# Using the Mechanical Bond to Tune the Performance of a Thermally Activated Delayed Fluorescence Emitter**

**DOI:** 10.1002/anie.202101870

**Published:** 2021-05-03

**Authors:** Pachaiyappan Rajamalli, Federica Rizzi, Wenbo Li, Michael A. Jinks, Abhishek Kumar Gupta, Beth A. Laidlaw, Ifor D. W. Samuel, Thomas J. Penfold, Stephen M. Goldup, Eli Zysman‐Colman

**Affiliations:** ^1^ Organic Semiconductor Centre EaStCHEM School of Chemistry University of St Andrews St Andrews Fife KY16 9ST UK; ^2^ Materials Research Centre Indian Institute of Science Bangalore 560012 India; ^3^ Chemistry University of Southampton Highfield Southampton SO17 1BJ UK; ^4^ Organic Semiconductor Centre SUPA School of Physics and Astronomy University of St Andrews St Andrews Fife KY16 9SS UK; ^5^ Chemistry, School of Natural and Environmental Sciences Newcastle University Newcastle upon Tyne NE1 7RU UK

**Keywords:** luminescence, mechanical bond, rotaxane, supramolecular chemistry, TADF

## Abstract

We report the characterization of rotaxanes based on a carbazole‐benzophenone thermally activated delayed fluorescence luminophore. We find that the mechanical bond leads to an improvement in key photophysical properties of the emitter, notably an increase in photoluminescence quantum yield and a decrease in the energy difference between singlet and triplet states, as well as fine tuning of the emission wavelength, a feat that is difficult to achieve when using covalently bound substituents. Computational simulations, supported by X‐ray crystallography, suggest that this tuning of properties occurs due to weak interactions between the axle and the macrocycle that are enforced by the mechanical bond. This work highlights the benefits of using the mechanical bond to refine existing luminophores, providing a new avenue for emitter optimization that can ultimately increase the performance of these molecules.

## Graphical abstract

We report rotaxanes containing a carbazole‐containing TADF luminophore in which the mechanical bond improves key photophysical properties, including the photoluminescence quantum yield and the singlet–triplet energy gap (Δ*E*_ST_). Computational simulations, supported by X‐ray crystallography, suggest this is due to weak interactions between the axle and macrocycle, enforced by the mechanical bond.

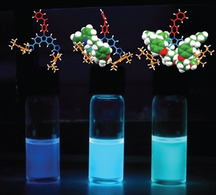

## Introduction

Organic compounds exhibiting thermally activated delayed fluorescence (TADF) have enjoyed tremendous recent attention due to their ability to undergo efficient spin state changes between the low‐lying excited states. This has led to TADF compounds being exploited as emitters in organic light emitting diodes (OLEDs),^[1]^ where they enable both singlet and triplet excitons to be harvested to achieve high efficiency, as well as photocatalysts for photoredox‐based organic transformations,^[2]^ sensors^[3]^ and as bio‐imaging agents.^[4]^

One of the major challenges in the optimization of TADF emitters is the inherent contradiction in the parameters that determine their photophysical properties.^[5]^ TADF is the result of two successive processes: reverse intersystem crossing (rISC) from the lowest excited triplet (T_1_) to the lowest excited singlet state (S_1_), followed by the emission from S_1_ to the ground state (S_0_). The efficiency of the former can be increased by decreasing the singlet‐triplet energy gap, Δ*E*_ST_, which is itself governed by the exchange integral between the HOMO and LUMO;^[1a]^ a smaller overlap of the electron density distributions of these two orbitals leads to a smaller Δ*E*_ST_. However, the rate of emission from S_1_ is proportional to the overlap between these two orbitals. Thus, upon first inspection, it appears paradoxical to maximize the efficiencies of both simultaneously.

The rate of rISC, characterised by the rate constant *k*_rISC_, increases exponentially as Δ*E*_ST_ decreases.^[1b]^ In purely organic TADF compounds, a small Δ*E*_ST_ is accomplished by spatially separating the orbitals involved in the lowest excited state which minimises the effect of Pauli repulsion (exchange) interactions.^[6]^ The most common molecular design to achieve this relies upon donor‐acceptor (D‐A) architectures, where the HOMO is localized on the donor and the LUMO is localized on the acceptor, resulting in S_1_ and T_1_ excited states that are predominantly intramolecular charge‐transfer (CT) in character.^[7]^ Several strategies to modulate the overlap between the two frontier molecular orbitals (FMOs) have been successfully applied, including inducing a large torsion between the D and A units by insertion of bulky substituents,[^5b^, ^6^, ^8^] inserting spiro‐junctions,^[9]^ and physically separating the FMOs through a homo‐junction that allows through‐space interaction of the HOMO and LUMO.^[10]^

The challenge of enhancing *k*_rISC_ by decreasing Δ*E*_ST_ whilst maintaining sufficient *k*_r_, to give high photoluminescence quantum yield, Φ_PL_, has motivated a significant amount of experimental^[11]^ and computational^[12]^ work focused on both elucidating and resolving the complex interplay between the photophysical properties of TADF materials. This has demonstrated, with the exception of multi‐resonance emitters,^[13]^ the importance of the rotational freedom around the D‐A bond to permit vibronic coupling between T_1_ and other low lying triplet excited states which aids rISC to the S_1_ state, combined with a near 90° mean dihedral angle between donor and acceptor to minimise Δ*E*_ST_.

Designing TADF emitters with the required fine structural control is not trivial. Many publications have focused on either using covalent modifications, such as substitution to enhance steric hindrance,^[14]^ or non‐covalent interactions^[15]^ to modify the conformational preferences of the D‐A bond. An alternative and as yet unexplored approach is to use the crowded, flexible environment of the mechanical bond in mechanically interlocked molecules (MIMs)^[16]^ such a rotaxanes to influence the properties of a TADF luminophore, although this approach has been used to influence the properties of other radiative processes.^[17]^ Indeed, the investigation of MIMs has expanded dramatically over the last half‐century, thanks largely to the development of high yielding, flexible synthetic methodologies^[16]^ that make them available for study in a range of areas including as sensors^[18]^ and catalysts,^[19]^ as well as their well‐known role as components of molecular machines.^[20]^ Herein we report a series of carbazole‐benzophenone‐based rotaxanes that demonstrate the ability of the environment provided by the macrocycles threaded close to the emitting core to fine‐tune the photophysical properties of a TADF‐active axle in solution and thin films.^[21]^

## Results and Discussion

### Synthesis of Interlocked TADF Emitters

**Figure 1:**
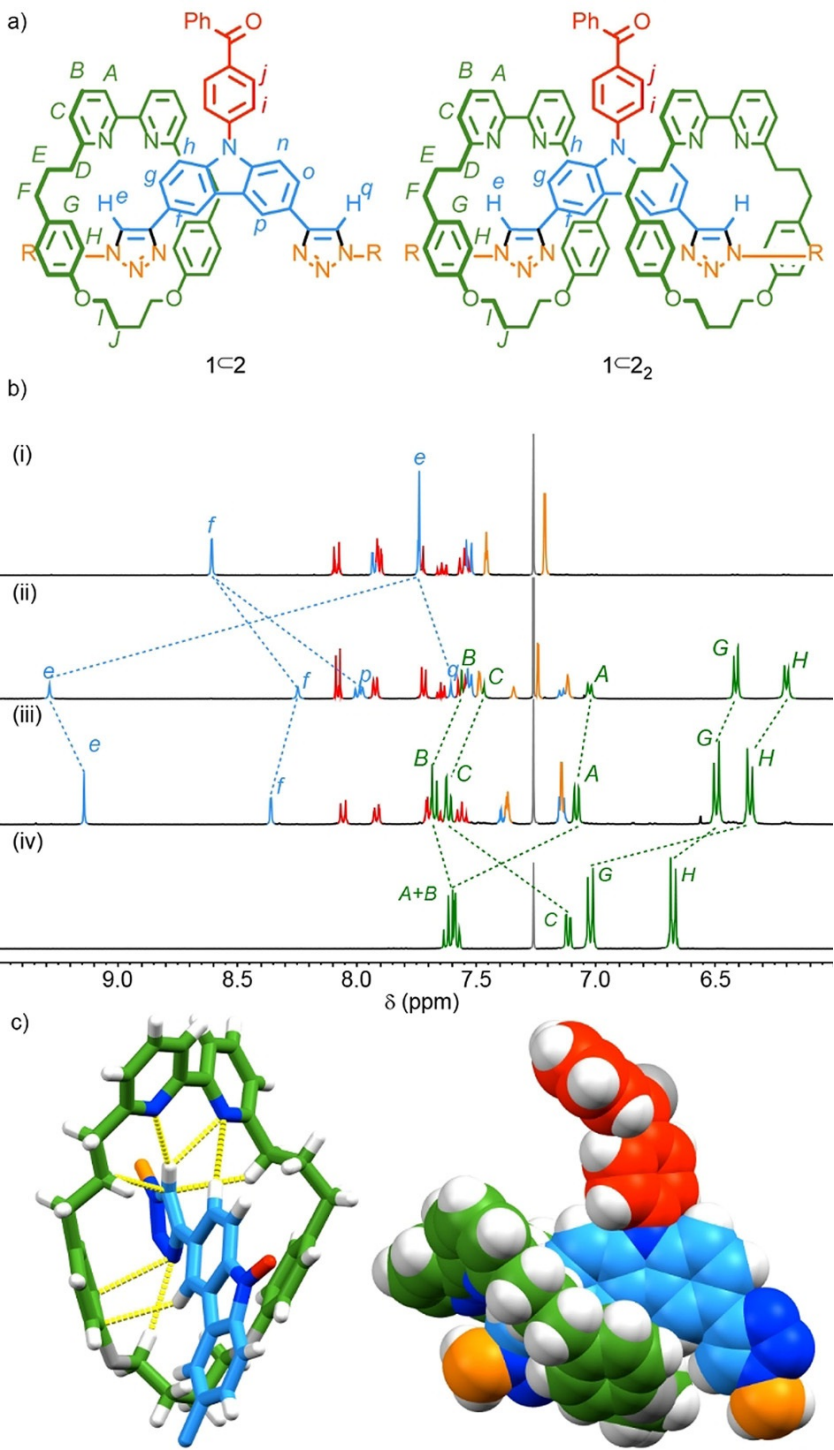
a) Interlocked TADF emitters [2]rotaxane **1**⊂**2** and [3]rotaxane **1**⊂**2**_2_. b) Partial ^1^H NMR (CDCl_3_, 400 MHz, 298 K) of (i) axle **1**, (ii) **1**⊂**2**, (iii) **1**⊂**2**_2_ and (iv) macrocycle **2**. Selected signals are assigned, and colour coded (see Supporting Information for labels). c) Solid‐state structure of **1**⊂**2**, in sticks representation with selected inter‐component interactions highlighted (left) and space‐filling representation highlighting the steric crowding of the mechanical bond (right).

The design of prototypical interlocked TADF emitters [2]rotaxane **1**⊂**2** and [3]rotaxane **1**⊂**2**_2_ (Figure 1 a) was based on the carbazole‐benzophenone system developed by Zysman‐Colman and co‐workers.^[22]^ The rotaxanes were synthesised in good yield using an active template^[23]^ Cu‐mediated azide‐alkyne cycloaddition (AT‐CuAAC)^[24]^ reaction between a benzophenone (BP) substituted carbazole (Cz) bis‐alkyne core and a bulky benzylic azide in the presence of macrocycle **2** ^[25]^ (see ESI for details, Scheme S2). Non‐interlocked axle **1** was synthesized using the CuAAC^[26]^ reaction in the absence of macrocycle **2**. ^1^H NMR analysis of the purified products revealed the expected differences between the non‐interlocked and interlocked emitters (Figure 1 b);^[24c]^ triazole (Tz) protons in regions of the axle encircled by a macrocycle (H_*e*_) are significantly deshielded relative to the non‐interlocked components and other resonances arising from both the encircled region of the axle and macrocycle components (e.g. H_*f*_, H_*G*_ and H_*H*_) are shifted to lower ppm.

Single‐crystal X‐ray diffraction analysis of [2]rotaxane **1**⊂**2** ^[37]^ suggests that, in keeping with previous reports,^[24c]^ the high ppm chemical shift of the encircled triazole protons is due to CH⋅⋅⋅N hydrogen bonds to the bipyridine N atoms (Figure 1 c). In addition, a network of weak C−H⋅⋅⋅π, C−H⋅⋅⋅N and C−H⋅⋅⋅O contacts between the macrocycle and axle are observed in the solid state. Viewed in a spacefill representation, it is clear that the macrocycle impinges significantly on the Cz fragment but does not interact with the BP unit, suggesting that any changes in the photophysical properties of the interlocked structures relative to the non‐interlocked axle are likely to arise from modulation of the properties of the donor unit.

### Photophysical Properties of 1, 1⊂2 and 1⊂2_2_

**Figure 2:**
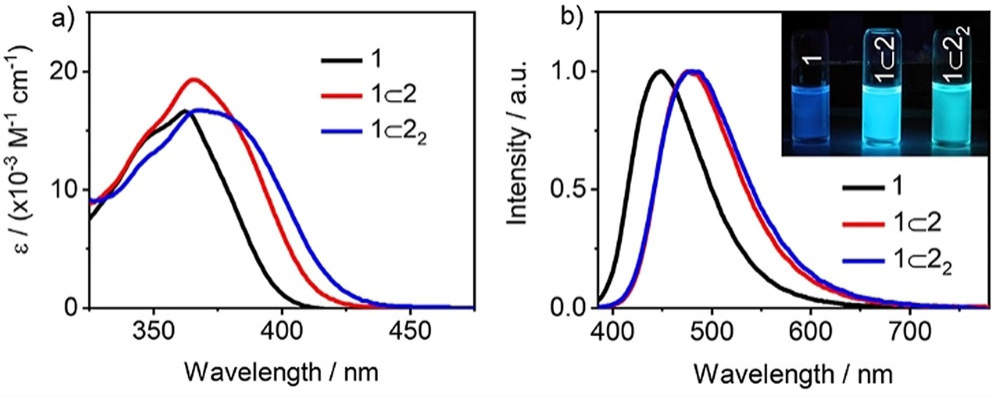
a) Absorption and b) PL spectra of **1**, **1**⊂**2** and **1**⊂**2_2_** in PhMe (*λ*_exc_=340 nm, 10^−5^ M). Inset: photograph of PhMe solutions (10^−5^ M) of the emitters under UV light.

All three emitters exhibited the expected CT UV/Vis absorption band, which is slightly and progressively red‐shifted with each additional macrocycle encircling the axle (Figure 2 a). The same trend was observed in the broad, steady‐state photoluminescence (PL) spectra, with a more pronounced shift in the emission maxima, *λ*_PL_, from 449 nm to 477 nm and 484 nm, for **1**, **1**⊂**2** and **1**⊂**2**_2_, respectively (Figure 2 b). A bathochromic shift was also observed in more polar solvents, consistent with the emissive excited state having significant CT character (Figure S39).^[27]^

**Figure 3:**
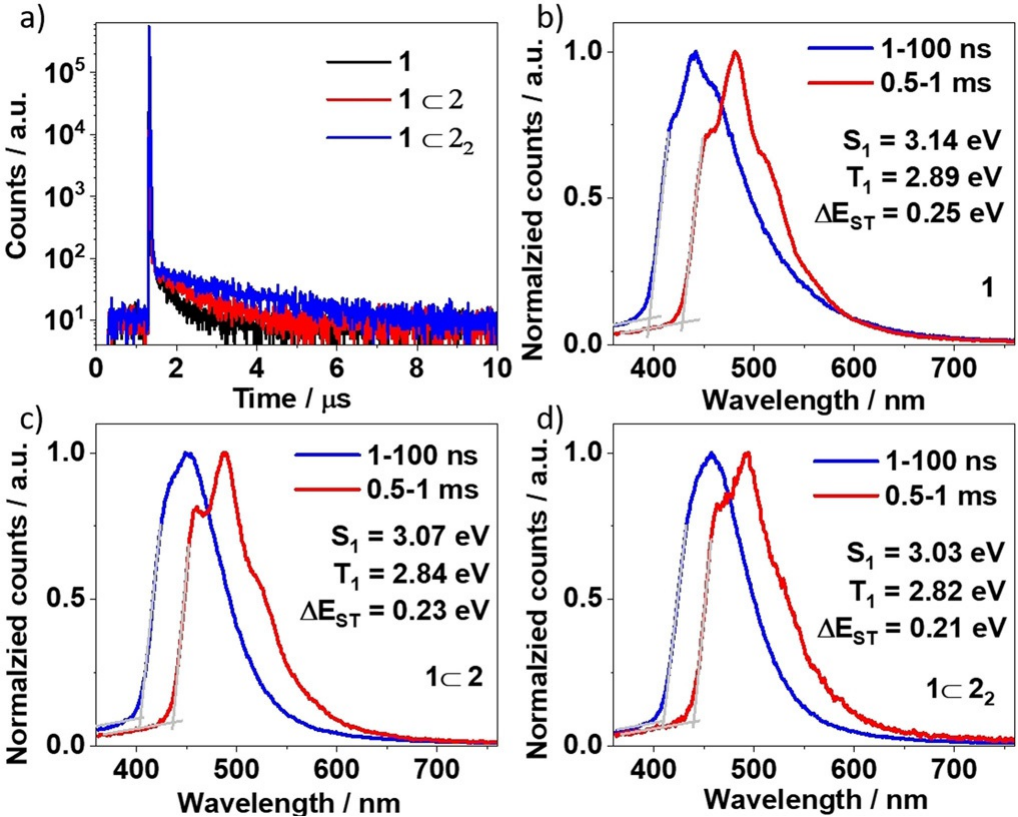
a) Time‐resolved PL decays and spectra of **1**, **1**⊂**2** and **1**⊂**2_2_** in PhMe at 298 K (*λ*_exc_=378 nm, 10^−5^ M). b,c,d) Prompt (1–100 ns) and phosphorescence (0.5–1 ms) spectra measured in PhMe (*λ*_exc_=343 nm, 10^−5^ M) at 77 K of **1**, **1**⊂**2** and **1**⊂**2**_2_.

Rotaxanes **1**⊂**2** and **1**⊂**2**_2_ displayed higher Φ_PL_ than the non‐interlocked axle **1** under both deoxygenated and ambient conditions (Table 1). Φ_PL_ was lower in the presence of O_2_ as is expected for TADF‐active compounds, for which accessible triplet states play a key role. Prompt, *τ*_p_, and delayed, *τ*_d_, fluorescence lifetimes were obtained from time‐resolved PL decays (Figure 3 a). The average *τ*_p_ and *τ*_d_ values both increase from **1** to **1**⊂**2** to **1**⊂**2**_2_ (Table 1). Prompt fluorescence for **1**⊂**2** and **1**⊂**2**_2_ at 77 K still retains a CT character, while the structured phosphorescence observed is clearly locally excited (^3^LE) in nature (Figures 3 b–d); the prompt fluorescence for **1** at 77 K shows a mixed CT/LE character. The differences in molecular orbital type between S_1_ and T_1_ implies that direct rISC is possible between these two states according to El Sayed's rule.^[28]^ The Δ*E*_ST_ values calculated from the difference between the onset of the prompt fluorescence and phosphorescence emission in spectra measured at 77 K decreased from 0.25 eV for **1** to 0.23 eV for **1**⊂**2** and 0.21 eV for **1**⊂**2**_2_.

**Table 1:** Optoelectronic properties of 1, 1⊂2 and 1⊂2_2_ in solution.

Entry		**1**	**1⊂2**	**1⊂2_2_**
	Photophysical measurements at 298 K			
1	*λ*_abs_ [nm]^[a]^	362	366	368
2	*λ*_PL_ [nm]^[b]^	449	477	484
3	Φ_PL_ (N_2_) [%]^[c]^	4	27	37
4	Φ_PL_ (air) [%]^[d]^	4	22	25
5	*τ*_p_ [ns]^[e]^	2.2	5.5	8.4
6	*τ*_d_ [μs]^[e]^	0.3	0.8	2.0
	Photophysical measurements at 77 K			
7	*λ*_PL_ [nm]^[f]^	440	450	460
8	*λ*_PL_ [nm]^[g]^	481	488	493
9	E(S_1_) [eV]^[h]^	3.14	3.07	3.03
10	E(T_1_) [eV]^[i]^	2.89	2.84	2.82
11	Δ*E*_ST_ [eV]^[j]^	0.25	0.23	0.21
	Electrochemistry measurements			
12	HOMO [eV]^[k]^	−5.62	−5.47	−5.41
13	LUMO [eV]^[l]^	−2.53	−2.52	−2.52

[a] UV/Vis absorption CT band maximum measured at 298 K in PhMe. [b] Steady‐state PL band maximum measured at 298 K in PhMe (*λ*_exc_=340 nm, 10^−5^ M). [c] Φ_PL_ measured in PhMe (*λ*_exc_=360 nm) under N_2_. [d] Φ_PL_ measured in PhMe (*λ*_exc_=360 nm) under air. [e] Average lifetime value obtained from time‐resolved PL decay spectra measured at 298 K in PhMe (*λ*_exc_=378 nm, 10^−5^ M). [f] Prompt fluorescence maximum measured at 77 K in PhMe (*λ*_exc_=343 nm, 10^−5^ M). [g] Phosphorescence maximum measured at 77 K in PhMe (*λ*_exc_=343 nm, 10^−5^ M). [h] Estimated from the onset of the prompt fluorescence spectrum measured at 77 K in PhMe. [i] Estimated from the onset of phosphorescence spectrum measured at 77 K. [j] Δ*E*_ST_=*E*(S_1_) − E(T_1_). [k] Determined from the oxidation potential observed by CV in MeCN (10^−3^ M). [l] Calculated from HOMO + *E*_g_.

Decreasing Δ*E*_ST_ is very desirable for TADF. The decrease across the series superficially appears to contradict the increase in *τ*_d_ as lower Δ*E*_ST_ is expected to increase *k*_rISC_. This apparent contradiction can be resolved by considering that emission lifetimes depend on both the rate of emission and the rate of non‐radiative decay, which both deplete the excited state population. The higher Φ_PL_ of **1**⊂**2** and **1**⊂**2**_2_ compared with **1** *despite* the longer measured lifetimes suggests that the mechanical bond serves to supress non‐radiative decay, outweighing the effect of any increase in *k*_rISC_ on the emission lifetime. This effect of the mechanical bond appears to extend to the photostability of the compounds; upon continuous irradiation at 325 nm of an aerated toluene solution the emission spectra of **1** evolved to one that contained a more pronounced LE character, which implies photodegradation of the emitter, whereas the emission profiles of rotaxanes **1**⊂**2** and **1**⊂**2**_2_ saw only a modest decrease in intensity over the same period (Figure S38).^[29]^

The HOMO and LUMO levels of **1**, **1**⊂**2** and **1**⊂**2**_2_ were determined by cyclic voltammetry (CV) and differential pulse voltammetry (DPV) in MeCN (Table 1). The Cz‐centred oxidation waves and BP‐centred reduction waves were found to be reversible for all three molecules (Figure S39). The oxidation potentials, *E*_1/2_^ox^, for **1** (1.20 V), **1**⊂**2** (1.05 V) and **1**⊂**2**_2_ (0.99 V) and the corresponding trend in the HOMO levels are consistent with the bathochromic shift observed in both the absorption and PL spectra. The reduction potentials, *E* ^red^, for **1** (−1.72 V), **1**⊂**2** (−1.74 V) and **1**⊂**2**_2_ (−1.75 V) are very similar, suggesting that the LUMO levels are largely unaffected by the mechanical bond, consistent with the solid‐state structure of **1**⊂**2** that suggests there is little interaction between the BP acceptor with the macrocycle components of the rotaxanes.

### Computational Modelling of 1, 1⊂2 and 1⊂2_2_

**Figure 4:**
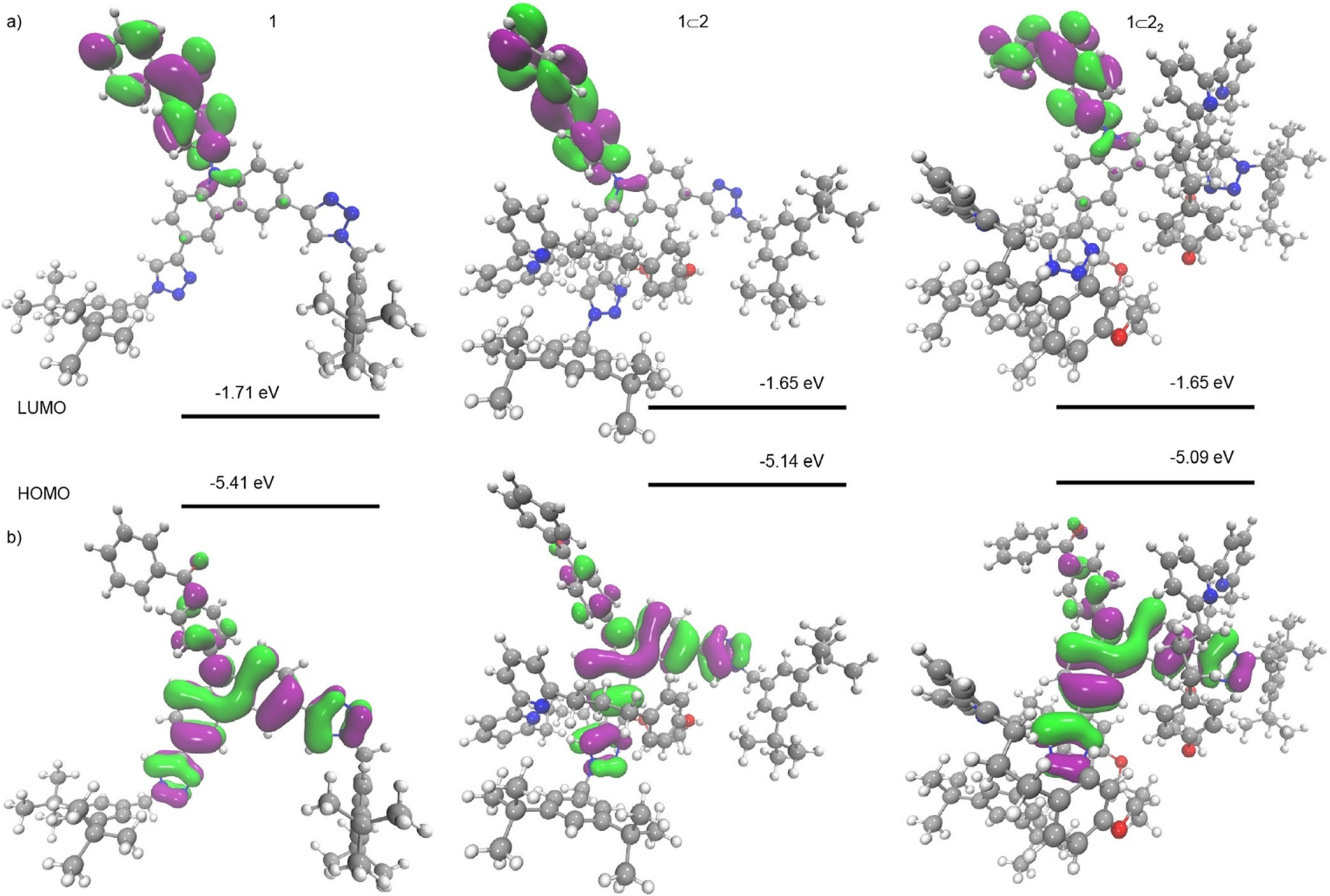
The HOMO (top) and LUMO (bottom) orbitals for **1**, **1**⊂**2** and **1**⊂**2_2_** at the ground state geometry computed using DFT(PBE0)/Def2‐SVP.

To aid in the interpretation of the photophysical and electrochemical data, and shed light on the role of the mechanical bond in determining the properties of **1**, **1**⊂**2** and **1**⊂**2**_2_, density functional theory (DFT) and ab initio molecular dynamics (MD) simulations were performed using the Q‐Chem^[^30^]^ and Terachem^[31]^ softwares, respectively. Consistent with previous results,^[22]^ DFT (PBE0)^[32]^ analysis of the lowest energy conformer of the ground state (S_0_) structures found the HOMO to be centred on the Cz donor moiety in all three cases, with the LUMO centred on the BP acceptor unit (Figure 4). In all structures, both triazole moieties were also found to contribute to the HOMO. The calculated HOMO/LUMO energy levels (Table 2) agree well with those measured by electrochemistry and, importantly, reproduce the trend for an increasing red‐shift in the CT absorption maximum (*λ*_abs_ **1**>**1**⊂**2**>**1**⊂**2**_2_).

**Table 2:** DFT(PBE0)/Def2‐SVP calculated electronic properties of axle **1**, **1⊂2_2_** and **1⊂2_2_** at both the optimised ground and excited S_1_ state geometries.

Entry		**1**	**1⊂2**	**1⊂2_2_**
	Ground state geometry			
1	HOMO [eV]	−5.41	−5.14	−5.09
2	LUMO [eV]	−1.71	−1.65	−1.65
3	S_1_ (*f*) [eV]^[a]^	3.17 (0.316)	3.00 (0.272)	2.91 (0.271)
4	T_1_ [eV]	2.83	2.70	2.63
5	T_2_ [eV]	3.11	3.10	3.10
	Excited state (S_1_) geometry			
6	S_1_ (*f*) [eV]^[a]^	2.33 (0.000)	2.06 (0.000)	1.99 (0.000)
7	T_1_ [eV]	2.32	2.06	1.99
8	T_2_ [eV]	2.74	2.78	2.79

[a] Values in parentheses refer to the oscillator strength of the S_0_–S_1_ transition.

The calculations also shed light on the origin of this effect. Whereas the LUMO energy levels are largely unaffected by the mechanical bond, the HOMO is destabilised due to a hydrogen bond between the bipyridine N donors and the C−H of the triazole, as observed in the solid‐state structure of **1**⊂**2**. This serves to increase the donation of electron density from the triazole units to the carbazole core and accounts for the large difference in HOMO level (0.27 eV) between **1**, which lacks this interaction, and **1**⊂**2**, and also the less pronounced difference (0.06 eV) between **1**⊂**2** and **1**⊂**2**_2_.

Bond lengths and angles in the S_0_ state were remarkably similar for all three emitters. The Cz‐BP bond was found to vary between 1.403 and 1.406 Å and minima at 40° and 140° were found when the Cz‐BP dihedral was scanned, with a relatively low barrier (≈0.1 eV) to conformational exchange (Figure S48). The most striking structural difference between **1**⊂**2** and **1**⊂**2**_2_ is that the Tz units of the latter adopt a *syn*‐*syn* orientation whereas those of **1**⊂**2** preferentially adopt a *syn*‐*anti* arrangement, the *syn* oriented unit being that encircled by the macrocycle.^[33]^ This difference can be explained by observing that the *syn* conformation of Tz units minimises steric repulsion between an encircling macrocycle and the Cz unit. A *syn*‐*anti* preference was also observed in the case of axle **1** as this minimises repulsion between the dipoles associated with the Tz rings.^[34]^ In all cases the Cz‐Tz distance was found to be identical (1.46 Å).

Models of the excited S_1_ state revealed a number of important changes. Firstly, in all cases, the Cz‐BP dihedral angle of the lowest energy conformation was found to be ≈90° and the Cz‐BP bond lengthened (1.44 Å), consistent with a slight weakening of this bond, as is typically observed in D‐A TADF emitters.^[35]^ In the excited state structures of **1** and **1**⊂**2**, one of the Cz‐Tz bonds was found to contract (Δ=0.022 and 0.029 Å, respectively) while the other remained largely unchanged, causing the electron density on the donor to exhibit a slight asymmetry. In contrast the Cz‐Tz bonds of **1**⊂**2**_2_ both contracted but remained very similar (1.442 and 1.447 Å). In all cases, both Cz‐Tz dihedral angles were reduced in S_1_ compared with S_0_, consistent with increased donation from the Tz moieties to the Cz core in the excited state.

In the ground state geometry, time dependent‐DFT (TD‐DFT) calculations of **1** identified two triplet states below the S_1_ energy level, both of which exhibit a mixed ^3^CT and acceptor‐based ^3^LE character (Table 2). For **1**⊂**2** and **1**⊂**2**_2_ the acceptor‐based ^3^LE appears slightly above the S_1_ state. This presence of a ^3^LE state is common for high performance TADF emitters^[11a]^ and consistent with the structured luminescence spectra (Figure 3). At the excited state geometry, TD‐DFT calculations found that the lowest S_1_ and T_1_ states exhibit pure CT character. Although the ^3^LE triplet state lies ≈0.3 eV higher in energy, it is expected that this gap is overestimate due to the challenges associated with TD‐DFT calculations predicting the absolute energy of CT states.^[36]^ The oscillator strength (*f*) for the S_1_‐S_0_ transition was calculated to be 0.316, 0.272 and 0.271 for **1**, **1**⊂**2** and **1**⊂**2**_2_ respectively at the ground state geometry but fell to 0 for all structures in their computed excited state geometry. Given that we experimentally observed photoluminescence with a μs lifetime in all cases, we set out to explore the role of molecular flexibility on the photophysical properties using ab initio MD simulations.

Simulations were performed over 10 ps (Figure S47) in both the ground (S_0_) and emissive excited state (S_1_) to investigate the dynamic properties of the emitters. In keeping with the DFT results, comparable average values of D‐A bond length were found for **1**, **1**⊂**2** and **1**⊂**2**_2_ in both the ground and excited states, confirming this value is unaffected by the mechanical bond. Similarly, in the ground state, peaks were found in the distribution of Cz‐BP dihedral angles at ≈40° and 140°, in keeping with the low barrier to conformational exchange predicted above. In contrast, in the excited state, the distribution of the Cz‐BP dihedral angle is subtly influenced by the interlocked macrocycles, indicated by a mean and standard deviation of 89±17°, 90±14° and 90±11°, observed for **1**, **1**⊂**2** and **1**⊂**2**_2_, respectively. Although subtle, the reduced standard deviation observed upon adding the macrocycle is indicative of a slight rigidification of this conformational mode due to the ability of the encircling macrocycles to exert fine conformational control of the excited state dynamics. A similar effect is also observed for the dihedral angle between the Cz and Tz moieties, which is 3±16° for **1**, 2±11° (encircled) and 5±17° (free) for **1**⊂**2** and 2±6° for **1**⊂**2_2_**. Note, for **1**⊂**2** we report two dihedral angles and only the one encircled by the macrocycle is altered relative to the axle alone.

The cumulative effect of the subtle dynamic differences enforced by the mechanical bond can be observed in the key parameters defining TADF performance, namely Δ*E*_ST_ and the average oscillator strength for the S_1_→S_0_ transition (*f_S1_*). Firstly, the trend in the mean Cz‐BP dihedral angles found by MD leads to an energy gap between the ^1^CT and ^3^CT states, which follows the trend **1**>**1**⊂**2**>**1**⊂**2**_2_ as angles closer to 90° are associated with reduced splitting between states of the same character. Although the reduction of Δ*E*_ST_ observed experimentally is between the ^1^CT and ^3^LE and due to the stronger electron donating strength introduced by the rotaxanes, the reduced gap between the two CT states is still expected to increase *k*_rISC._ ^[12b]^ Secondly, narrowing of the distribution of dihedral angles around 90° for Cz‐BP bond is expected to decrease the overall oscillator strength for the S_1_→S_0_ transition. Indeed, the average values of *f_S1_* determined by ab initio MD are 0.0260, 0.0095 and 0.0088 for **1**, **1**⊂**2** and **1**⊂**2**_2_ respectively, meaning that although the rotaxanes have reduced Δ*E*_ST_, they retain enough oscillator strength to be efficient emitters. Finally, the more conformationally restricted molecular framework, as indicated by narrower distribution of the Cz‐BP and Cz‐Tz dihedral angles, could also be expected to decrease the rate of non‐radiative decay and so lead to a higher photoluminescence quantum yield, consistent with the **1**>**1**⊂**2**>**1**⊂**2**_2_ trend observed.

### Luminescent Properties of 1, 1⊂2 and 1⊂2_2_ in Thin Films

We next performed a preliminary photophysical investigation to determine whether the effect of the mechanical bond was maintained in thin films. Spin‐coated 10 wt % doped films were prepared from chlorobenzene solutions of the three emitters with poly(methyl methacrylate) (PMMA) (Table 3).

**Table 3:** Photophysical measurements in films.

Entry		**1**	**1⊂2**	**1⊂2_2_**
	10 wt % PMMA			
1	λ_PL_ [nm]^[a]^	450	458	463
2	Φ_PL_ (N_2_) [%]^[b]^	6	23	29
3	Φ_PL_ (air) [%]^[c]^	5	18	21
4	*τ*_p_ [ns]^[d]^	5.0	5.4	6.3
5	*τ*_d_ [μs]^[d]^	94	152	206

[a] Steady‐state PL band maximum measured at 77 K of 10 wt % PMMA film (*λ*_exc_=343 nm). [b] Absolute Φ_PL_ of 10 wt % PMMA film measured using an integrating sphere under N_2_ atmosphere. [c] Absolute Φ_PL_ of 10 wt % PMMA film measured using an integrating sphere under air. [d] Average lifetime value obtained from time‐resolved PL decay spectra measured at 298 K of 10 wt % PMMA film (*λ*_exc_=378 nm).

**Figure 5:**
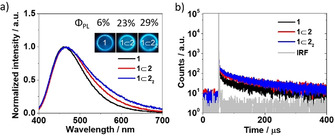
a) Emission spectra of **1**, **1**⊂**2** and **1**⊂**2_2_** in 10 wt % PMMA film (*λ*_exc_=343 nm). b) Time‐resolved emission spectra of **1**, **1**⊂**2** and **1**⊂**2_2_** in 10 wt % PMMA film (*λ*_exc_=378 nm).

Although the emission of the **1**, **1**⊂**2** and **1**⊂**2**_2_ in PMMA films was blue‐shifted compared to the PL spectra in PhMe, a similar red‐shift was also observed along the series (Figure 5 a), which suggests that the hydrogen bonds between the bipyridine unit and the Tz moieties are maintained in the film. The shift in emission wavelength increased incrementally with each additional interlocked macrocycle. The PL spectra of the PMMA films are sharper than those in solution, as would be expected in a rigid medium that reduces both conformational freedom and inhibits reorganization of the system. As with the solution‐state measurements, τ_d_ progressively increases from **1** to **1**⊂**2** and **1**⊂**2**_2_ (Figure 5 b).

Finally, the effect of the mechanical bond was maintained in different host media. Replacing PMMA with bis[2‐(diphenylphosphino)phenyl] ether oxide (DPEPO) or 1,3‐bis(*N*‐carbazolyl)benzene (mCP) gave qualitatively similar results (see ESI for details); a progressive red‐shift was observed across both series, with a corresponding decrease in Δ*E*_ST_. TADF was observed in all hosts.

## Conclusion

We have designed and synthesized a series of carbazole‐benzophenone‐based TADF emitters, two of which are mechanically interlocked. The electrochemical and photophysical properties of these molecules were mainly investigated in solution, with the conclusion that the mechanical bond can tune important properties of such TADF emitters. First, we find that the mechanical bond leads to a large increase in photoluminescence quantum yield, which is a very important property for light‐emitting materials. Second, we find that the mechanical bond reduces Δ*E*_ST_, which is highly desirable for TADF OLED emitters because it facilitates harvesting of triplet excitons. Third, the mechanical bond increases the photostability of the emitters under continuous UV excitation. Fourth, the mechanical bond provides a way of fine‐tuning the HOMO energy level, making it shallower, an adjustment that would facilitate hole injection in OLEDs. Fifth, we find that it leads to fine‐tuning of the emission spectrum, moving it slightly to the red, thereby providing a way of optimising the emission colour. Our DFT and ab initio MD simulations show that the fine‐tuning of the HOMO energy, shift in emission energy and reduction in the flexibility of the molecule is caused by weak interactions between the bipyridine N donors and the C−H of the triazole. They also demonstrate that the mechanical bond subtly alters the conformation of the TADF‐active axle, which reduces the energy gap between the two CT states and decreases the oscillator strength of the S_1_ state, key parameters defining TADF performance. The effects on the TADF parameters were maintained in thin films, reinforcing the hypothesis that the mechanical bond can be exploited to optimize the efficiency of such emitters.

Given the exceptional interest TADF emitters have recently gained, combined with increasing availability of rotaxanes and other mechanically interlocked molecules, the use of the mechanical bond to engineer existing and novel luminophores is a promising approach for TADF emitter optimization and, ultimately, a new approach for increasing device efficiency. In addition, given that modern computational chemistry is able to simulate the properties of these large structures to predict trends and provide insights on the origin of proposed effects, it should be possible to generate predictive data for future targets.

The research data underpinning this publication can be accessed at https://doi.org/10.17630/e7f5c0f6‐7acd‐4594‐8972‐a095170ca213.

## Conflict of interest

The authors declare no conflict of interest.

## Supporting information

As a service to our authors and readers, this journal provides supporting information supplied by the authors. Such materials are peer reviewed and may be re‐organized for online delivery, but are not copy‐edited or typeset. Technical support issues arising from supporting information (other than missing files) should be addressed to the authors.

Supplementary

Supplementary

Supplementary

Supplementary

Supplementary

Supplementary
