# Supplementary material for: Using the Mechanical Bond to Tune the Performance of a Thermally Activated Delayed Fluorescence Emitter
Source: Angew Chem Int Ed Engl. 2021 May 3;60(21):12066–73. doi: 10.1002/anie.202101870 (PMC8251797; doi:10.1002/anie.202101870)

checkCIF/PLATON report

You have not supplied any structure factors. As a result the full set of tests cannot be run.

THIS REPORT IS FOR GUIDANCE ONLY. IF USED AS PART OF A REVIEW PROCEDURE FOR PUBLICATION, IT SHOULD NOT REPLACE THE EXPERTISE OF AN EXPERIENCED CRYSTALLOGRAPHIC REFEREE.

No syntax errors found.   CIF dictionary   Interpreting this report

Datablock: mj_305

```
Bond precision:  C-C = 0.0028 A          Wavelength=0.71073

Cell:            a=16.7366(5)     b=14.4586(3)       c=31.8111(6)
                 alpha=90         beta=93.707(2)     gamma=90
Temperature:     293 K
```

	Calculated	Reported
Volume	7681.8(3)	7681.8(3)
Space group	P 21/n	P 1 21/n 1
Hall group	-P 2yn	-P 2yn
Moiety formula	C59 H63 N7 O, C32 H34 N2 O2 [+ solvent]	4(C32 H34 N2 O2), 4(C59 H63 N7 O)
Sum formula	C91 H97 N9 O3 [+ solvent]	C364 H388 N36 O12
Mr	1364.78	5459.36
Dx,g cm-3	1.180	1.180
Z	4	1
Mu (mm-1)	0.072	0.069
F000	2920.0	2533.0
F000’	2921.04	
h,k,lmax	24,21,46	22,20,44
Nref	24753	20856
Tmin,Tmax	0.998,0.999	0.671,1.000
Tmin’	0.988	

```
Correction method= # Reported T Limits: Tmin=0.671 Tmax=1.000
AbsCorr = MULTI-SCAN

Data completeness= 0.843          Theta(max)= 31.130

R(reflections)= 0.0624( 12665)    wR2(reflections)= 0.1773( 20856)

S = 1.036                  Npar= 940
```

The following ALERTS were generated. Each ALERT has the format
test-name_ALERT_alert-type_alert-level.
Click on the hyperlinks for more details of the test.

Alert level B

```
PLAT230_ALERT_2_B Hirshfeld Test Diff for    C02M     --C02P     .        7.3 s.u.
```

Author Response: Differences in the components of the anisotropic displacement paramete

---

Alert level C

```
ABSMU01_ALERT_1_C  The ratio of given/expected absorption coefficient lies
               outside the range 0.99 <> 1.01
            Calculated value of mu =     0.072
            Value of mu given      =     0.069
PLAT220_ALERT_2_C NonSolvent   Resd 1  C   Ueq(max)/Ueq(min) Range        3.6 Ratio
PLAT222_ALERT_3_C NonSolvent Resd 1  H   Uiso(max)/Uiso(min) Range        4.4 Ratio
PLAT230_ALERT_2_C Hirshfeld Test Diff for    C01Q     --C02Q     .        5.3 s.u.
```

Author Response: Differences in the components of the anisotropic displacement paramete

```
PLAT230_ALERT_2_C Hirshfeld Test Diff for    C020     --C02P     .        6.6 s.u.
```

Author Response: Differences in the components of the anisotropic displacement paramete

---

Alert level G

```
PLAT013_ALERT_1_G N.O.K.   _shelx_hkl_checksum Found in CIF ......     Please Check
PLAT042_ALERT_1_G Calc. and Reported MoietyFormula Strings  Differ     Please Check
PLAT045_ALERT_1_G Calculated and Reported Z Differ by a Factor ...       4.00 Check
PLAT068_ALERT_1_G Reported F000 Differs from Calcd (or Missing)...     Please Check
PLAT199_ALERT_1_G Reported _cell_measurement_temperature ..... (K)        293 Check
PLAT200_ALERT_1_G Reported   _diffrn_ambient_temperature ..... (K)        293 Check
PLAT605_ALERT_4_G Largest Solvent Accessible VOID in the Structure         84 A**3
PLAT720_ALERT_4_G Number of Unusual/Non-Standard Labels ..........        199 Note
PLAT802_ALERT_4_G CIF Input Record(s) with more than 80 Characters          1 Info
PLAT941_ALERT_3_G Average HKL Measurement Multiplicity ...........        3.7 Low
PLAT950_ALERT_5_G Calculated (ThMax) and CIF-Reported Hmax Differ           2 Units
PLAT952_ALERT_5_G Calculated (ThMax) and CIF-Reported Lmax Differ           2 Units
```

---

0 **ALERT level A** = Most likely a serious problem - resolve or explain
1 **ALERT level B** = A potentially serious problem, consider carefully
5 **ALERT level C** = Check. Ensure it is not caused by an omission or oversight
12 **ALERT level G** = General information/check it is not something unexpected

7 ALERT type 1 CIF construction/syntax error, inconsistent or missing data

```
   4 ALERT type 2 Indicator that the structure model may be wrong or deficient
   2 ALERT type 3 Indicator that the structure quality may be low
   3 ALERT type 4 Improvement, methodology, query or suggestion
   2 ALERT type 5 Informative message, check
```

Validation response form

Please find below a validation response form (VRF) that can be filled in and pasted into your CIF.

```
# start Validation Reply Form
_vrf_ABSMU01_mj_305
;
PROBLEM: The ratio of given/expected absorption coefficient lies
RESPONSE: ...
;
_vrf_PLAT220_mj_305
;
PROBLEM: NonSolvent   Resd 1  C   Ueq(max)/Ueq(min) Range        3.6 Ratio
RESPONSE: ...
;
_vrf_PLAT222_mj_305
;
PROBLEM: NonSolvent Resd 1  H   Uiso(max)/Uiso(min) Range        4.4 Ratio
RESPONSE: ...
;
# end Validation Reply Form
```

It is advisable to attempt to resolve as many as possible of the alerts in all categories. Often the minor alerts point to easily fixed oversights, errors and omissions in your CIF or refinement strategy, so attention to these fine details can be worthwhile. In order to resolve some of the more serious problems it may be necessary to carry out additional measurements or structure refinements. However, the purpose of your study may justify the reported deviations and the more serious of these should normally be commented upon in the discussion or experimental section of a paper or in the "special_details" fields of the CIF. checkCIF was carefully designed to identify outliers and unusual parameters, but every test has its limitations and alerts that are not important in a particular case may appear. Conversely, the absence of alerts does not guarantee there are no aspects of the results needing attention. It is up to the individual to critically assess their own results and, if necessary, seek expert advice.

Publication of your CIF in IUCr journals

A basic structural check has been run on your CIF. These basic checks will be run on all CIFs submitted for publication in IUCr journals (*Acta Crystallographica*, *Journal of Applied Crystallography*, *Journal of Synchrotron Radiation*); however, if you intend to submit to *Acta Crystallographica Section C* or *E* or *IUCrData*, you should make sure that full publication checks are run on the final version of your CIF prior to submission.

Publication of your CIF in other journals

Please refer to the *Notes for Authors* of the relevant journal for any special instructions relating to CIF submission.

PLATON version of 05/12/2020; check.def file version of 05/12/2020

Datablock mj_305 - ellipsoid plot

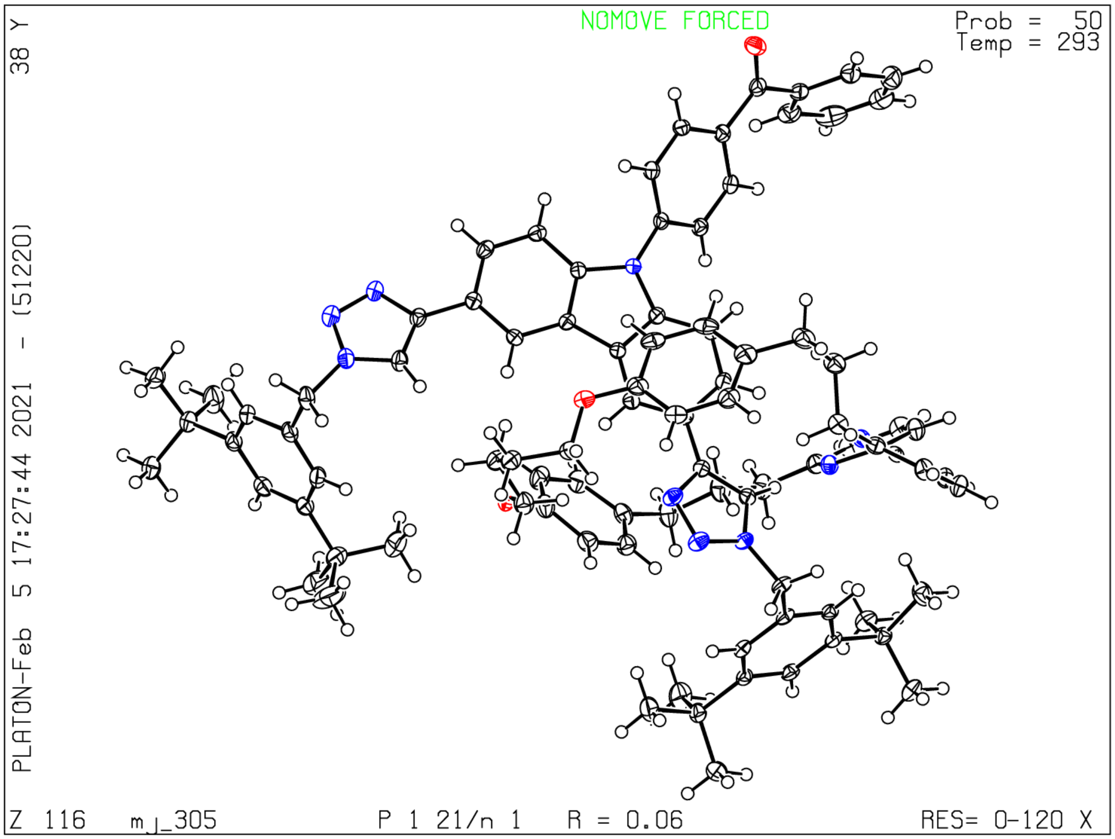

Supplement: Supplementary file 4 — Supplementary [file ANIE-60-12066-s005.pdf]
