# Supplementary material for: Using the Mechanical Bond to Tune the Performance of a Thermally Activated Delayed Fluorescence Emitter
Source: Angew Chem Int Ed Engl. 2021 May 3;60(21):12066–73. doi: 10.1002/anie.202101870 (PMC8251797; doi:10.1002/anie.202101870)
Supplement: Supplementary file 5 — Supplementary [file ANIE-60-12066-s006.pdf]

Angewandte Chemie
Eine Zeitschrift der Gesellschaft Deutscher Chemiker

Supporting Information

Using the Mechanical Bond to Tune the Performance of a Thermally Activated Delayed Fluorescence Emitter**

Pachaiyappan Rajamalli+, Federica Rizzi+, Wenbo Li+, Michael A. Jinks, Abhishek Kumar Gupta, Beth A. Laidlaw, Ifor D. W. Samuel, Thomas J. Penfold,* Stephen M. Goldup,* and Eli Zysman-Colman**

anie_202101870_sm_miscellaneous_information.pdf

Supporting Information

Table of Contents

General Experimental Information 2
Synthetic Schemes 3
3,6-Diiodo-9H-carbazole S3 4
4-(3,6-Dibromo-9H-carbazol-9-yl)-phenyl-methanone S4 6
(4-(3,6-bis(3,3-dimethylbut-1-yn-1-yl)-9H-carbazol-9-yl)phenyl)(phenyl)methanone S5 9
(4-(3,6-diethynyl-9H-carbazol-9-yl)phenyl)(phenyl)methanone S6 12
(4-(3-(1-(3,5-di-tert-butylbenzyl)-1H-1,2,3-triazol-4-yl)-6-ethynyl-9H-carbazol-9-yl)phenyl)(phenyl)methanone S7 15
Synthesis of Axle 1 18
Synthesis of [2]rotaxane $1\subset2$ 21
Synthesis of [3]rotaxane $1\subset2_2$ 24
Electrochemical Properties of the Emitters 27
Photophysical Properties 29
Computational Photophysical Properties of the Emitters 31
Single crystal X-ray crystallographic data for $1\subset2$ 33
References 36

General Experimental Information

Unless otherwise stated, all reagents were purchased from commercial sources (Acros Organics, Alfa Aesar, Fisher Scientific, FluoroChem, Sigma Aldrich and VWR) and used without further purification. $[Cu(NCMe)_4]PF_6$ was prepared as described by Pigorsch and Köckerling.[1] Anhydrous solvents were purchased from Acros Organics. Petrol refers to the fraction of petroleum ether boiling in the range 40-60°C. DIPEA refers to *N,N*-diisopropylethylamine. DMF refers to dimethylformamide. EDTA-NH_3 solution refers to a saturated aqueous solution of NH_3 (17% w/v) saturated with sodium-ethylenediaminetetraacetate. THF refers to tetrahydrofuran. $CDCl_3$ (without stabilising agent) was distilled over $CaCl_2$ and K_2CO_3 prior to use. All reactions were carried out under an atmosphere of air using bench grade solvents unless otherwise stated. Flash column chromatography was performed using Biotage Isolera-4 or Isolera-1 automated chromatography system. SiO_2 cartridges were purchased commercially from Biotage (SNAP or ZIP, 50 µm irregular silica, default flow rates). Neutralised SiO_2 refers to ZIP cartridges which were eluted with petrol-NEt_3 (99:1, 5 column volumes), followed by petrol (5 column volumes). Analytical TLC was performed on pre-coated silica gel plates on aluminum (0.25 mm thick, 60F254, Merck, Germany) and observed under UV light (254 nm).

NMR spectra were recorded on Bruker AV400 or AV500 instrument, at a constant temperature of 298 K. Chemical shifts are reported in parts per million from low to high field and referenced to residual solvent. Coupling constants (*J*) are reported in Hertz (Hz). Standard abbreviations indicating multiplicity were used as follows: m = multiplet, t = triplet, d = doublet, s = singlet, dd = doublet of doublets, app. = apparent, br = broad. Signal assignment was carried out using 2D NMR methods (COSY, HSQC, HMBC) where necessary. In some cases, complex multiplets with multiple contributing proton signals, exact assignment was not possible. Here indicative either/or assignments (e.g., $H_{A/B}$ for H_A or H_B) are provided. In interlocked compounds, all proton signals corresponding to axle components are in lower case, and all proton signals corresponding to the macrocycle components are in upper case. Low resolution mass spectrometry was carried out by the mass spectrometry services at the University of Southampton using a Waters TQD mass spectrometer equipped with a triple quadrupole analyser with UHPLC injection (BEH C18 column, MeCN-H_2O gradient with 0.2% formic acid). High resolution mass spectrometry was carried out by the mass spectrometry services at the University of Southampton using either a solariX Bruker Daltonics FT-ICR equipped with a 4.7 T superconducting magnet (samples were infused *via* a syringe driver at a flow rate of 5uL/minute; mass spectra were recorded using positive/negative ion atmospheric pressure photoionization [APPI]) or a MaXis Bruker Daltonics, with a Time of Flight (TOF) analyser (samples were introduced to the mass spectrometer via a Dionex Ultimate 3000 autosampler and uHPLC pump in a gradient of 20% MeCN in hexane to 100% MeCN [0.2% formic acid] over 5-10 min at 0.6 mL/min; Waters column: Acquity UPLC BEH C18 1.7 micron 50 × 2.1 mm; mass spectra were recorded using positive/negative ion electrospray ionisation).

Theoretical Calculations

Models for **1**, **1**⊂**2** and **1**⊂**2**$_2$ were prepared from crystal structures and subsequently optimized. For **1**⊂**2** and **1**⊂**2**$_2$ the position of the rotaxane relative to the axle was rotated into a number of different starting configurations to ensure the lowest energy conformer was obtained. All ground state optimizations have been carried using the Density Functional Theory (DFT) level as implemented within the Q-Chem quantum chemistry package,[2] within the approximation of the PBE0 functional with the def2-SVP basis set.[3,4] Excited state calculations were performed using Time-Dependent DFT (TD-DFT) with the Tamm-Dancoff approximation (TDA) [5,6] using the same functional basis set. The *Ab initio* molecular dynamics (AIMD) were performed using the TeraChem[7] software using DFT in the electronic ground state, and LR-TDDFT in the excited S_1 state. Throughout the PBE0[3] functional and def2-SVP[4] basis set were used. The trajectory was propagated using the velocity Verlet algorithm and a finite temperature of 300 K. After an initial equilibration period of 5 ps starting from the ground state or S_1 state optimized geometry, the MD were run for a further 10 ps from which all properties were calculated. All calculations included the solvent environment was described using a conductor-like polarisable continuum model using the dielectric constant of toluene.

Photophysical Measurements

The sample (solution/film) for the photophysical studies were prepared in spectroscopy-grade solvents. Absorption spectra of these emitters were recorded at room temperature using a Shimadzu UV-1800 double beam spectrophotometer. A stock solution with absorbance of ca. 0.5 was prepared and then four dilutions were prepared, and the Beer–Lambert law was found to be linear at the concentrations of these solutions. Samples for the steady-state and time-resolved emission were prepared in toluene at 10^{-5} M and degassed *via* freeze-pump-thaw cycles (three) using a quartz cuvette designed in-house. Steady-state emission and excitation spectra and time-resolved emission spectra were recorded at 298 K using an Edinburgh Instruments FLS980 fluorimeter. All samples for steady-state measurements were excited at 340 nm using a xenon lamp and time-resolved measurements were excited at 375 nm using a PicoQuant, LDH-D-C-375 pulsed diode laser. The thin films were prepared by spin-coating the samples from a solution of chlorobenzene of the desired sample on a sapphire substrate. Solid-state Φ_{PL} measurements were performed in an integrating sphere[8] under a nitrogen atmosphere in a Hamamatsu C9920-02 luminescence measurement system and films were made on sapphire substrates. Variable temperature (298 K, 200 K, 100 K, 77 K) measurements were done in a cryostat (Oxford Instruments). The excitation source was a picosecond laser emitting at 375 nm (PicoQuant, LDH-D-C-375) triggered by a delay generator (Stanford Research Systems, DG645) in burst mode. The burst mode was used to increase the excitation power so as to get more emitted photons from the samples and speed up the measurement. Film samples were prepared on sapphire substrates and cooled down to 77 K in a cold finger cryostat (Oxford Instruments). The samples were photoexcited using a femtosecond laser emitting at 343 nm (Orpheus-N, model: PN13F1). Emission from the samples was focused onto a spectrograph (Chromex imaging, 250is spectrograph) and detected with a sensitive gated iCCD camera (Stanford Computer Optics, 4Picos) having sub-nanosecond resolution. Prompt fluorescence spectra were integrated by iCDD between 1 ns – 100 ns after the laser excitation. Phosphorescence spectra were integrated by iCDD between 0.5 – 1 ms after the laser excitation. The energy values of the lowest singlet and triplet states were

determined from the onset of prompt and phosphorescence spectra at 77 K. The singlet-triplet splitting energy, ΔE_{ST}, was determined by recording the prompt fluorescence and the phosphorescence spectra at 77 K.

Photostability measurements were carried out using a helium-cadmium laser (Kimmon Koha, IK5351R-D) as the continuous excitation source and a fibre-coupled CCD spectrograph (Andor, Model Number: DV420-BV) as the detector. The laser emits at 325 nm with optical power of 4.18 mW (intensity: 440 mW/cm^2). The emitters were dissolved in toluene at the concentration of 10^{-5} M and the solutions were then transferred into NMR tubes (80 µL) and were exposed to the laser beam. The samples emission was collected by the CCD with the integration duration of 30 s.

Electrochemistry Measurements

An electrochemical Analyzer model 620D from CH Instruments was used for Cyclic Voltammetry (CV) analysis. All samples were prepared as acetonitrile (MeCN) solutions and bubbled with MeCN-saturated nitrogen gas for 15 minutes before measurements. A 0.1 M MeCN solution of tetra-*n*-butylammonium hexafluorophosphate [nBu$_4$N][PF$_6$] was used as the electrolyte solution. An Ag/Ag$^+$ electrode was used as the reference electrode while a platinum electrode and a platinum wire were used as the working electrode and counter electrode, respectively. The redox potentials are reported relative to a saturated calomel electrode (SCE) with a ferrocene/ferrocenium (Fc/Fc$^+$) redox couple as the internal standard (0.38 V vs SCE in MeCN).[9]

Synthetic Schemes

The following compounds were synthesised according to literature procedures: 1-(azidomethyl)-3,5-di-*tert*-butylbenzene **S1**,[10] macrocycle **S2**.[11]

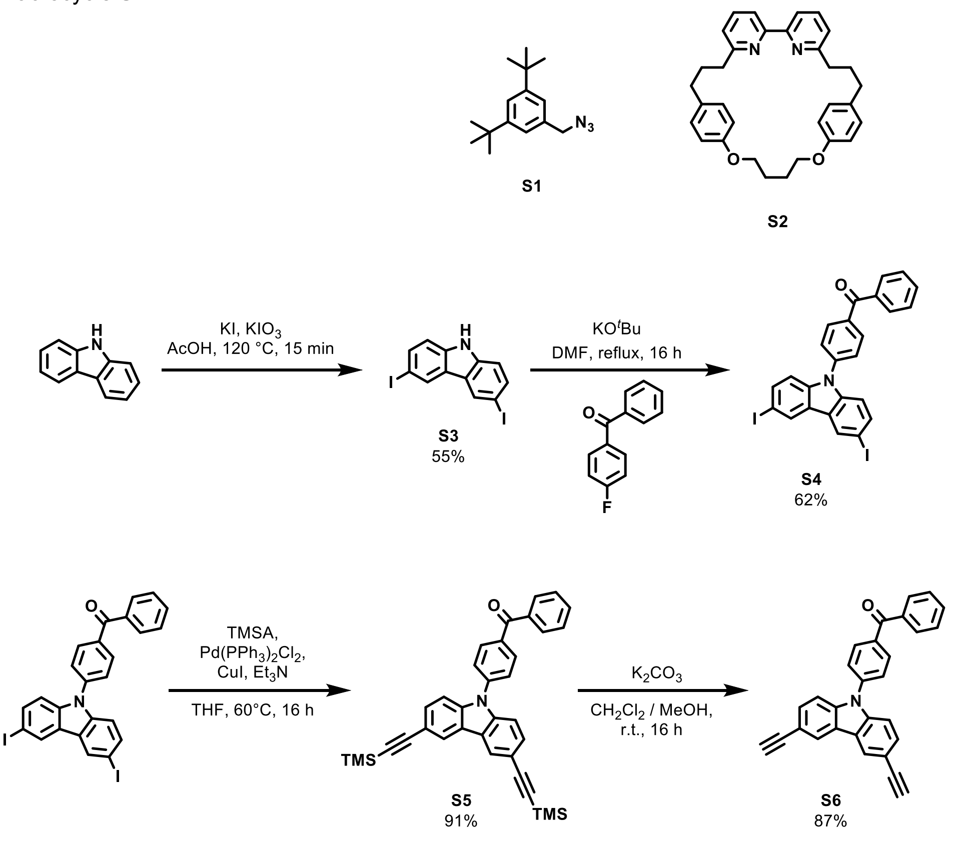

Scheme S1. Synthesis of the building blocks.

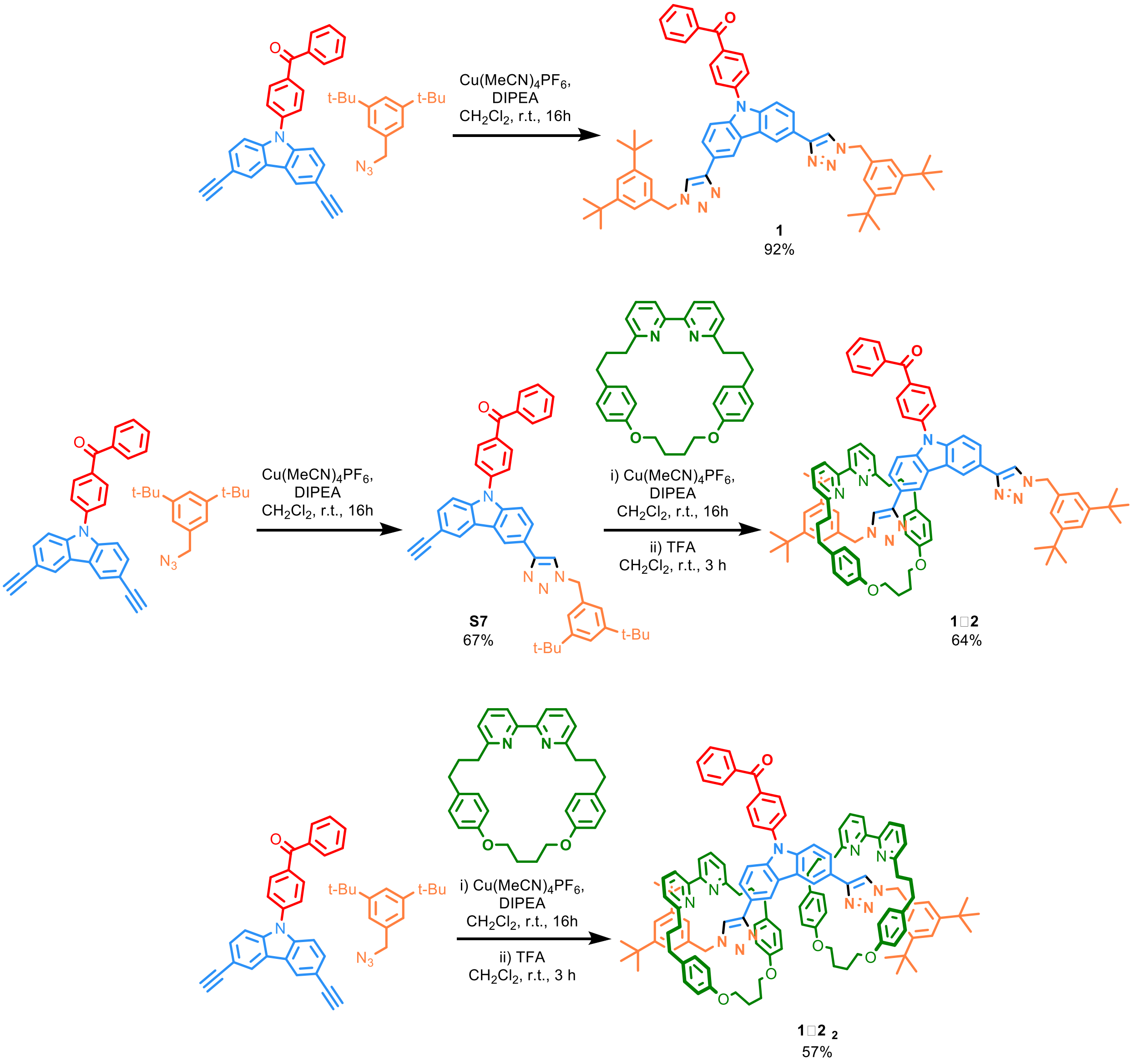

Scheme S2. Synthesis of the TADF emitters.

3,6-Diiodo-9H-carbazole S3

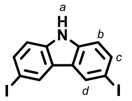

To a solution of 9*H*-carbazole (1.40 g, 8.40 mmol) in AcOH (30 mL) was added KI (1.86 g, 11.2 mmol), and the mixture was heated to 80 °C. KIO_3 (1.21 g, 5.66 mmol) was slowly added, and the mixture was refluxed for 15 min. The reaction mixture was allowed to cool to room temperature, suspended in deionised H_2O (200 mL) and filtered. The crude was washed with 1 M $NaHCO_{3(aq)}$ (50 mL) and MeOH (50 mL). Compound **S3** was obtained as a dark red powder (1.95 g, 55%). ^{1}H NMR (400 MHz, $CDCl_3$) δ_H 8.33 (d, *J* = 1.8, 2H, H_d), 8.11-8.07 (br. s, 1H, H_a), 7.68 (dd, *J* = 8.5, 1.8, 2H, H_c), 7.22 (dd, *J* = 8.5, 0.6, 2H, H_b); ^{13}C NMR (101 MHz, $CDCl_3$) δ_C 138.5, 134.8, 129.4, 124.6, 112.7, 82.5; HRMS (APPI): *m/z* = 418.8656 $[M]^{\cdot}$, calc. 418.8668. Values in accordance with literature.[12]

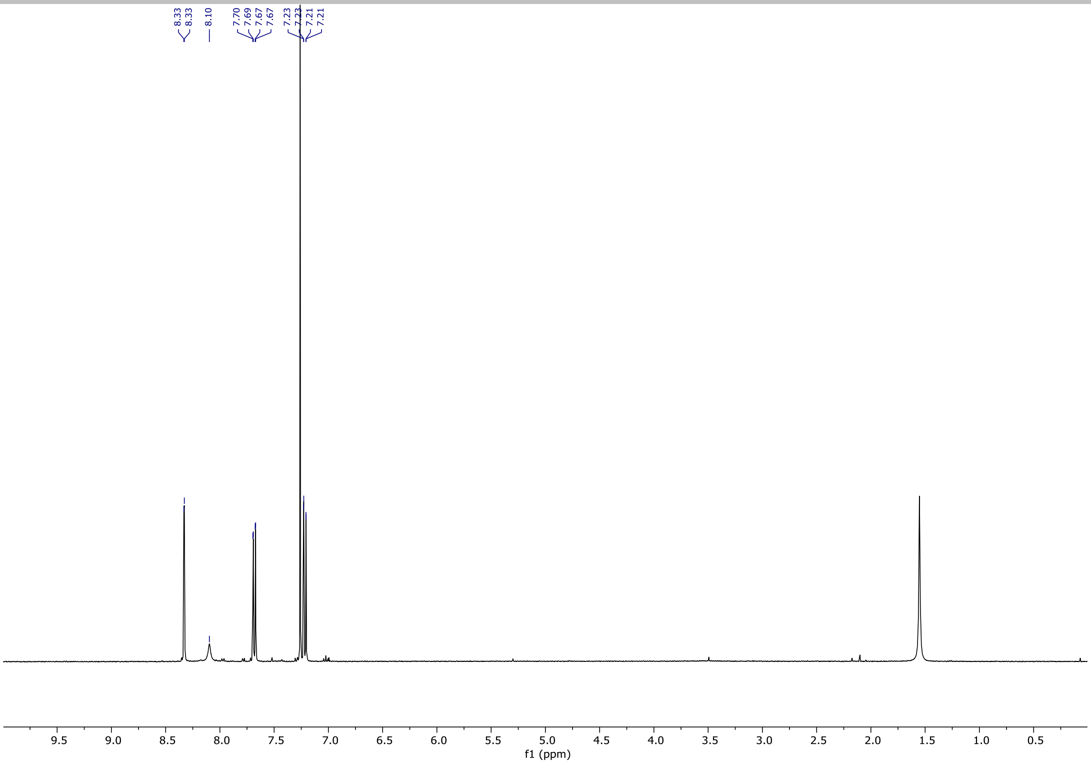

Figure S1. ^{1}H NMR (400 MHz, $CDCl_3$) of **S3**.

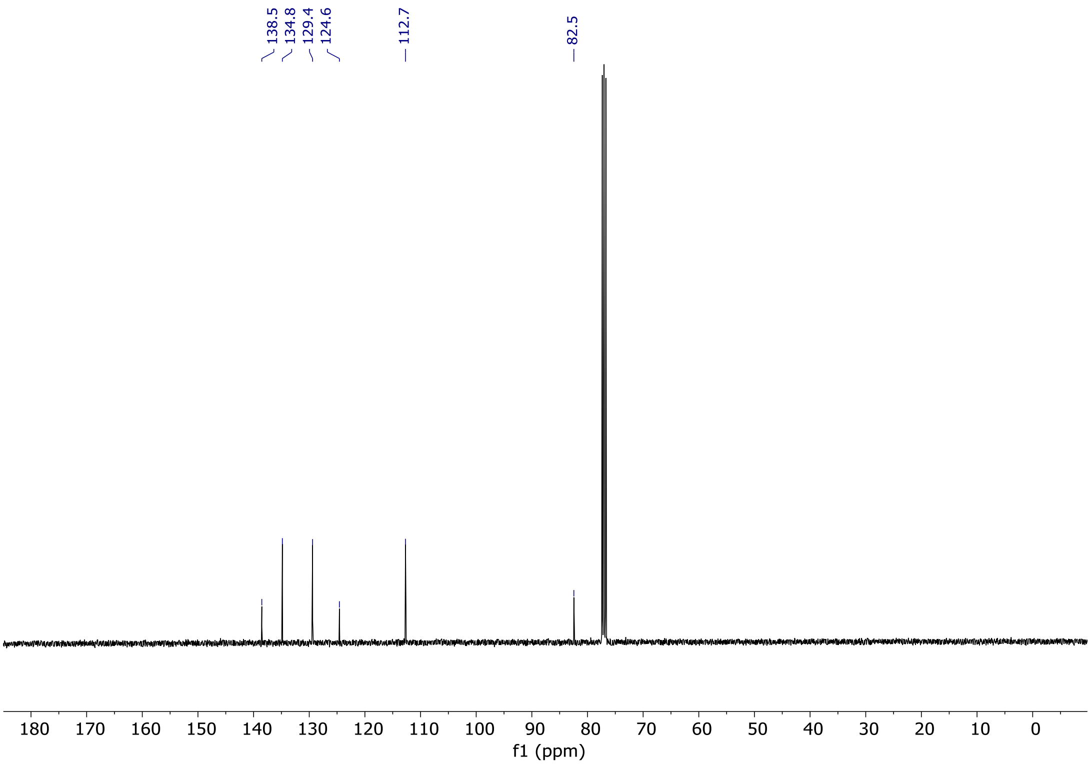

Figure S2. ^{13}C NMR (101 MHz, $CDCl_3$) of **S3**.

4-(3,6-Dibromo-9H-carbazol-9-yl)-phenyl-methanone S4

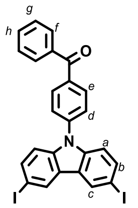

Compound **S3** (588 mg, 1.40 mmol) and KO^{t}Bu (277 mg, 2.47 mmol) were dissolved in anhydrous DMF (40 mL) under a nitrogen atmosphere, and the solution was stirred at 80 °C for 30 min. To this was added *via* syringe a solution of 4-fluorobenzophenone (287 mg, 1.43 mmol) in anhydrous DMF (5 mL) under a nitrogen atmosphere, and the mixture stirred at 130 °C for 16 h. The solution was allowed to cool, poured onto 5 M $HCl_{(aq)}$ (50 mL) and extracted with Et_2O (3 x 50 mL). The combined organic phases were washed with brine (3 x 100 mL) and then 5% w/v $LiCl_{(aq)}$ (2 x 100 mL), dried with $MgSO_4$, filtered and concentrated *in vacuo*. Purification *via* column chromatography (petrol : Et_2O = 9 : 1, isocratic) afforded **S4** as an off-white solid (523 mg, 62%). ^{1}H NMR (400 MHz, $CDCl_3$) δ_H 8.41 (dd, *J* = 1.8, 0.5, 2H, H_c), 8.08 (d, *J* = 8.7, 2H, H_e), 7.90 (dd, *J* = 8.4, 1.4, 2H, H_f), 7.71 (dd, *J* = 8.6, 1.7, 2H, H_b), 7.67 – 7.63 (m, 3H, H_d, H_h), 7.55 (app. t, *J* = 7.5, 2H, H_g), 7.26 (dd, *J* = 8.6, 0.5,, 2H, H_a); ^{13}C NMR (100 MHz, $CDCl_3$) δ_C 195.4, 140.5, 139.6, 137.2, 136.8, 135.2, 132.8, 132.0, 130.0, 129.5, 128.5, 126.3, 124.8, 111.9, 83.5; HRMS (APPI): *m/z* = 598.9228 [M]$^{\bullet}$ (100), calc. 598.9243.

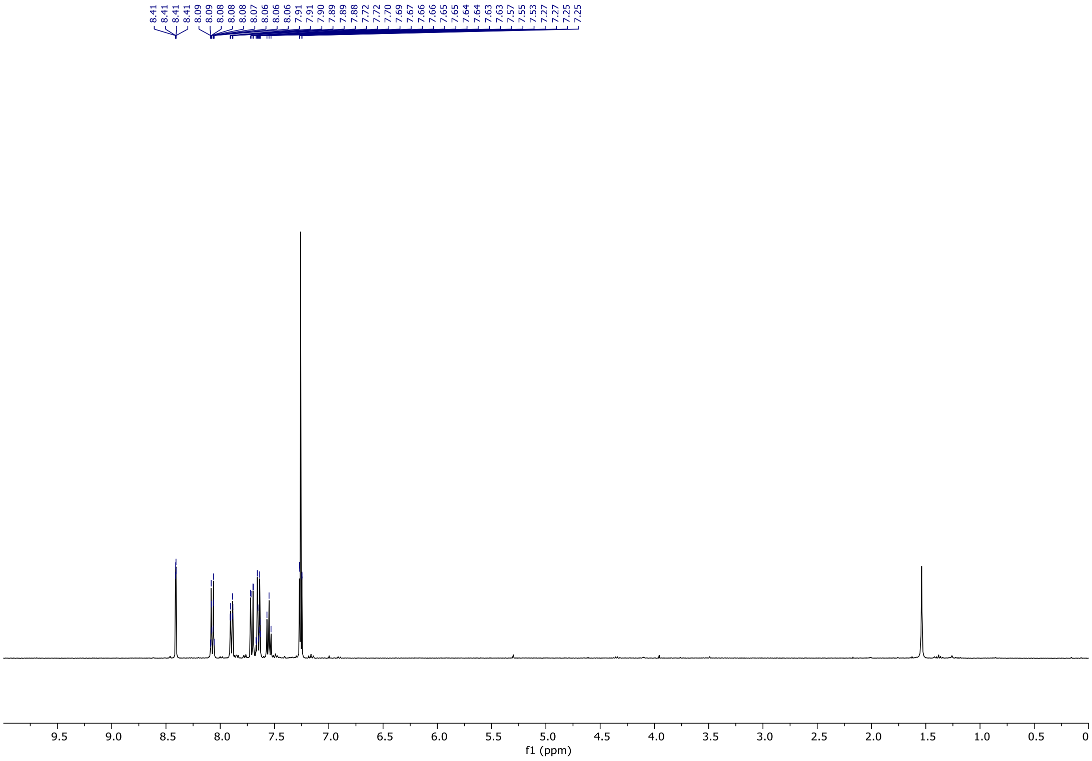

Figure S3. ^{1}H NMR (400 MHz, $CDCl_3$) of **S4**.

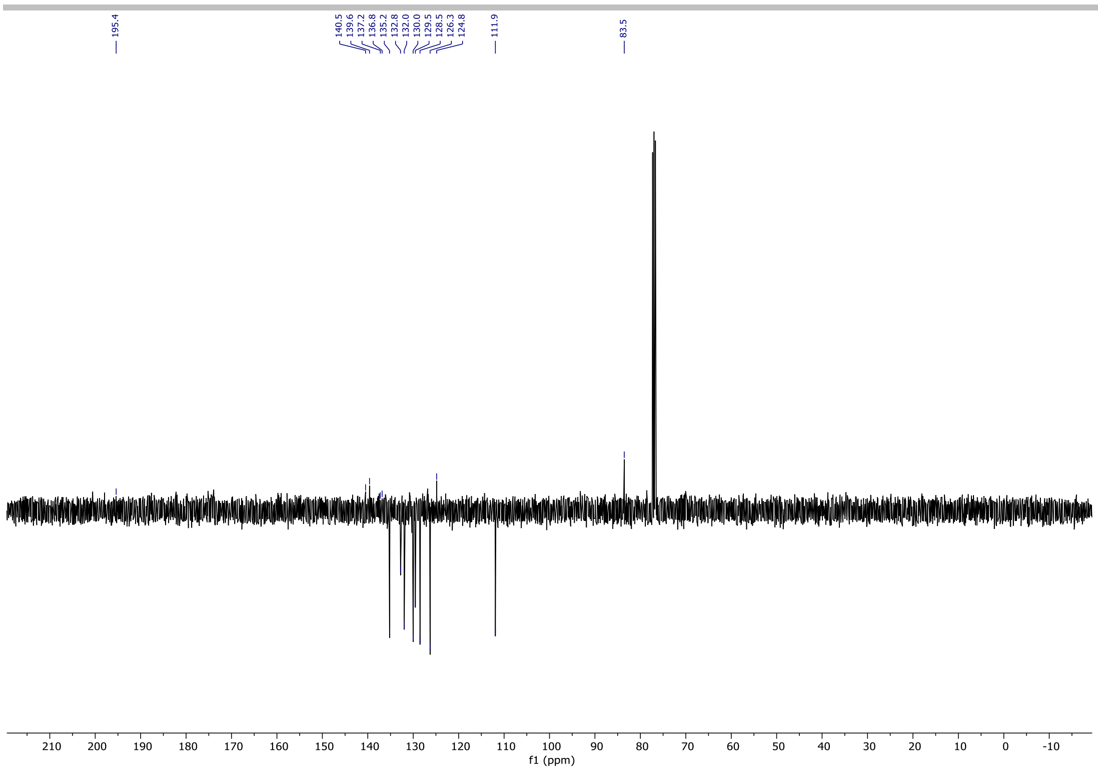

Figure S4. JMOD NMR (101 MHz, $CDCl_3$) of **S4**.

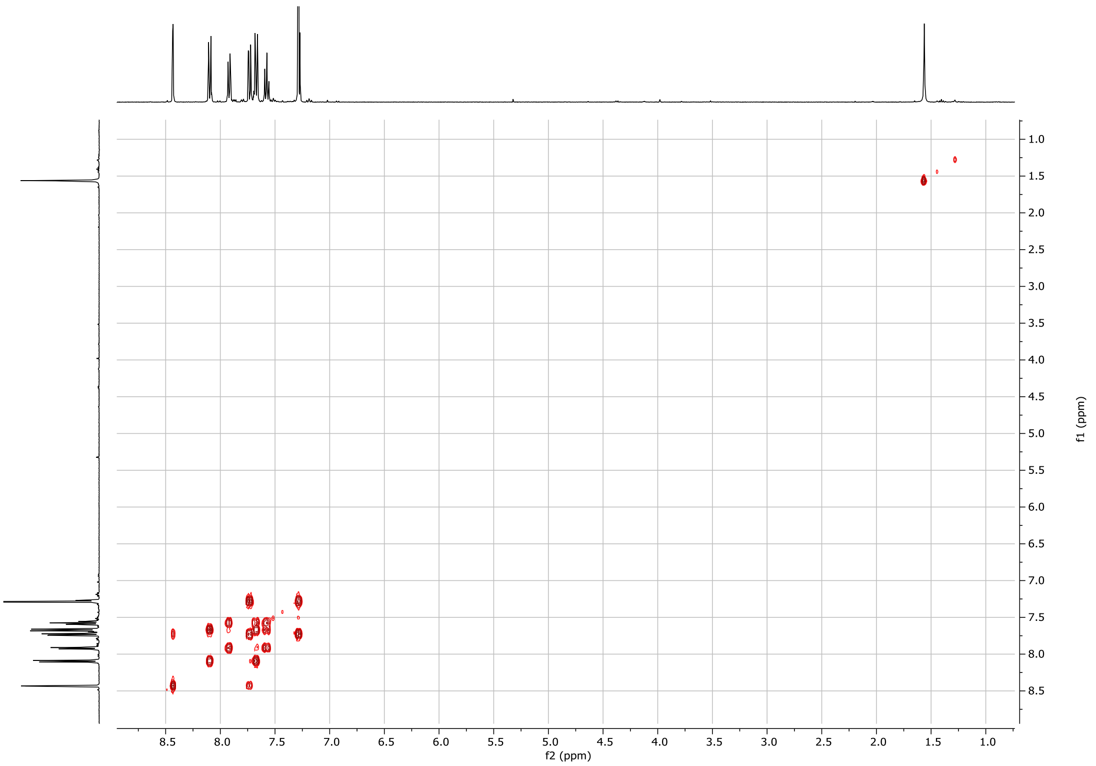

Figure S5. COSY NMR (400 MHz, $CDCl_3$) of **S4**.

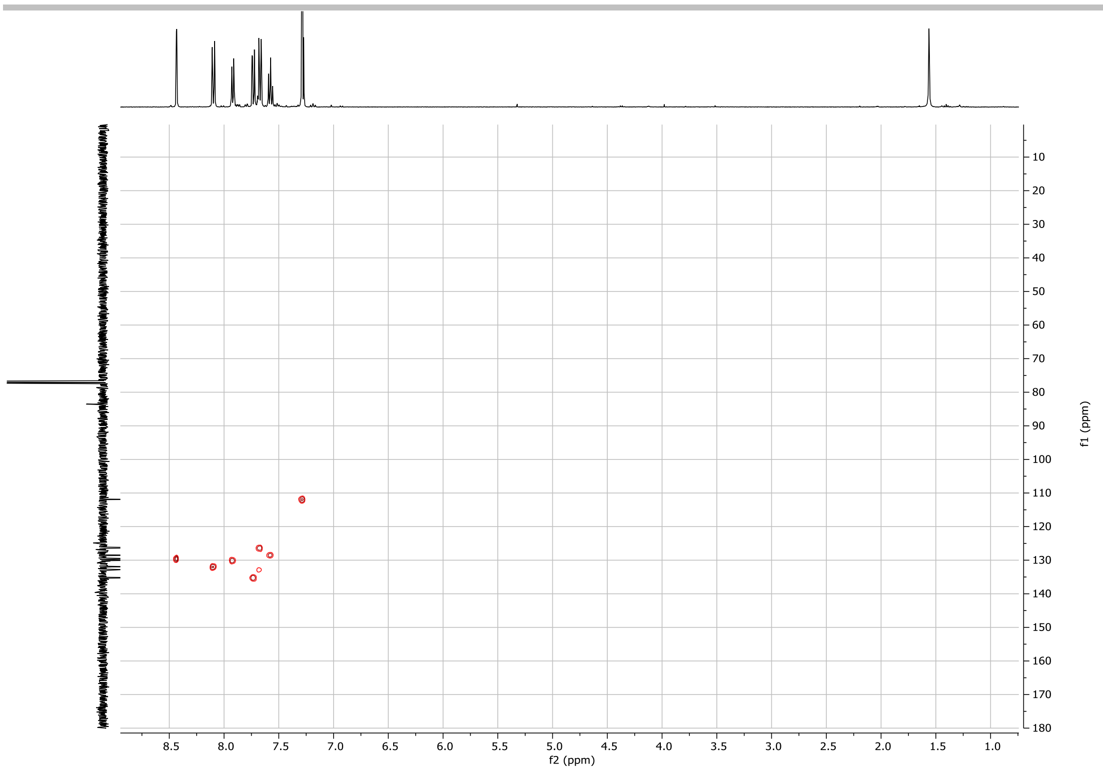

Figure S6. HSQC (101 MHz, $CDCl_3$) of **S4**.

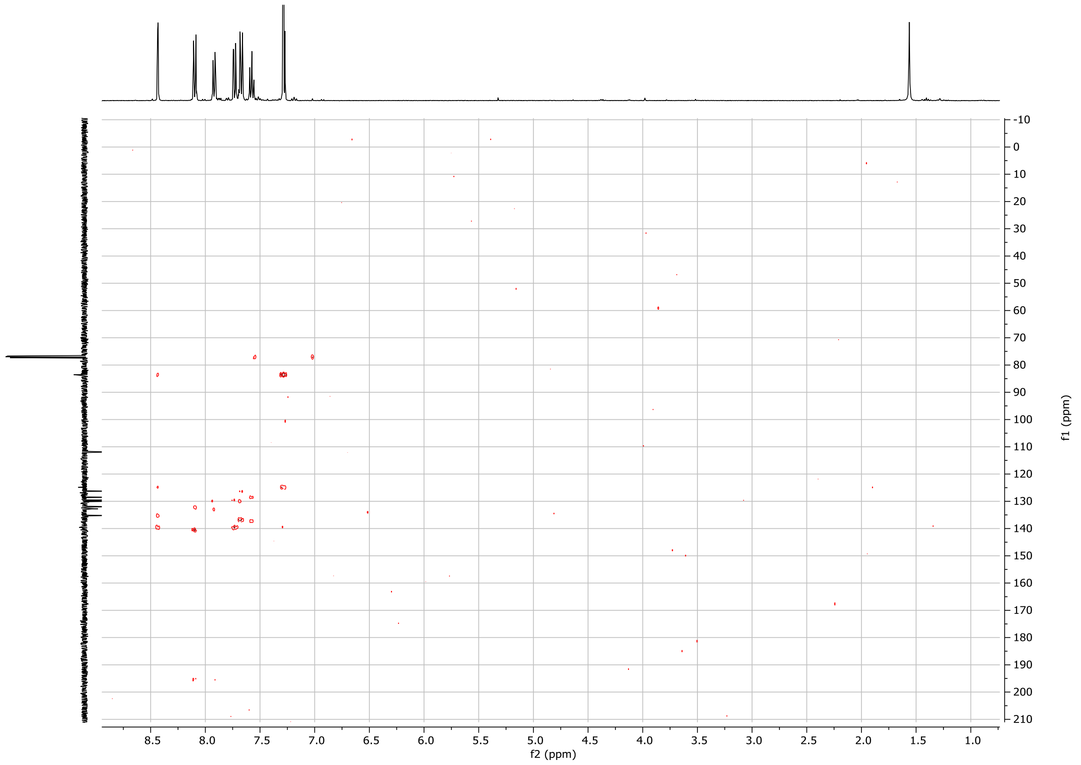

Figure S7. HMBC (101 MHz, $CDCl_3$) of **S4**.

(4-(3,6-bis(3,3-dimethylbut-1-yn-1-yl)-9H-carbazol-9-yl)phenyl)(phenyl)methanone S5

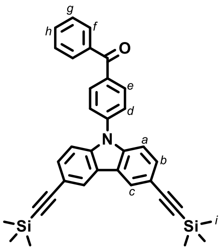

A suspension of **S4** (511 mg, 0.854 mmol), $Pd(PPh_3)_2Cl_2$ (27.6 mg, 39.3 μmol) and CuI (36.6 mg, 0.192 mmol) in Et_3N (12 mL) in a CEM vial was degassed for 30 min. Anhydrous THF (11 mL) and trimethylsilylacetylene (1.2 mL, 8.54 mmol) were added *via* syringe, and the suspension was stirred at 60 °C for 16 h under a nitrogen atmosphere. The suspension was allowed to cool down and the solvent removed *in vacuo*. Purification *via* column chromatography (petrol, with a 0-100% CH_2Cl_2 gradient) yielded **S5** as an off-white foam (419 mg, 91%). ^{1}H NMR (400 MHz, $CDCl_3$) δ_H 8.25 (dd, *J* = 1.7, 0.7, 2H, H_c), 8.08 (app. d, *J* = 8.5, 2H, H_e), 7.95-7.85 (m, 2H, H_f), 7.67 (app. d, *J* = 8.5, 2H, H_d), 7.66-7.62 (m, 1H, H_h), 7.60-7.50 (m, 4H, H_b, H_g), 7.39 (dd, *J* = 8.6, 0.7, 2H, H_a), 0.30 (s, 18H, H_i); ^{13}C NMR (100 MHz, $CDCl_3$) δ_C 195.3, 140.5, 140.2, 137.1, 136.6, 132.7, 131.8, 130.4, 129.9, 128.4, 126.2, 124.6, 123.1, 115.5, 109.7, 105.7, 92.7, 0.0; HRMS (APPI): *m/z* = 539.2083 $[M]^{\bullet}$ (100), calc. 539.2101.

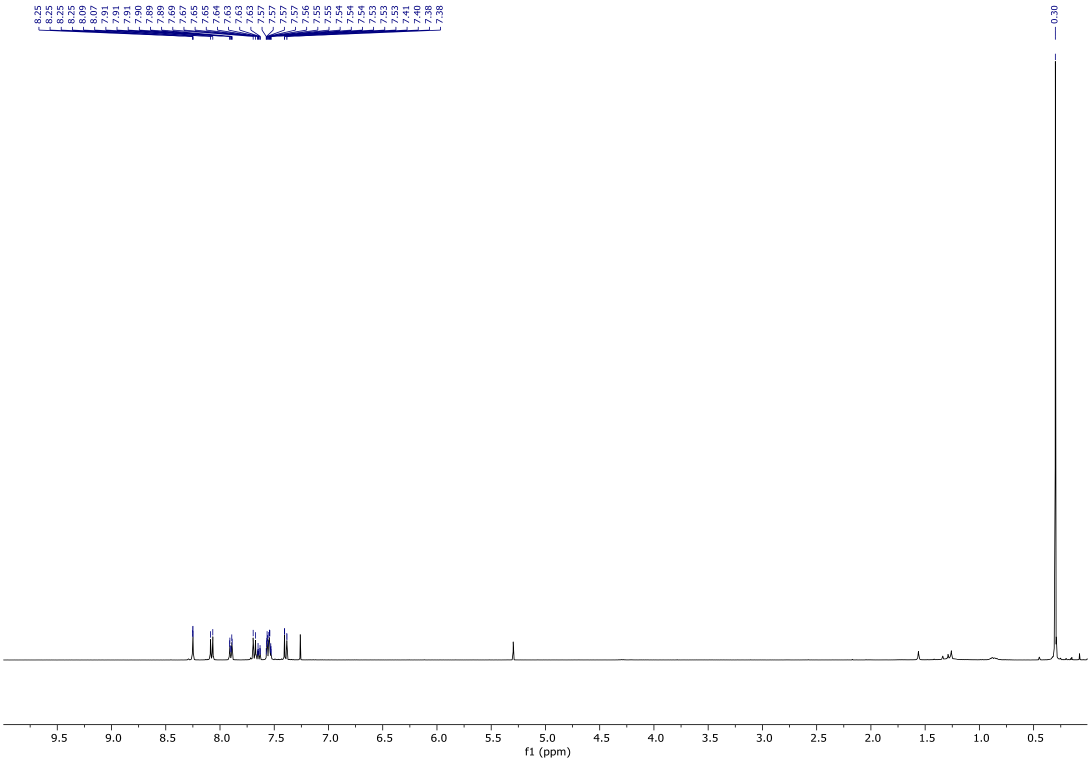

Figure S8. ^{1}H NMR (400 MHz, $CDCl_3$) of **S5**.

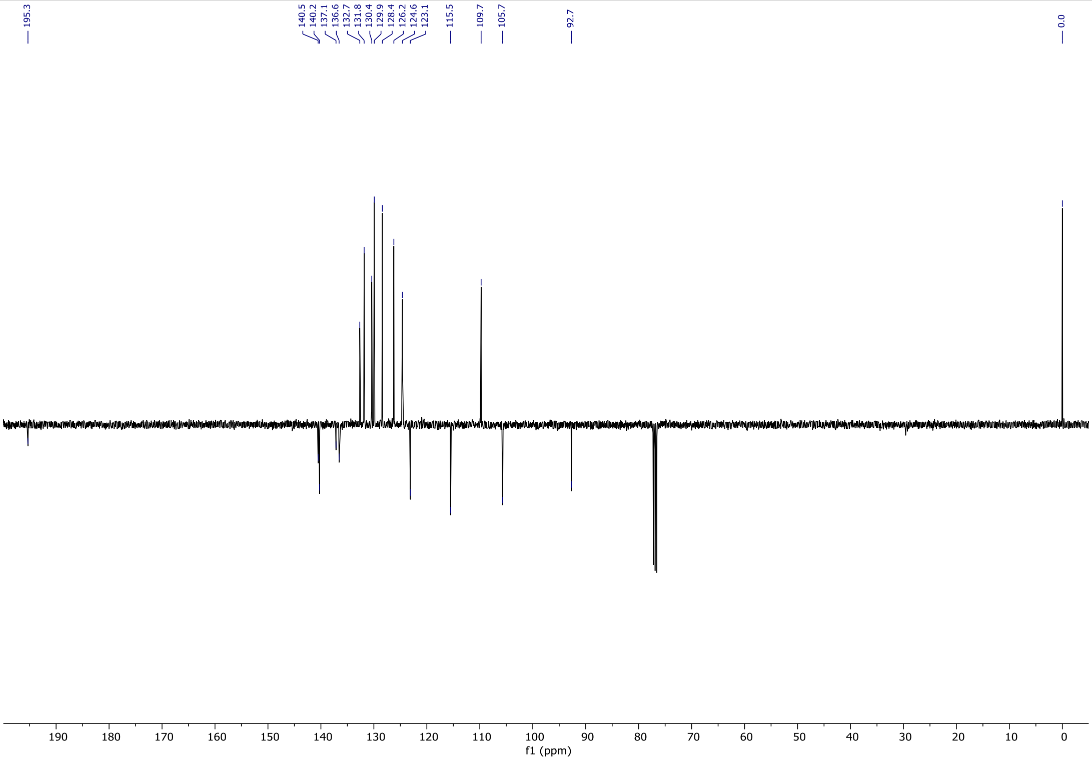

Figure S9. JMOD NMR (101 MHz, $CDCl_3$) of **S5**.

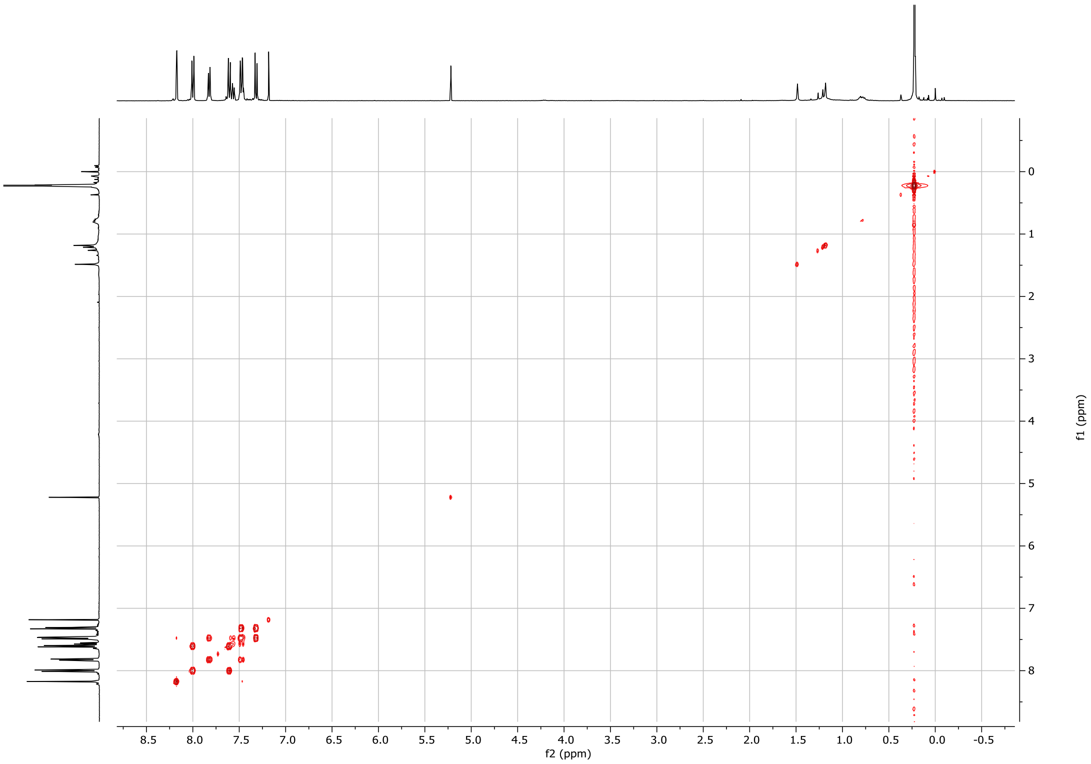

Figure S10. COSY NMR (400 MHz, $CDCl_3$) of **S5**.

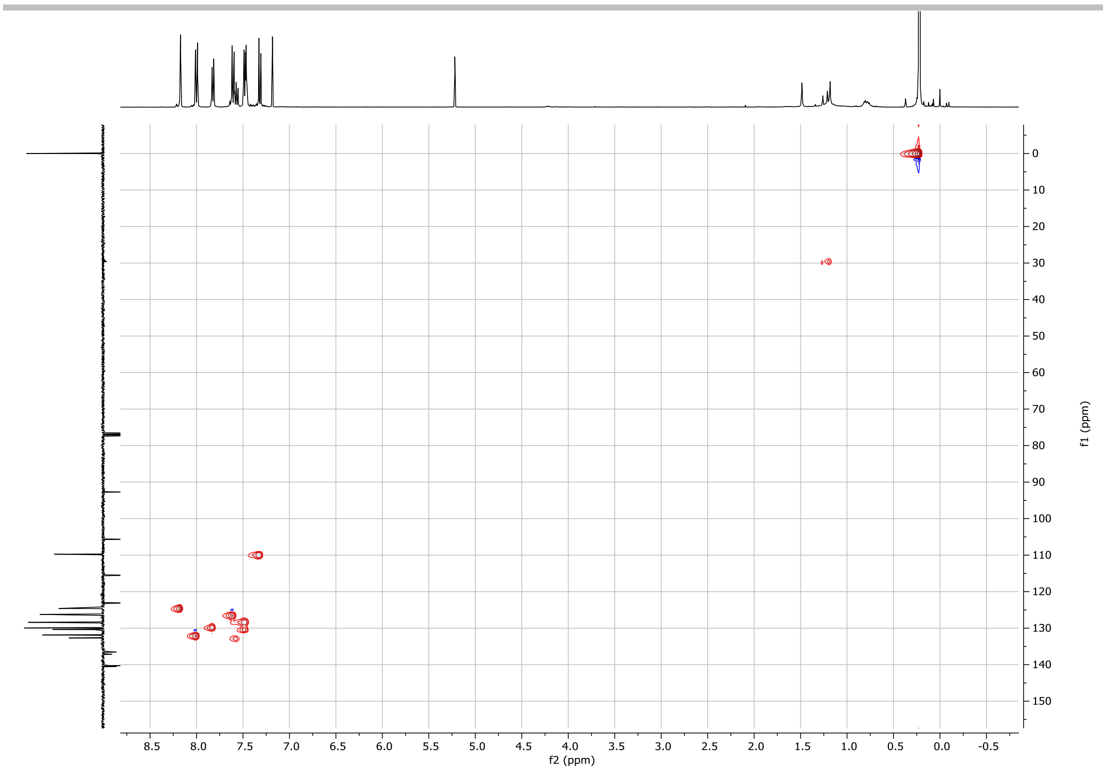

Figure S11. HSQC NMR (101 MHz, $CDCl_3$) of **S5**.

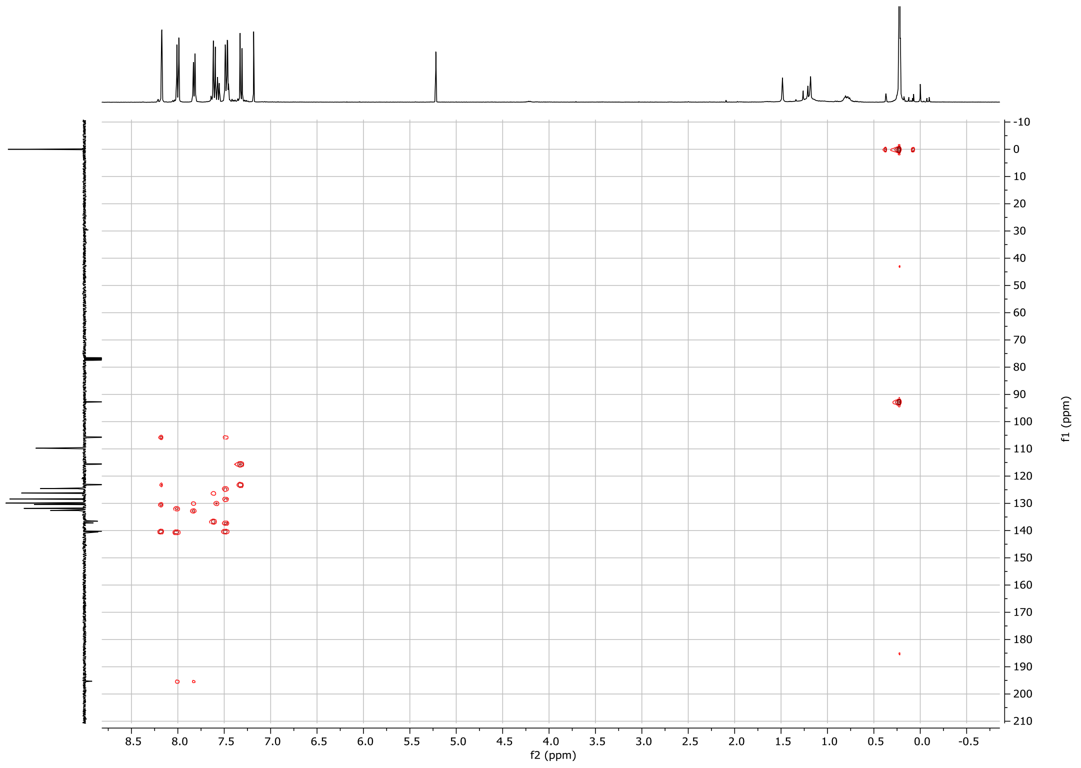

Figure S12. HMBC NMR (101 MHz, $CDCl_3$) of **S5**.

(4-(3,6-diethynyl-9H-carbazol-9-yl)phenyl)(phenyl)methanone S6

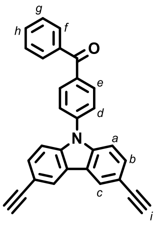

To a solution of **S5** (253 mg, 0.47 mmol) in CH_2Cl_2 (10 mL) was added MeOH (15 mL) and K_2CO_3 (4.39 g, 31.8 mmol), and the mixture was stirred vigorously at r.t. for 16 h. The mixture was then poured onto H_2O (50 mL) and the organic material extracted with CH_2Cl_2 (3 x 50 mL), dried with $MgSO_4$, filtered and concentrated *in vacuo*. Purification *via* column chromatography (petrol, with a 0-100% Et_2O gradient) afforded **S6** as a dark orange foam (161 mg, 87%). ^{1}H NMR (400 MHz, $CDCl_3$) δ_H 8.28 (d, *J* = 0.9, 2H, H_c), 8.08 (app. d, *J* = 8.5, 2H, H_e), 7.94-7.86 (m, 2H, H_f), 7.69 (app. d, *J* = 8.5, 2H, H_d), 7.68-7.61 (m, 1H, H_h), 7.60-7.51 (m, 4H, H_b, H_g), 7.42 (dd, *J* = 8.5, 0.7, 2H, H_a), 3.11 (s, 2H, H_i); ^{13}C NMR (101 MHz, $CDCl_3$) δ_C 195.4, 140.6, 140.5, 137.2, 136.8, 132.8, 132.0, 130.7, 130.1, 128.5, 126.4, 124.8, 123.2, 114.6, 110.1, 84.3, 76.1; HRMS (APPI): *m/z* = 395.1302 $[M]^{\bullet}$, calc. 395.1310.

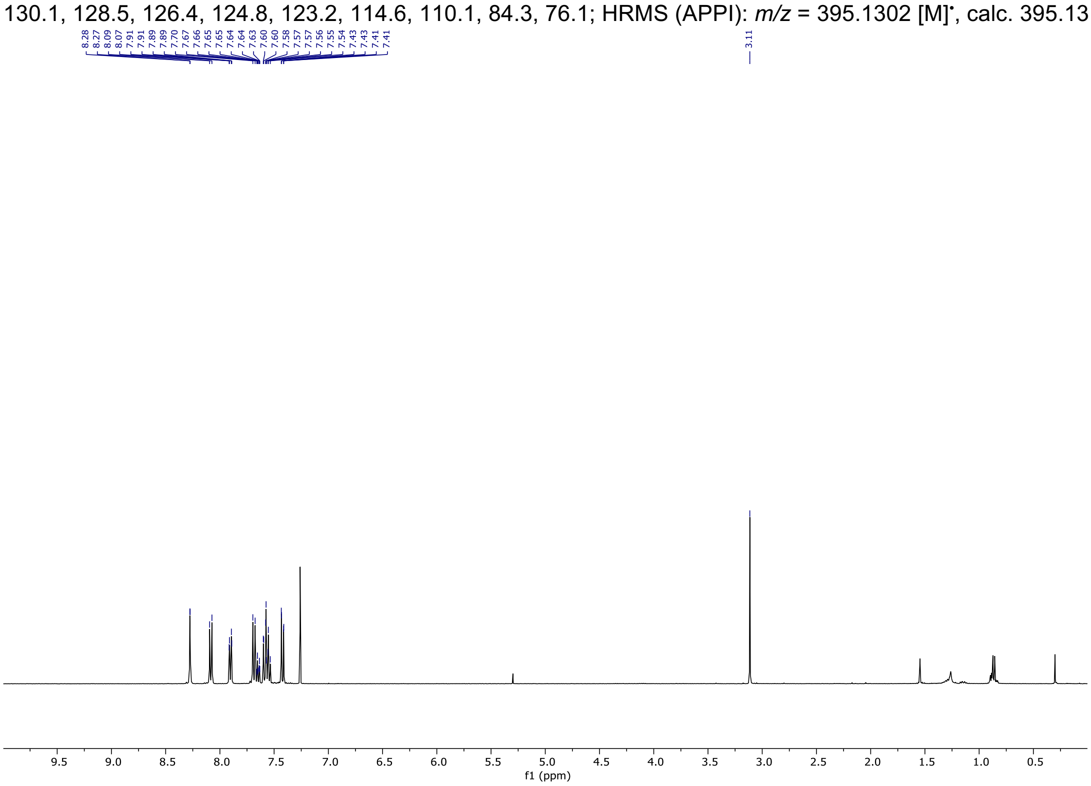

Figure S13. ^{1}H NMR (400 MHz, $CDCl_3$) of **S6**.

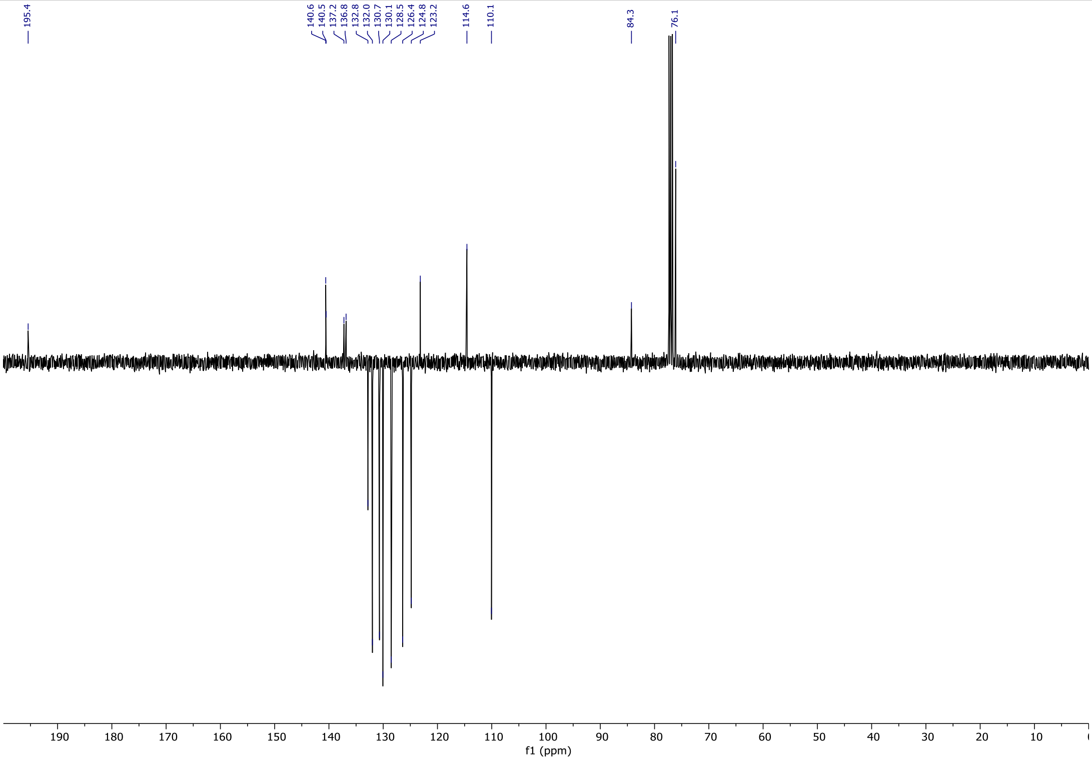

Figure S14. JMOD NMR (101 MHz, $CDCl_3$) of **S6**.

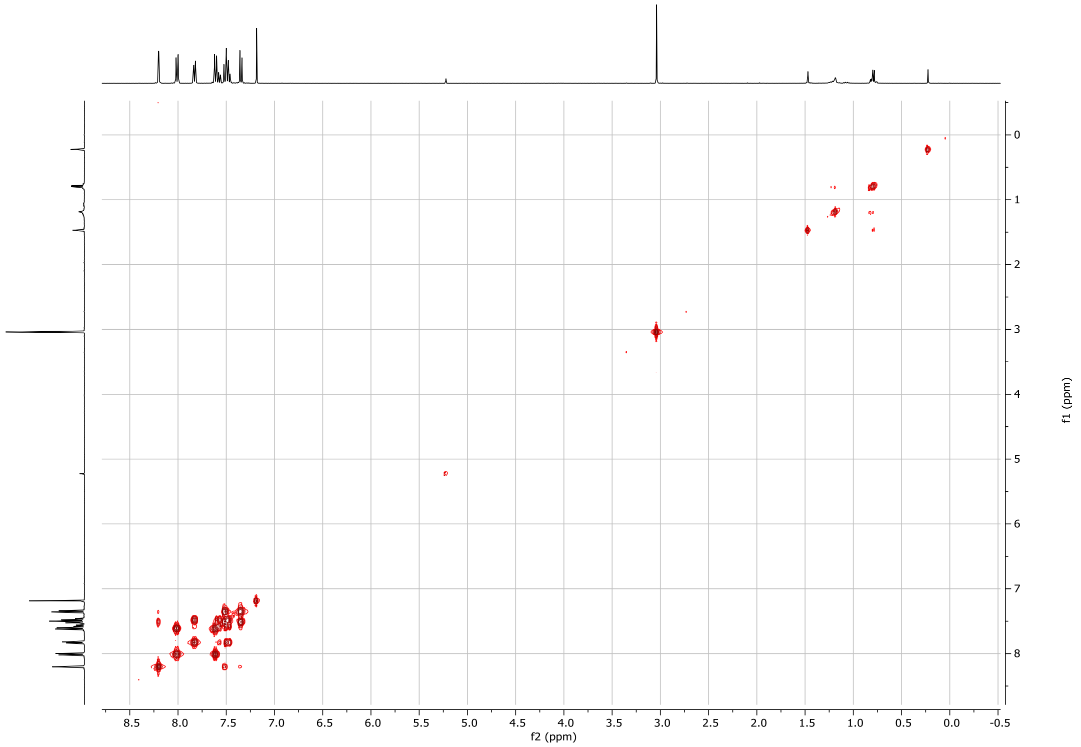

Figure S15. COSY NMR (400 MHz, $CDCl_3$) of **S6**.

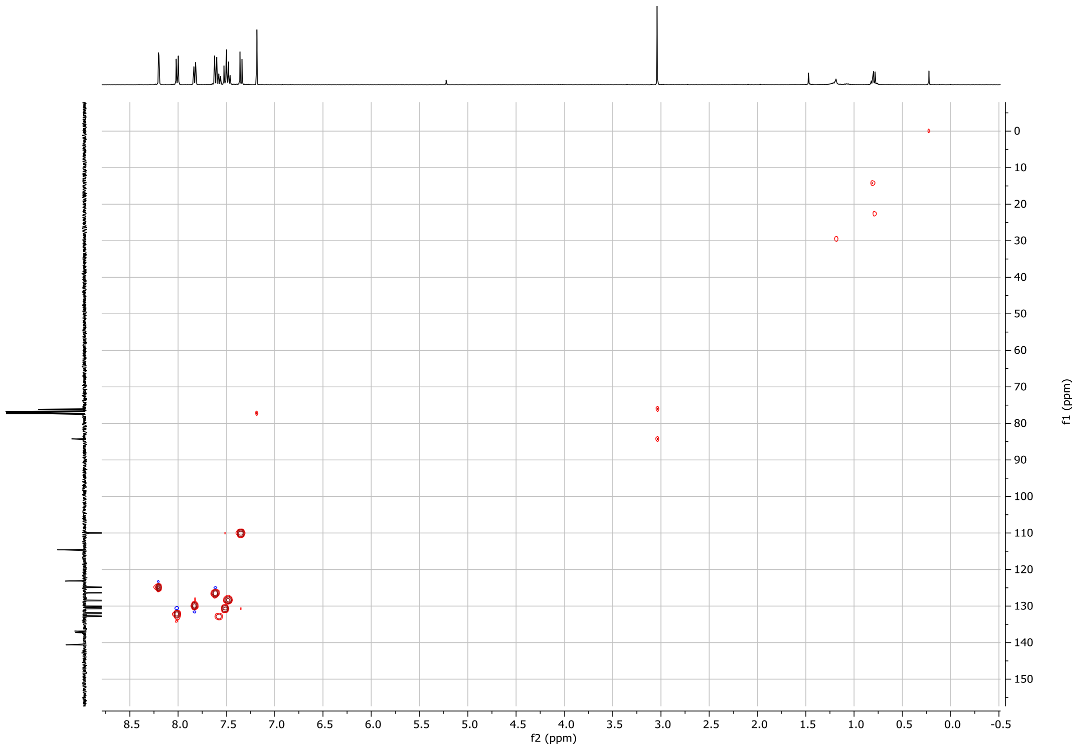

Figure S16. HSQC NMR (101 MHz, $CDCl_3$) of **S6**.

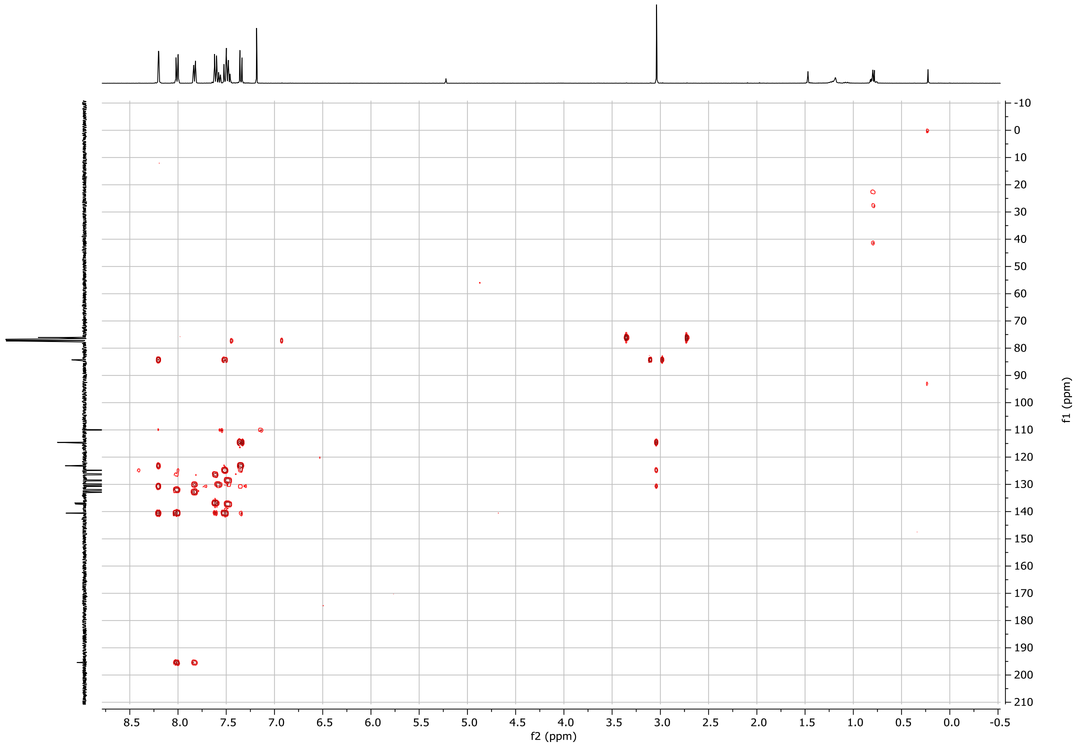

Figure S17. HMBC NMR (101 MHz, $CDCl_3$) of **S6**.

(4-(3-(1-(3,5-di-tert-butylbenzyl)-1H-1,2,3-triazol-4-yl)-6-ethynyl-9H-carbazol-9-yl)phenyl)(phenyl)methanone S7

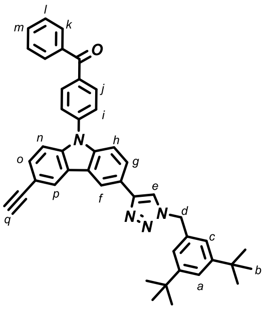

To a solution of **S6** (150 mg, 0.379 mmol), **S1** (44.3 mg, 0.181 mmol) and [Cu(NCCN)$_4$]PF$_6$ (66.4 mg, 0.178 mmol) in dry CH_2Cl_2 (5 mL) under a nitrogen atmosphere was added DIPEA (240 µL, 1.43 mmol) and the solution was stirred at r.t. for 16 h. The solution was poured onto an EDTA-NH_3 solution (20 mL) and the organic material extracted with CH_2Cl_2 (3 x 20 mL), dried with $MgSO_4$, filtered and concentrated *in vacuo*. Purification *via* column chromatography (petrol, with a 0-100% CH_2Cl_2 gradient, then CH_2Cl_2, with a 0-10% ethyl acetate gradient) afforded **S7** as a yellow foam (78.5 mg, 67%). ^{1}H NMR (400 MHz, $CDCl_3$) δ_H 8.58 (dd, *J* = 1.7, 0.6, 1H, H_f), 8.32 (dd, *J* = 1.7, 0.7, 1H, H_p), 8.08 (app. d, *J* = 8.5, 2H, H_j), 7.94-7.88 (m, 3H, H_g, H_k), 7.76 (s, 1H, H_e), 7.71 (app. d, *J* = 8.5, 2H, H_i), 7.68-7.62 (m, 1H, H_m), 7.58-7.49 (m, 4H, H_h, H_l, H_o), 7.45 (t, *J* = 1.8, 1H, H_a), 7.43 (dd, *J* = 8.6, 0.7, 1H, H_n), 7.21 (d, *J* = 1.8, 2H, H_c), 5.60 (s, 2H, H_d), 3.10 (s, 1H, H_q), 1.33 (s, 18H, H_b); ^{13}C NMR (101 MHz, $CDCl_3$) δ_C 195.5, 151.9, 148.5, 140.9, 140.5, 140.5, 137.3, 136.6, 133.9, 132.8, 132.0, 130.4, 130.1, 128.5, 126.3, 124.9, 124.8, 122.9, 122.5, 119.0, 117.9, 114.2, 110.3, 110.0, 84.4, 75.9, 55.0, 34.9, 31.4; HRMS (ESI+, MeCN): *m/z* = 641.3285 [M+H]$^+$, calc. 641.3275.

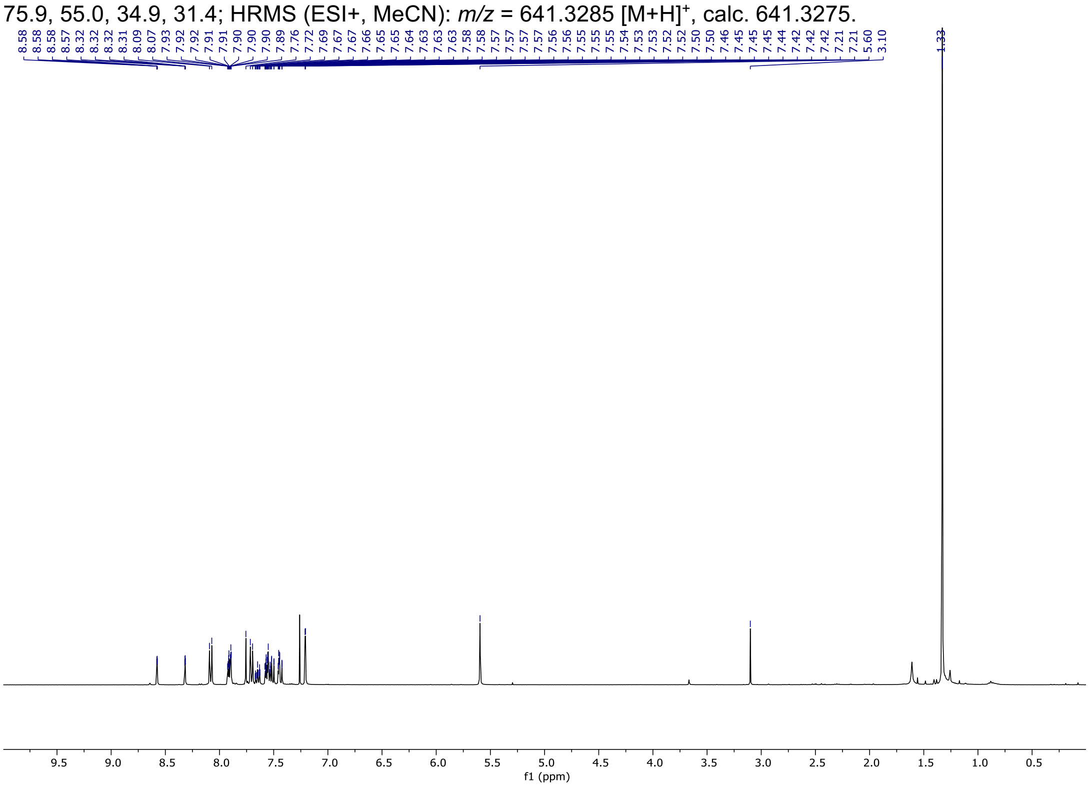

Figure S18. ^{1}H NMR (400 MHz, $CDCl_3$) of **S7**.

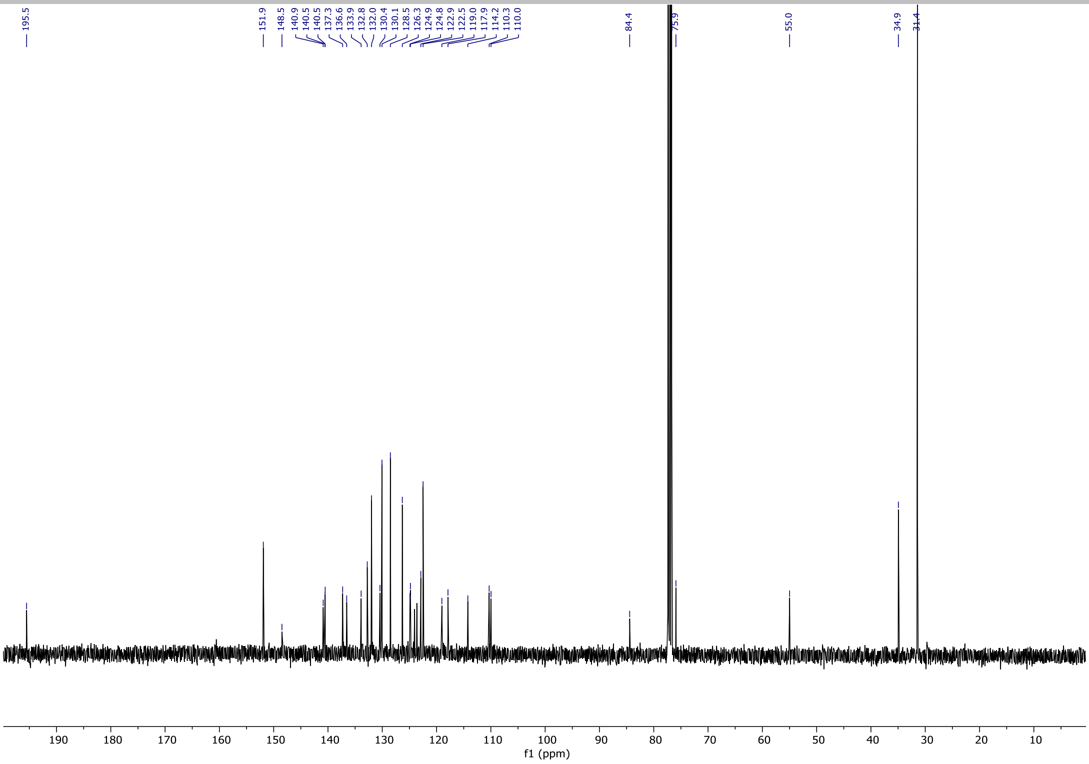

Figure S19. JMOD NMR (101 MHz, $CDCl_3$) of **S7**.

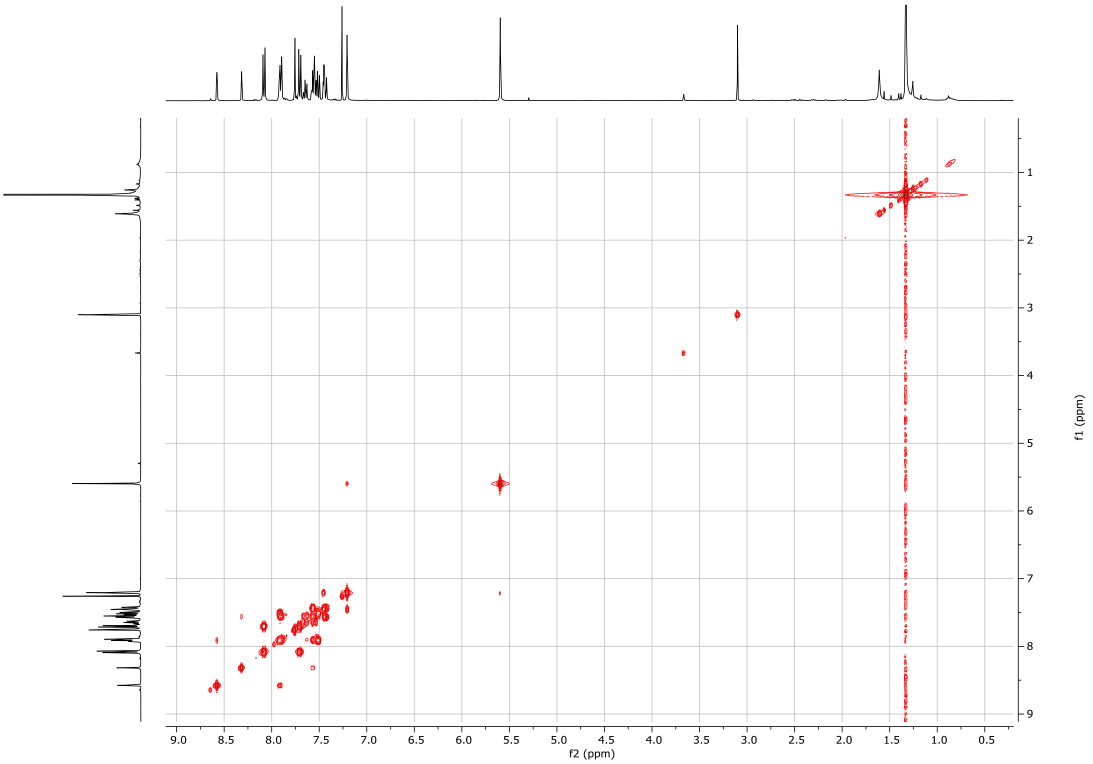

Figure S20. COSY NMR (400 MHz, $CDCl_3$) of **S7**.

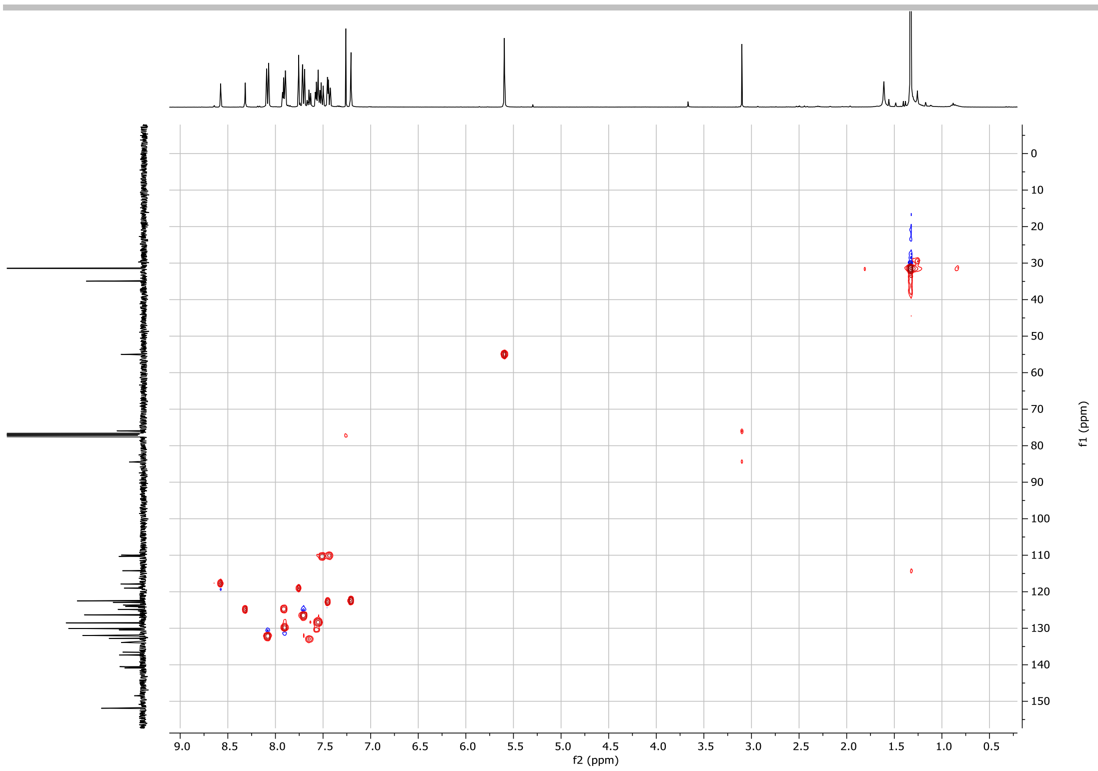

Figure S21. HSQC NMR (101 MHz, $CDCl_3$) of **S7**.

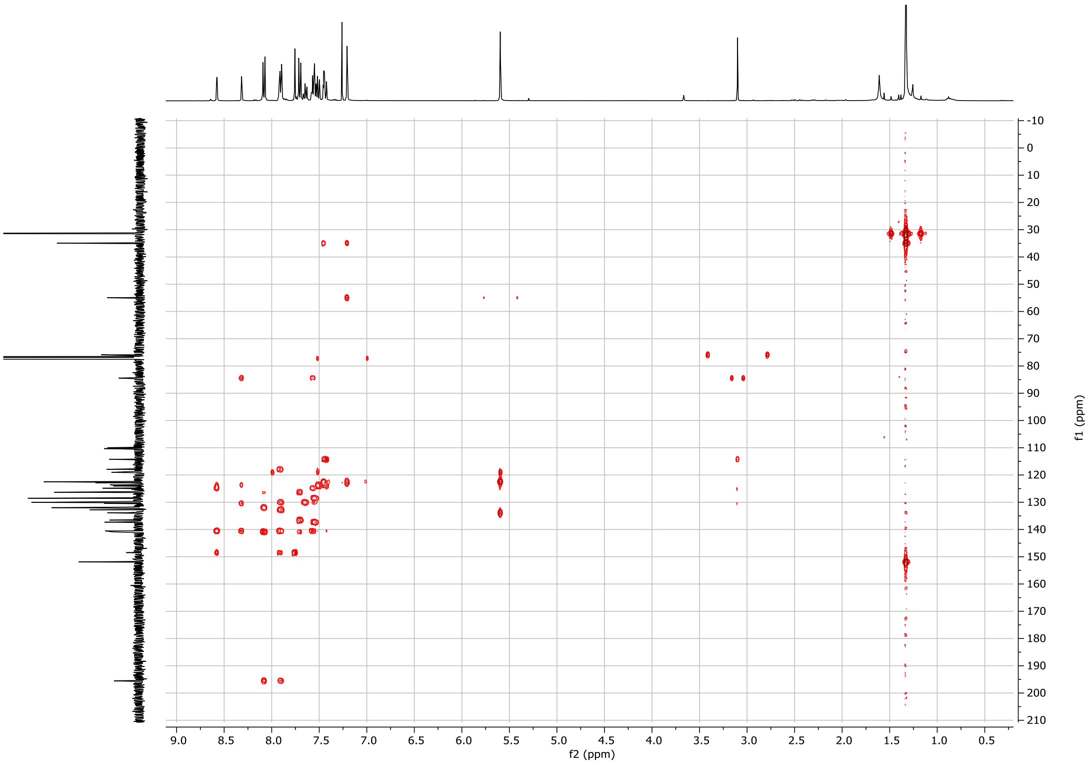

Figure S22. HMBC NMR (101 MHz, $CDCl_3$) of **S7**.

Synthesis of Axle 1

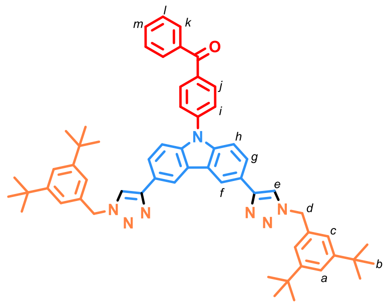

To a solution of **S6** (150 mg, 0.379 mmol), **S1** (44.3 mg, 0.181 mmol) and $[Cu(NCMe)_4]PF_6$ (66.4 mg, 0.178 mmol) in dry CH_2Cl_2 (5 mL) under a nitrogen atmosphere was added DIPEA (240 µL, 1.43 mmol) and the solution was stirred at r.t. for 16 h. The solution was poured onto an EDTA-NH_3 solution (20 mL) and the organic material extracted with CH_2Cl_2 (3 x 20 mL), dried with $MgSO_4$, filtered and concentrated *in vacuo*. Purification *via* column chromatography (petrol, with a 0-100% CH_2Cl_2 gradient, then CH_2Cl_2, with a 0-10% ethyl acetate gradient) afforded **1** as a yellow foam (43.2 mg, 27%). ^{1}H NMR (400 MHz, $CDCl_3$) δ_H 8.61 (dd, *J* = 1.7, 0.6, 2H, H_f), 8.08 (app. d, *J* = 8.5, 2H, H_j), 7.94-7.89 (m, 4H, H_g, H_k), 7.74 (s, 2H, H_e), 7.73 (app. d, *J* = 8.5, 2H, H_i), 7.67-7.62 (m, 1H, H_m), 7.58-7.51 (m, 4H, H_h, H_l), 7.46 (t, *J* = 1.8, 2H, H_a), 7.21 (d, *J* = 1.8 Hz, 4H, H_c), 5.60 (s, 4H, H_d), 1.33 (s, 36H, H_b); ^{13}C NMR (101 MHz, $CDCl_3$) δ_C 195.5, 151.9, 148.6, 141.3, 140.5, 137.4, 136.3, 133.9, 132.7, 131.9, 130.0, 128.5, 126.2, 124.5, 124.2, 123.8, 122.9, 122.5, 118.9, 117.9, 110.3, 55.0, 34.9, 31.4; HRMS (ESI+, MeCN): *m/z* = 885.5154 $[M+H]^+$, calc. 886.5167.

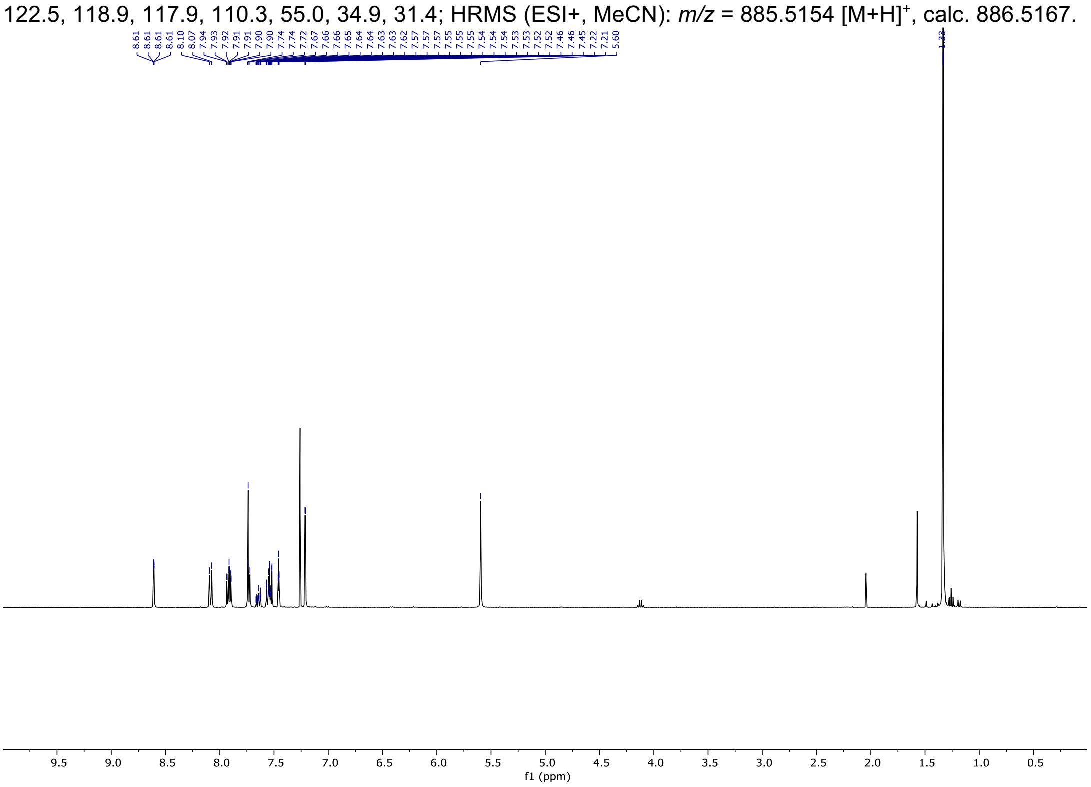

Figure S23. ^{1}H NMR (400 MHz, $CDCl_3$) of **1**.

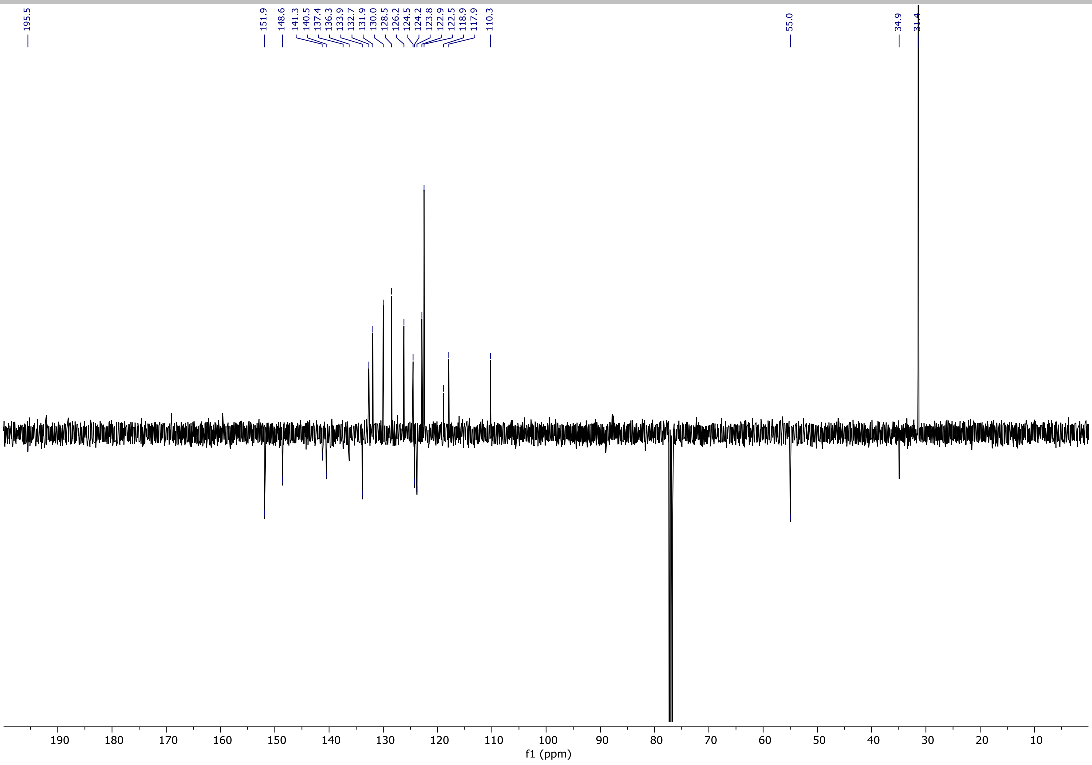

Figure S24. JMOD NMR (101 MHz, $CDCl_3$) of **1**.

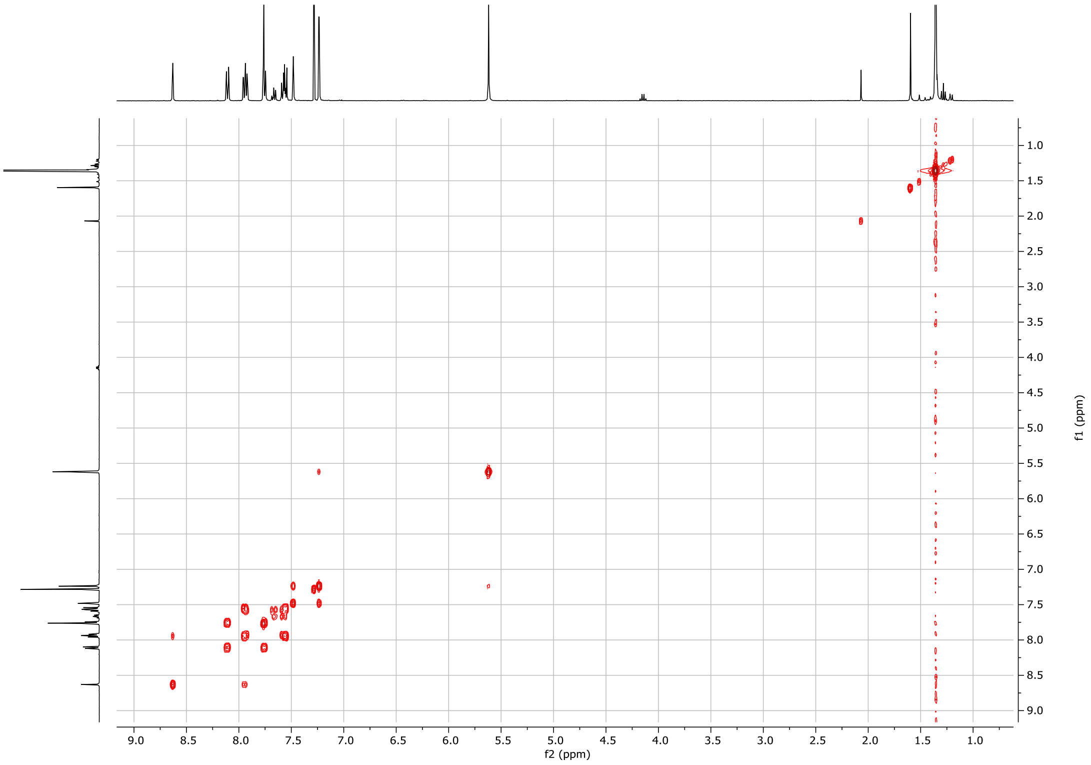

Figure S25. COSY NMR (400 MHz, $CDCl_3$) of **1**.

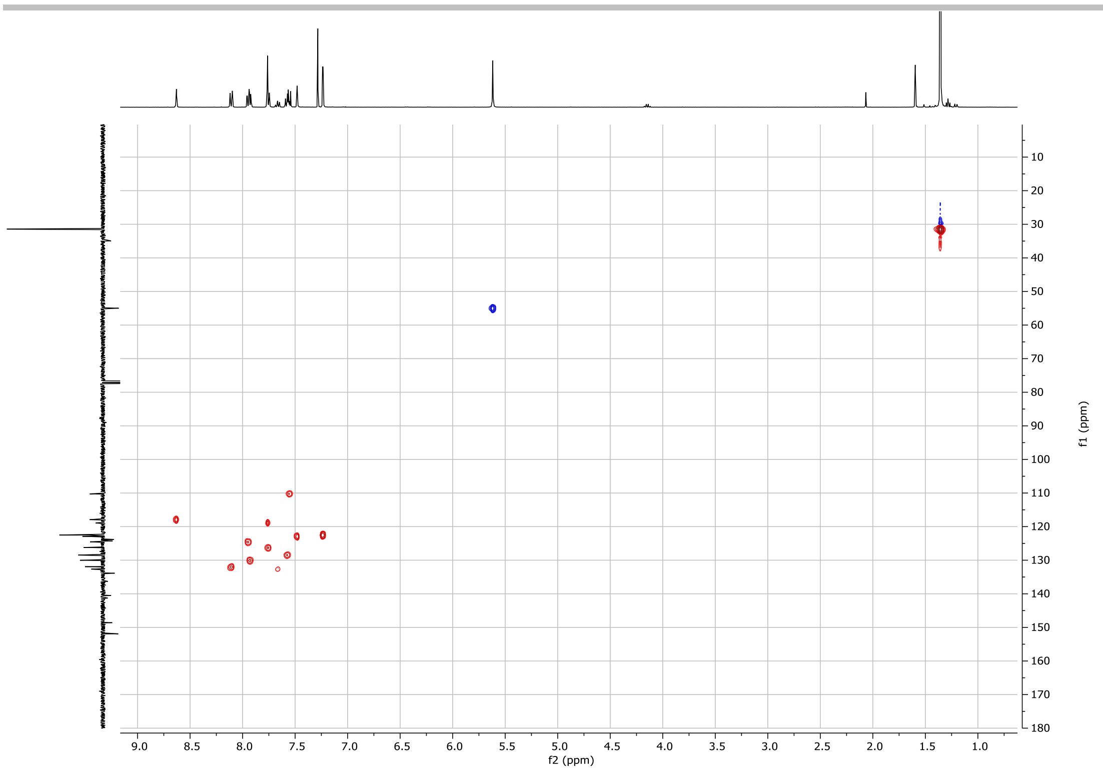

Figure S26. HSQC NMR (101 MHz, $CDCl_3$) of **1**.

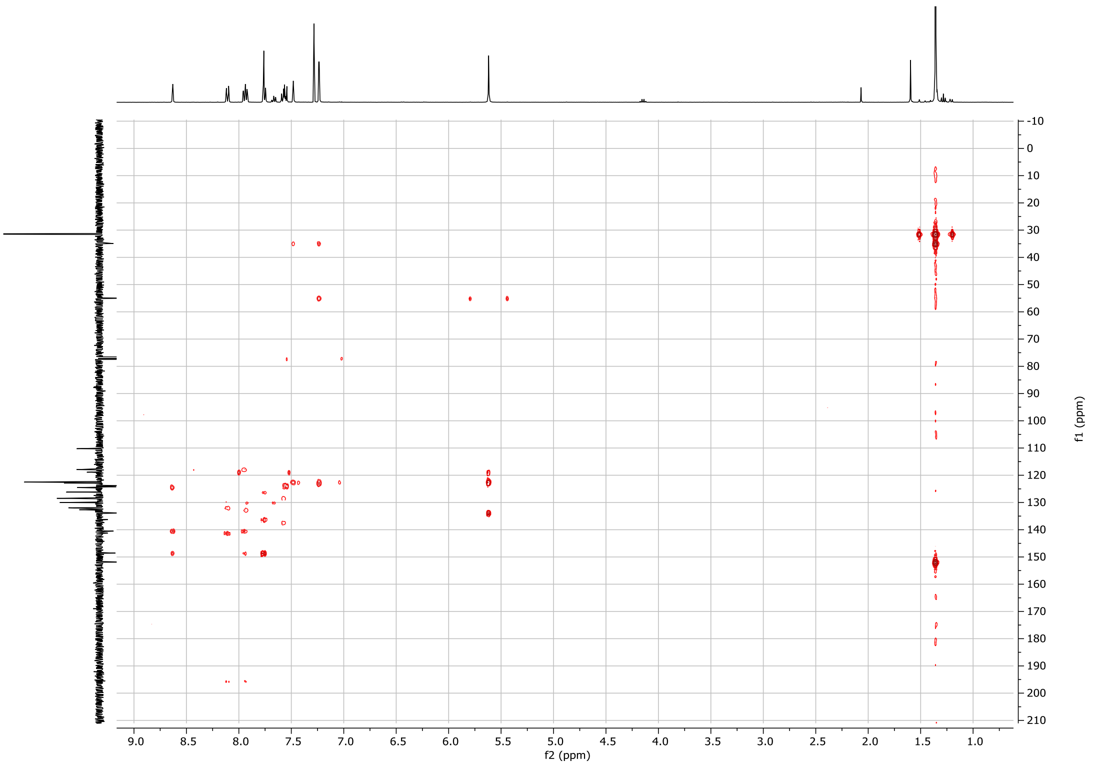

Figure S27. HMBC NMR (101 MHz, $CDCl_3$) of **1**.

Synthesis of [2]rotaxane 1⊂2

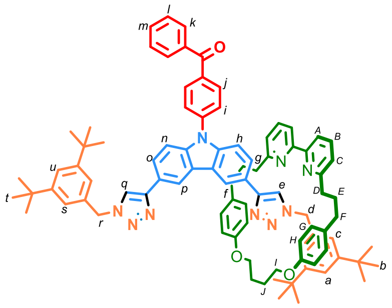

To a solution of **S7** (78.5 mg, 0.122 mmol), **S1** (31.2 mg, 0.127 mmol), **S2** (70.3 mg, 0.147 mmol) and $[Cu(NCMe)_4]PF_6$ (47.7 mg, 0.128 mmol) in dry CH_2Cl_2 (3.7 mL) under a nitrogen atmosphere was added DIPEA (160 µL, 0.953 mmol) and the solution was stirred at r.t. for 16 h. After removing the solvent *in vacuo*, the crude was purified by column chromatography on neutralised SiO_2 ($CHCl_3$, with a 0-25% MeCN gradient). After analysis by ESI-LCMS, the fractions containing the Cu triazolide of **1**⊂**2** were combined and the solvent removed *in vacuo*. The Cu triazolide of **1**⊂**2** was dissolved in CH_2Cl_2 (5 mL), TFA (280 µL, 3.21 mmol) was added and the solution stirred at r.t. for 3 h. The solution was poured onto an EDTA-NH_3 solution (30 mL) and the organic material extracted with CH_2Cl_2 (3 x 50 mL), dried with $MgSO_4$, filtered and the solvent removed *in vacuo*. Compound **1**⊂**2** was obtained as a yellow gum (106.6 mg, 64%). ^{1}H NMR (500 MHz, $CDCl_3$) δ_H 9.29 (s, 1H, H_q), 8.27-8.21 (br. m, 1H, H_p), 8.08 (app. d, *J* = 8.7, 2H, H_j), 8.03-7.95 (m, 2H, H_f, H_g), 7.95-7.89 (m, 2H, H_k), 7.72 (app. d, *J* = 8.5, 2H, H_i), 7.67-7.62 (m, 1H, H_m), 7.61 (s, 1H, H_e), 7.59-7.50 (m, 6H, H_B, H_h, H_l, H_o), 7.50-7.46 (m, 3H, H_a, H_C), 7.36-7.32 (br. m, 1H, H_t), 7.24 (d, *J* = 1.8, 2H, H_c), 7.17-7.08 (m, 3H, H_n, H_s), 7.02 (d, *J* = 7.6, Hz, 2H, H_A), 6.41 (d, *J* = 8.6, 4H, H_H), 6.20 (d, *J* = 8.1 Hz, 4H, H_G), 5.62 (s, 2H, H_d), 4.85 (s, 2H, H_r),4.76-4.66 (m, 2H, 2 of H_I), 4.33-4.23 (m, 2H, 2 of H_I), 2.41-2.30 (m, 2H, 2 of H_J), 2.30-2.14 (m, 8H, H_D, H_F), 2.13-2.03 (m, 2H, 2 of H_J), 1.43-1.31 (m, 22H, H_b, H_E), 1.19 (s, 18H, H_u); ^{13}C NMR (126 MHz, $CDCl_3$) δ_C 195.6, 163.3, 157.5, 157.1, 152.0, 151.1, 149.0, 146.8, 141.9, 140.0, 139.4, 137.5, 136.8, 135.6, 134.5, 134.2, 132.6, 132.2, 131.9, 130.1, 128.5, 128.1, 125.8, 125.6, 124.9, 124.7, 123.4, 123.1, 123.1, 122.9, 122.9, 122.8, 122.4, 121.7, 119.7, 118.8, 118.1, 114.9, 109.8, 108.2, 66.4, 54.9, 54.1, 36.9, 35.0, 34.9, 34.7, 31.8, 31.5, 31.4, 25.1; HRMS (ESI+, MeCN): *m/z* = 683.5773 $[M+2H]^{2+}$, calc. 683.7703.

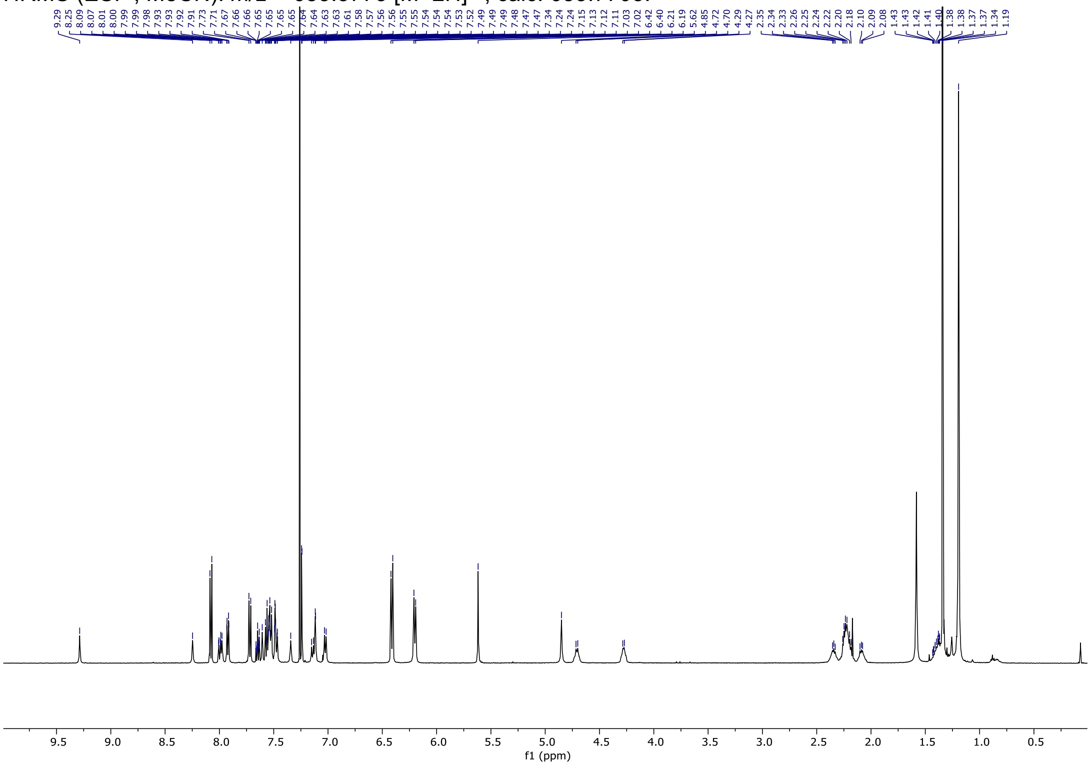

Figure S28. ^{1}H NMR (500 MHz, $CDCl_3$) of **1**⊂**2**.

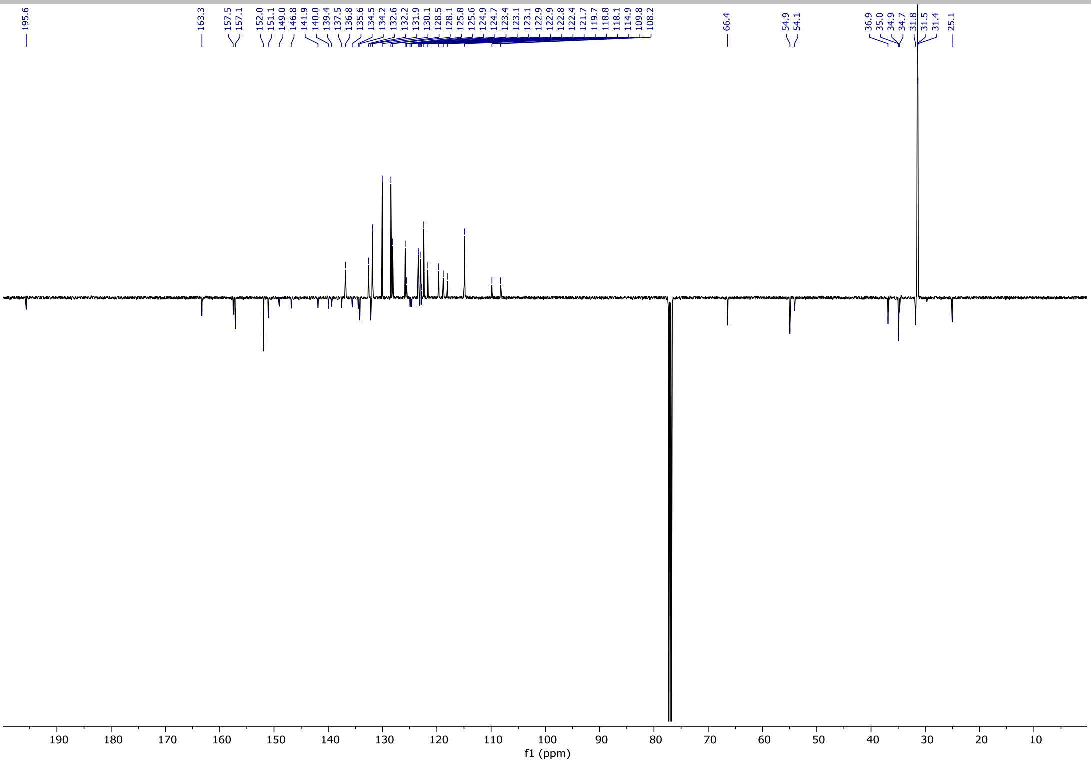

Figure S29. JMOD NMR (126 MHz, $CDCl_3$) of **1**⊂**2**.

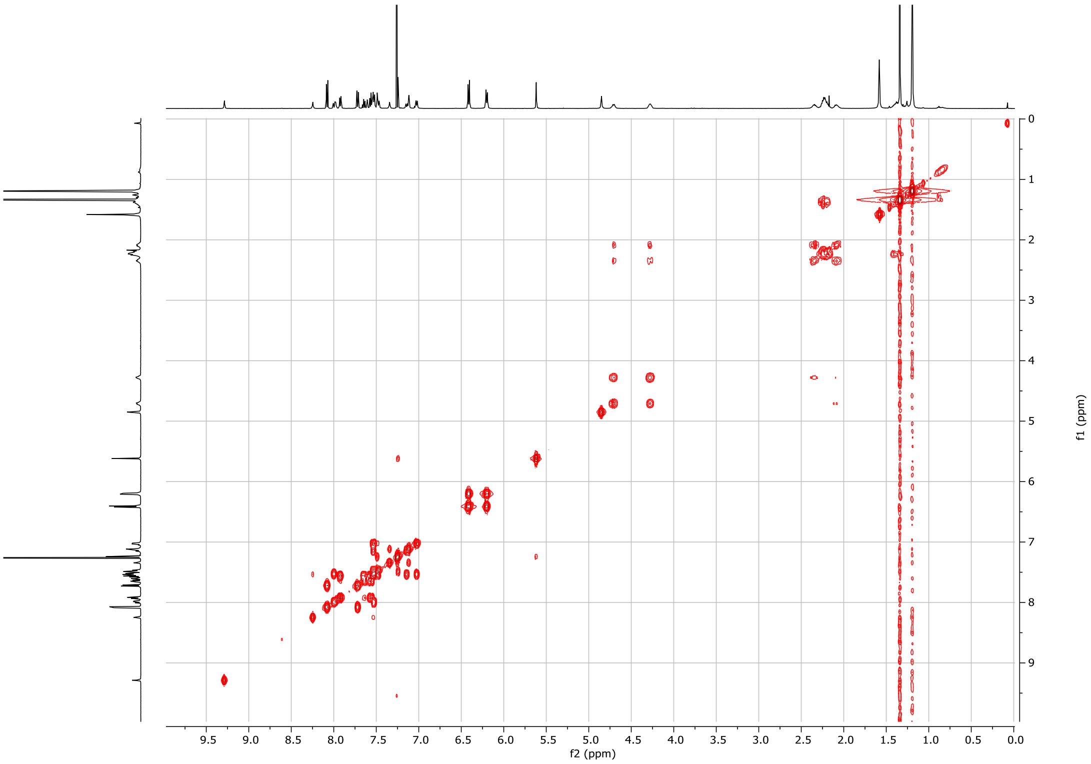

Figure S30. COSY NMR (500 MHz, $CDCl_3$) of **1**⊂**2**.

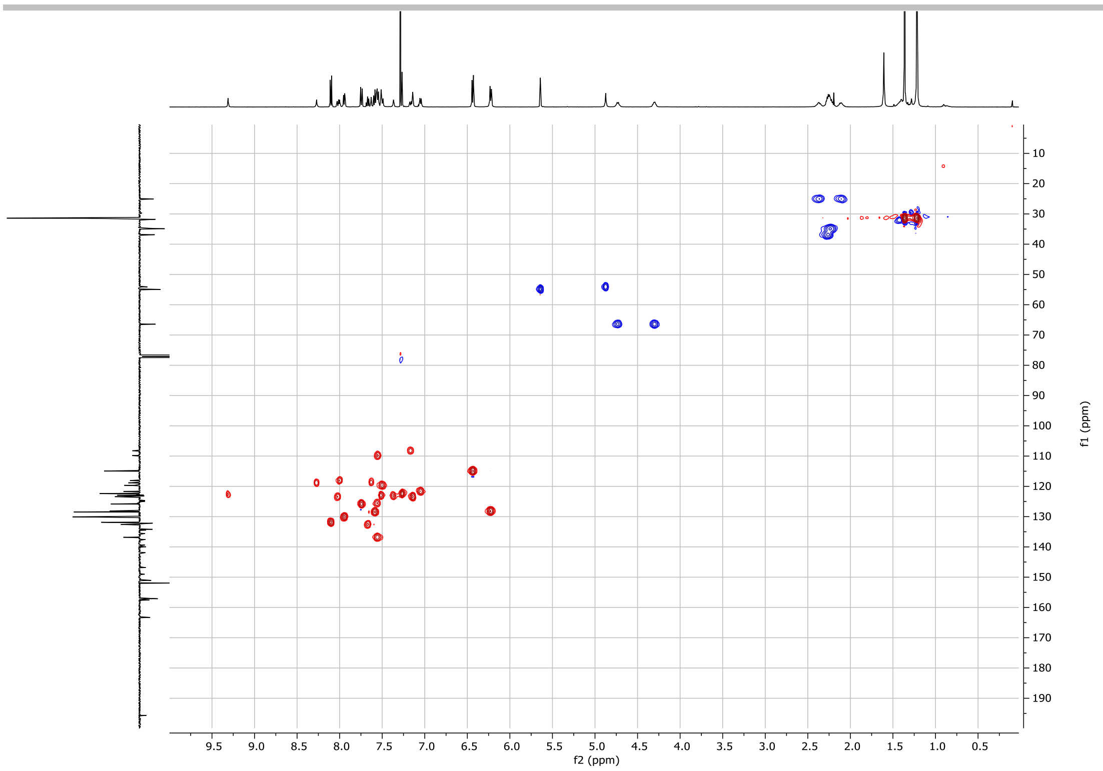

Figure S31. HSQC NMR (126 MHz, $CDCl_3$) of **1**⊂**2**.

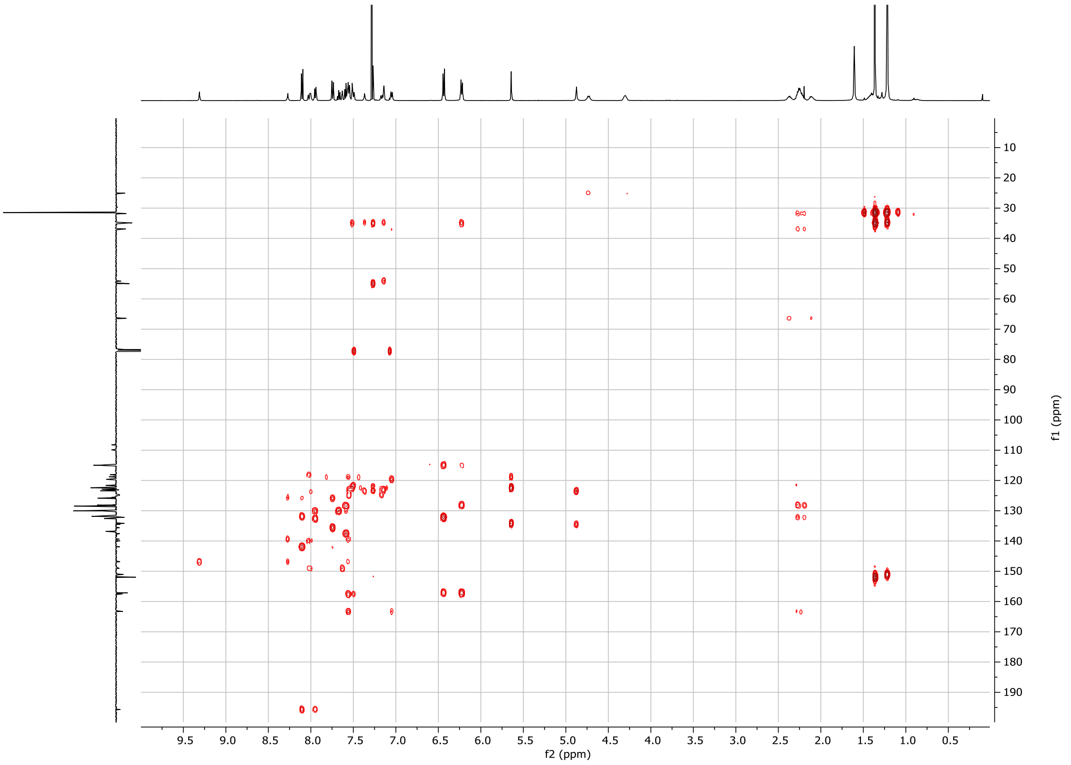

Figure S32. HMBC NMR (126 MHz, $CDCl_3$) of **1**⊂**2**.

Synthesis of [3]rotaxane 1⊂2$_2$

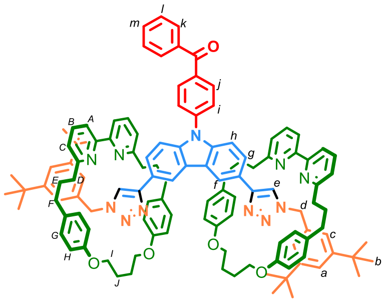

To a solution of **S6** (30.6 mg, 0.0774 mmol), **S1** (37.9 mg, 0.154 mmol), **S2** (74.3 mg, 0.155 mmol) and $[Cu(NCMe)_4]PF_6$ (49.9 mg, 0.134 mmol) in dry CH_2Cl_2 (3.9 mL) under a nitrogen atmosphere was added DIPEA (105 µL, 0.625 mmol) and the solution was stirred at r.t. for 16 h. After removing the solvent *in vacuo*, the crude was purified *via* column chromatography on neutralised SiO_2 ($CHCl_3$, with a 0-25% MeCN gradient). After analysis by ESI-LCMS, the fractions containing the Cu triazolide of **1**⊂**2**$_2$ were combined and the solvent removed *in vacuo*. The Cu triazolide of **1**⊂**2**$_2$ was dissolved in CH_2Cl_2 (2.5 mL), TFA (100 µL, 1.30 mmol) was added and the solution stirred at r.t. for 3 h. The solution was poured over an EDTA-NH_3 solution (25 mL) and the organic material extracted with CH_2Cl_2 (3 x 50 mL), dried with $MgSO_4$, filtered and the solvent removed *in vacuo*. Compound **1**⊂**2**$_2$ was obtained as a yellow gum (81.6 mg, 57%). ^{1}H NMR (400 MHz, $CDCl_3$) δ_H 9.14 (s, 2H, H_e), 8.36 (d, *J* = 1.6 Hz, 2H, H_f), 8.06 (d, *J* = 8.6, 2H, H_j), 7.97-7.88 (m, 2H, H_k), 7.73-7.64 (m, 7H, H_B, H_i, H_m), 7.62 (dd, *J* = 7.9, 1.1, 4H, H_C), 7.58-7.54 (m, 2H, H_l), 7.40-7.36 (m, 4H, H_a, H_g), 7.17-7.13 (m, 6H, H_c, H_h), 7.08 (dd, *J* = 7.7, 1.1, 4H, H_A), 6.49 (d, *J* = 8.6, 8H, H_H), 6.36 (d, *J* = 8.5, 8H, H_G), 4.59-4.51 (m, 8H, H_d, 4 of H_I), 4.38-4.31 (m, 4H, 4 of H_I), 2.44-2.38 (m, 8H, H_D), 2.35-2.27 (m, 8H, H_F), 2.23-2.17 (br m, 8H, H_J), 1.58-1.51 (m, 8H, H_E), 1.26 (s, 36H, H_b); ^{13}C NMR (101 MHz, $CDCl_3$) δ_C 195.6, 163.0, 157.9, 157.5, 150.7, 145.9, 142.3, 139.2, 137.6, 136.8, 135.3, 134.4, 132.5, 132.2, 131.7, 130.0, 128.4, 128.3, 125.8, 125.3, 125.1, 124.2, 124.1, 122.9, 122.5, 121.6, 120.0, 118.0, 115.3, 108.6, 67.0, 53.6, 37.0, 34.8, 34.8, 31.8, 31.5, 25.1; HRMS (ESI+, MeCN): *m/z* = 922.9255 $[M+2H]^{2+}$, calc. 922.5257.

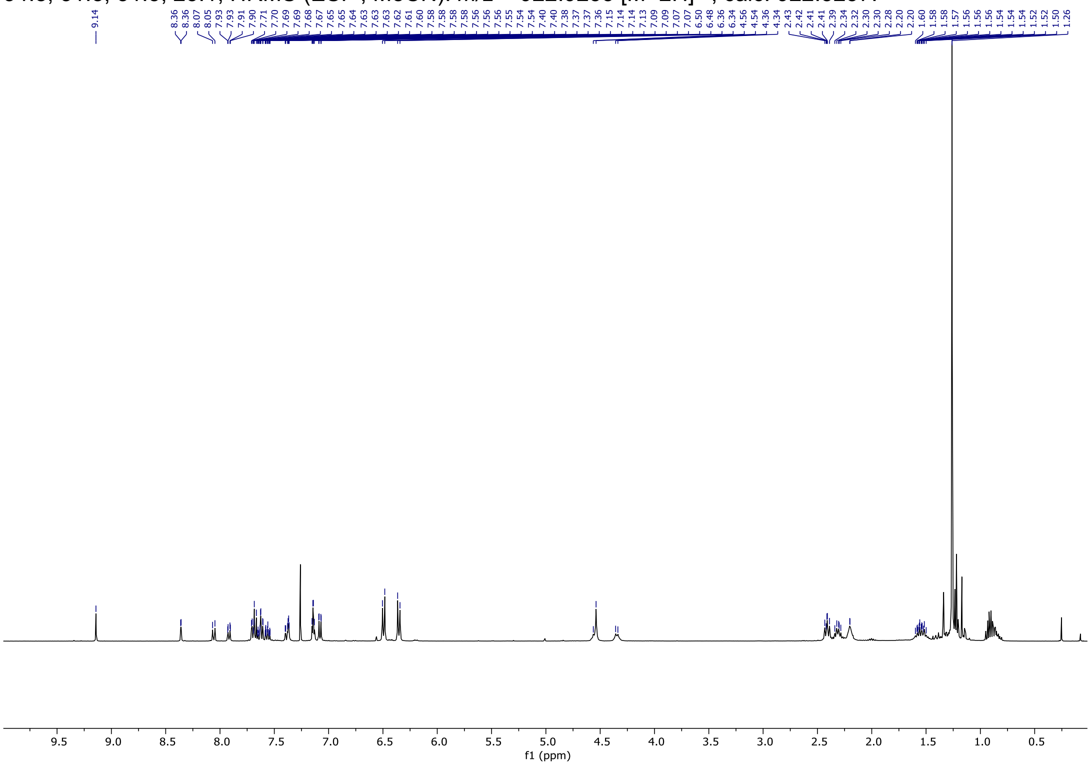

Figure S33. ^{1}H NMR (500 MHz, $CDCl_3$) of **1**⊂**2**$_2$.

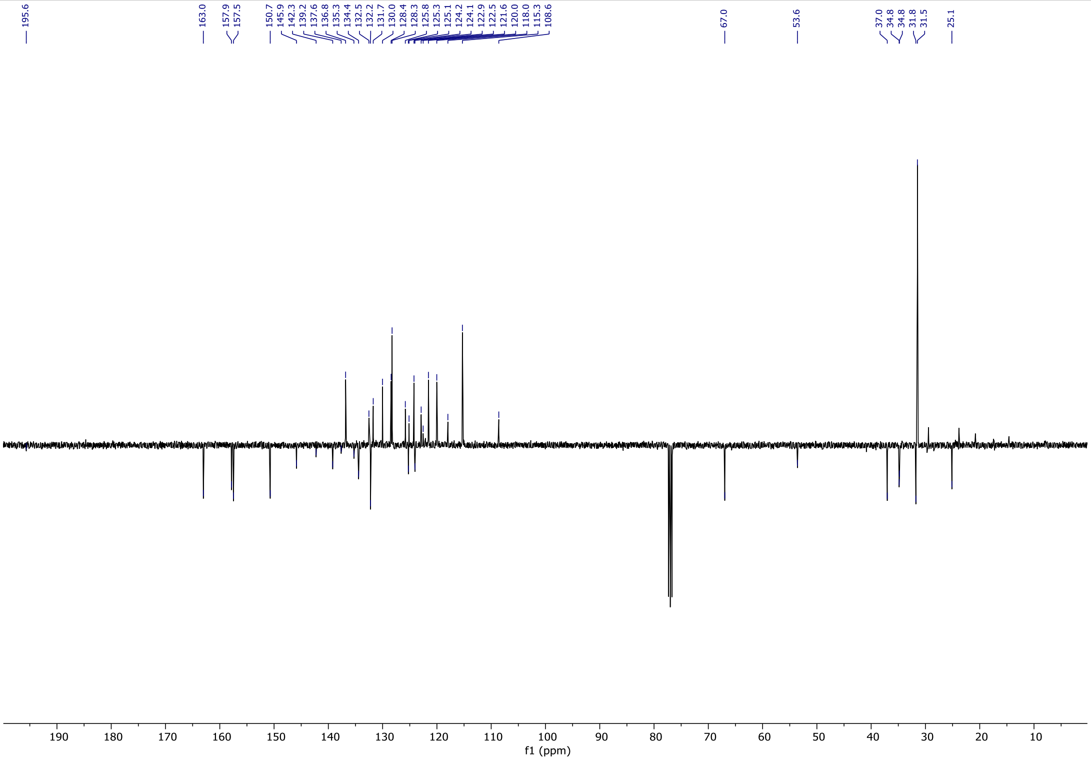

Figure S34. JMOD NMR (126 MHz, $CDCl_3$) of **1**⊂**2**$_2$.

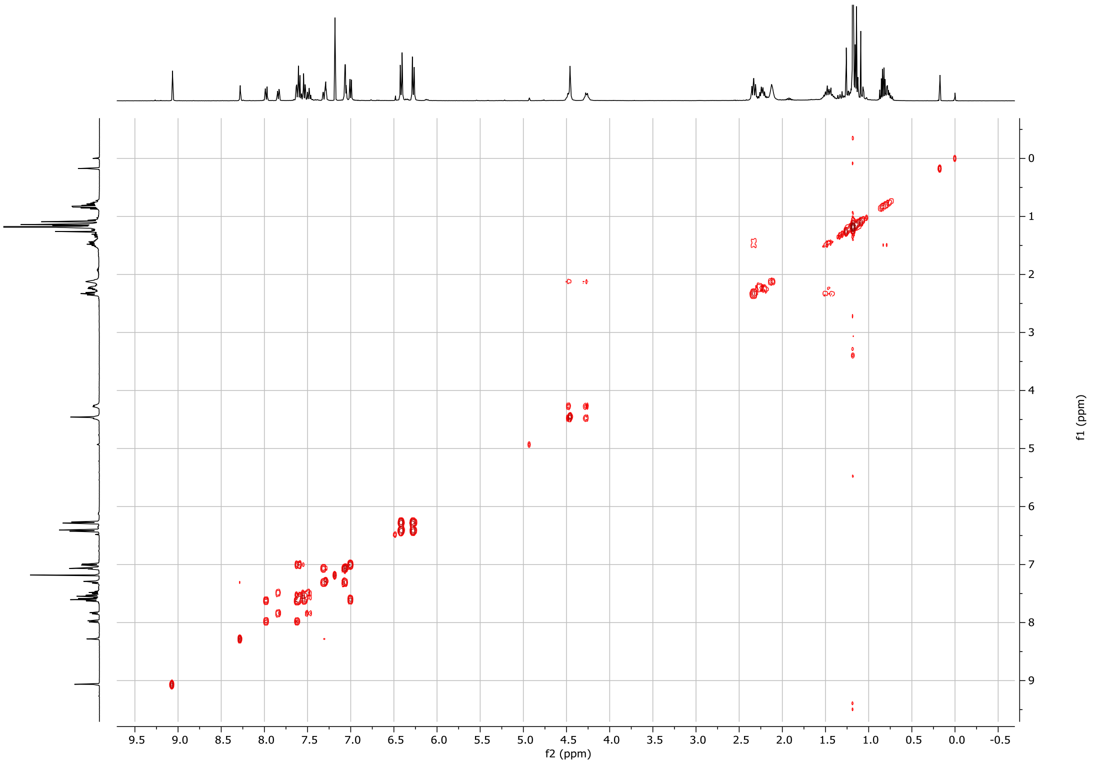

Figure S35. COSY NMR (500 MHz, $CDCl_3$) of **1**⊂**2**$_2$.

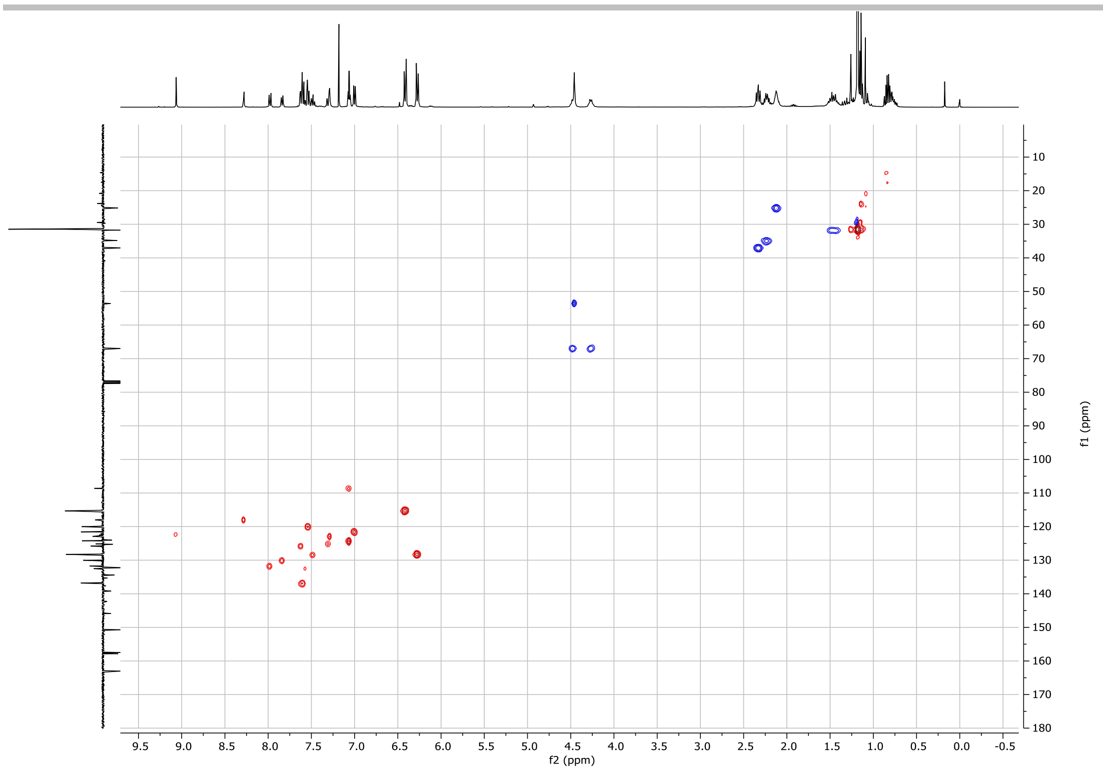

Figure S36. HSQC NMR (126 MHz, $CDCl_3$) of **1**⊂**2**$_2$.

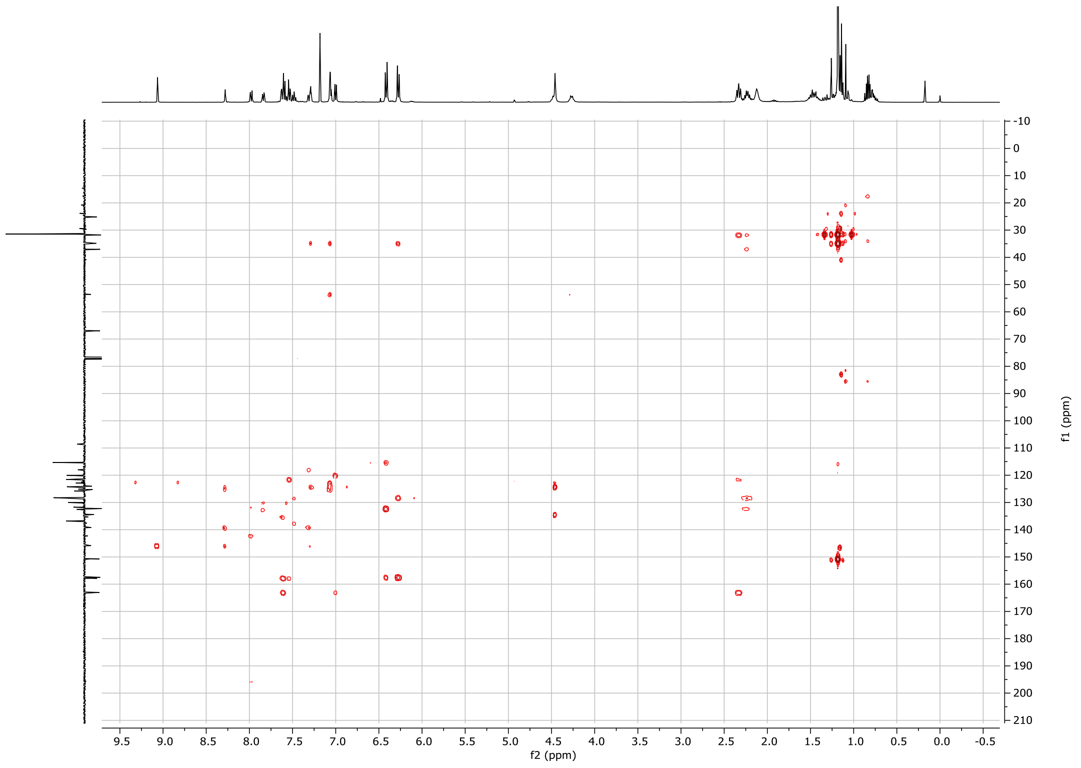

Figure S37. HMBC NMR (126 MHz, $CDCl_3$) of **1**⊂**2**$_2$.

Photostability measurement of the Emitters

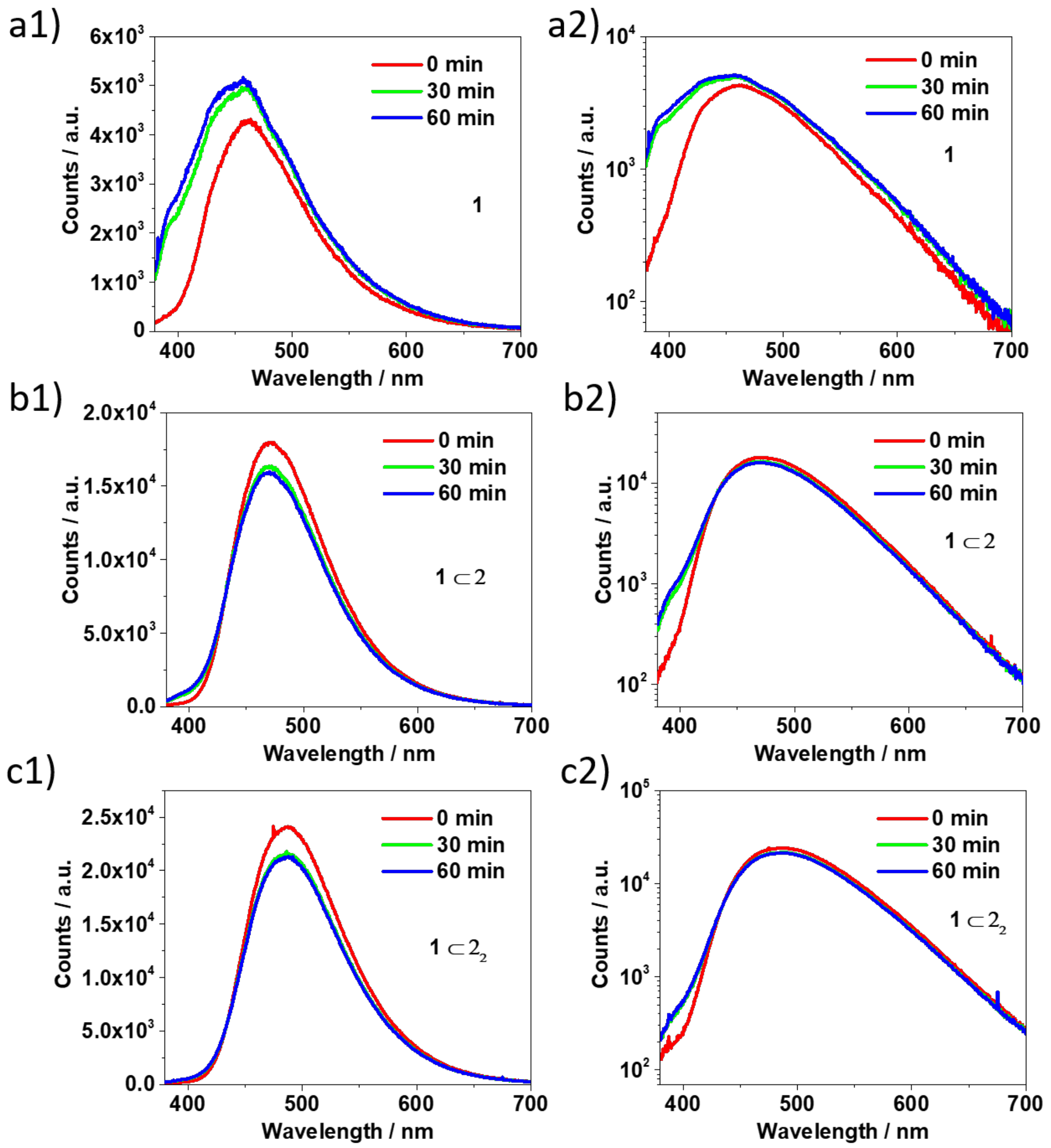

Figure S38 a1, b1, c1). PL spectra of **1**, **1⊂2** and **1⊂2₂** in toluene (10^{-5} M) 0 min, 30 min and 60 min after continuous photoexcitation plotted in linear scale. a2, b2, c2). The same spectra plotted on a semi-log scale. (Sample volume = 80 µL, λ_{exc} = 325 nm, optical power = 4.18 mW, beam diameter = 1.1 mm, beam intensity = 440 mW/cm^2).

Electrochemical Properties of the Emitters

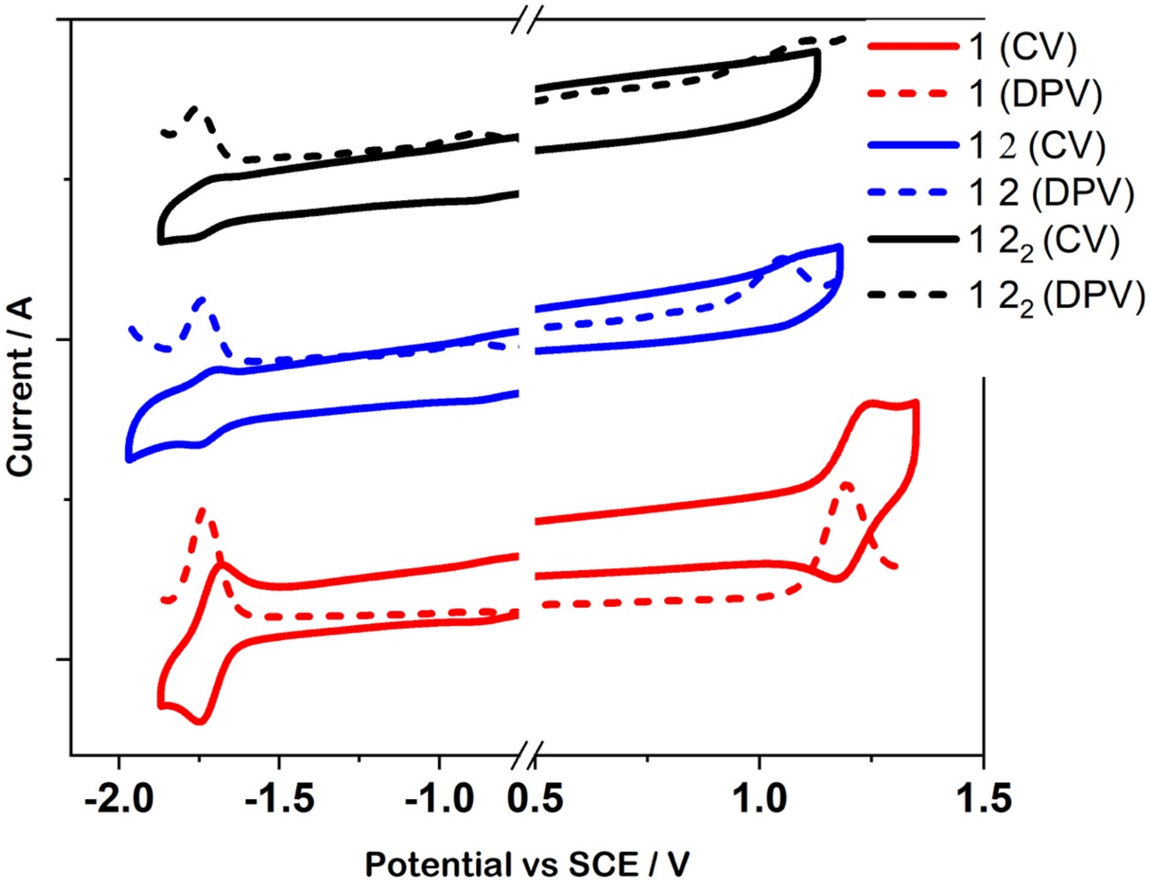

Figure S39. Cyclic Voltammograms and Differential Pulse Voltammograms of **1**, **1**⊂**2** and **1**⊂$\mathbf{2_2}$ in MeCN (10^{-3} M), reported versus SCE (Fc/Fc^+ = 0.38 V in MeCN) and scan rate = 100 mV/s.[9]

Photophysical Properties of the Emitters

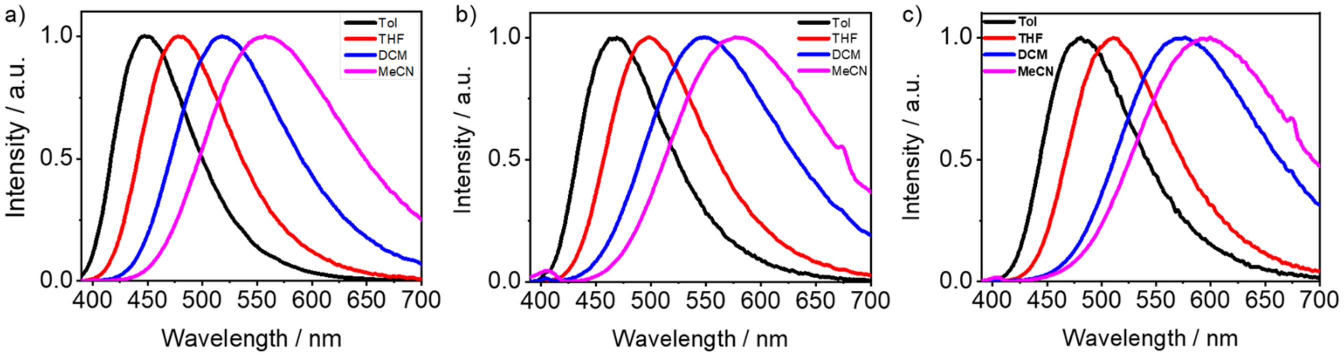

Figure S40. Solvatochromic PL spectra of a) **1**, b) **1**⊂**2** and c) **1**⊂**2**$_2$ (λ_{exc} = 340nm).

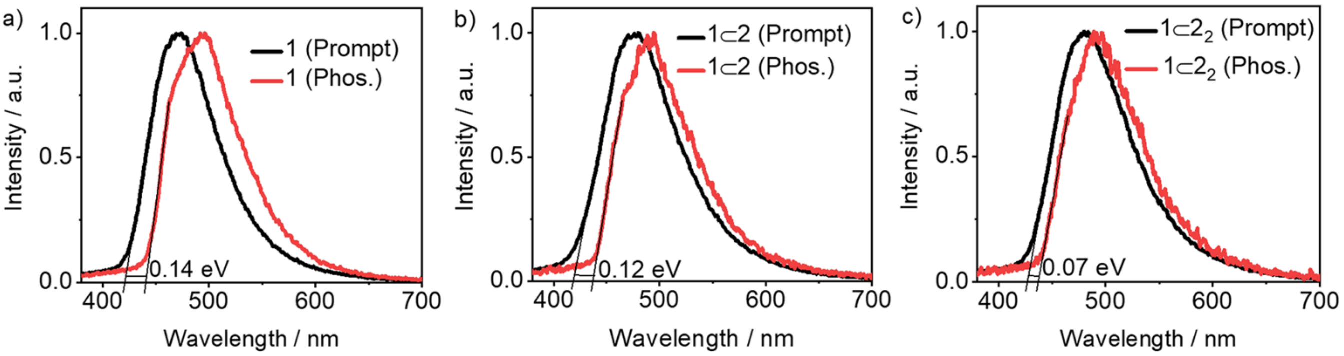

Figure S41. Prompt and phosphorescence spectra measured at 77 K in mCP (10 wt%) a) **1**, b) **1**⊂**2** and c) **1**⊂**2**$_2$ (λ_{exc} = 343 nm).

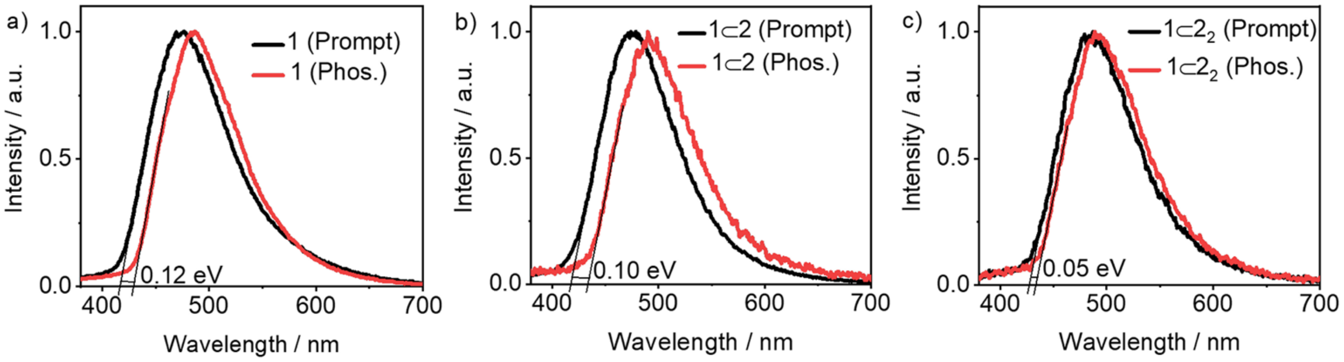

Figure S42. Prompt and phosphorescence spectra measured at 77 K in DPEPO (10 wt%) a) **1**, b) **1**⊂**2** and c) **1**⊂**2**$_2$ (λ_{exc} = 343 nm).

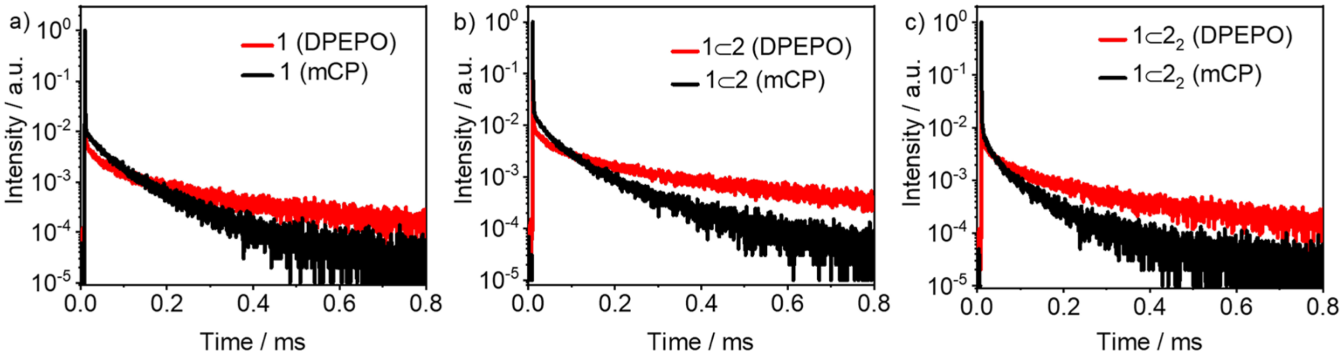

Figure S43. Time-resolved emission in DPEPO (10 wt%) and mCP (10 wt%) host a) **1**, b) **1**⊂**2** and c) **1**⊂**2**$_2$ (λ_{exc} = 378 nm).

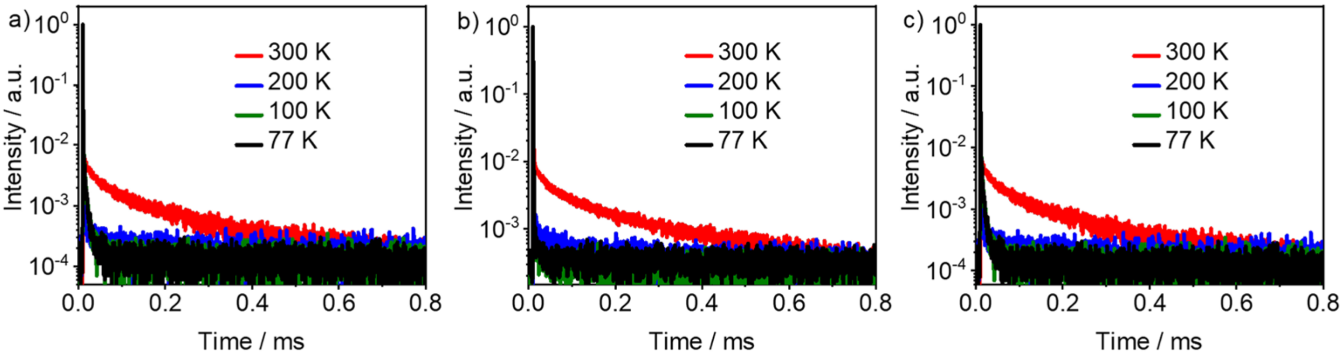

Figure S44. Time-resolved PL decay curve and measured at variable temperature in 10 wt% DPEPO film a) **1**, b) **1**⊂**2** and c) **1**⊂**2**$_2$ (λ_{exc} = 378 nm).

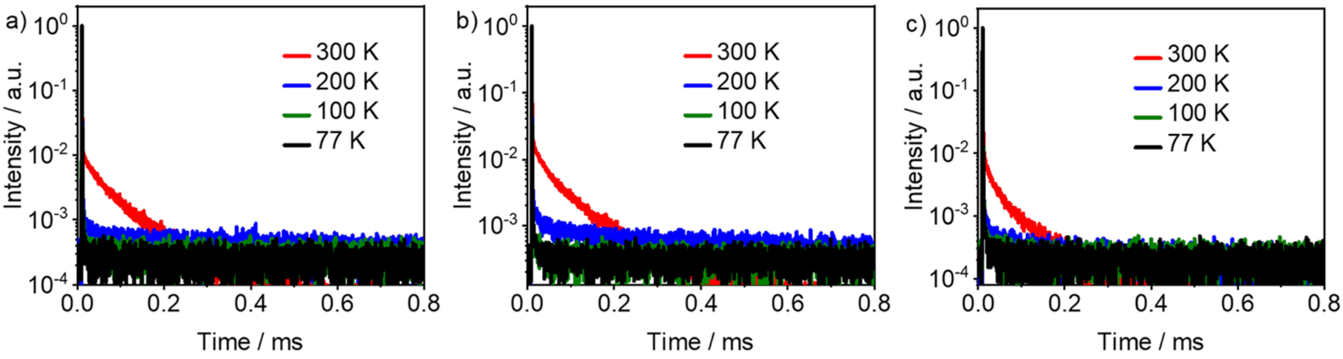

Figure S45. Time-resolved PL decay curve and measured at variable temperature in 10 wt% mCP film a) **1**, b) **1**⊂**2** and c) **12**$_2$ (λ_{exc} = 378 nm).

Computational Photophysical Properties of the Emitters

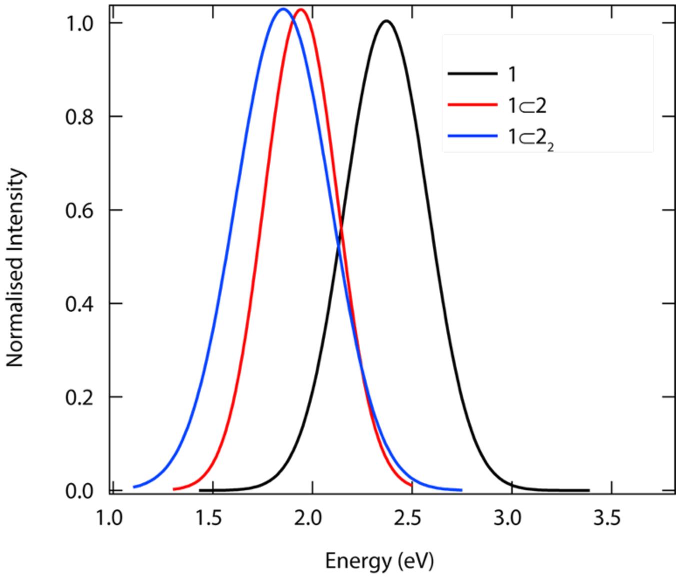

Figure S46. The emission spectra for **1**, **1**⊂**2** and **1**⊂**2**$_2$ computed from the MD simulations.

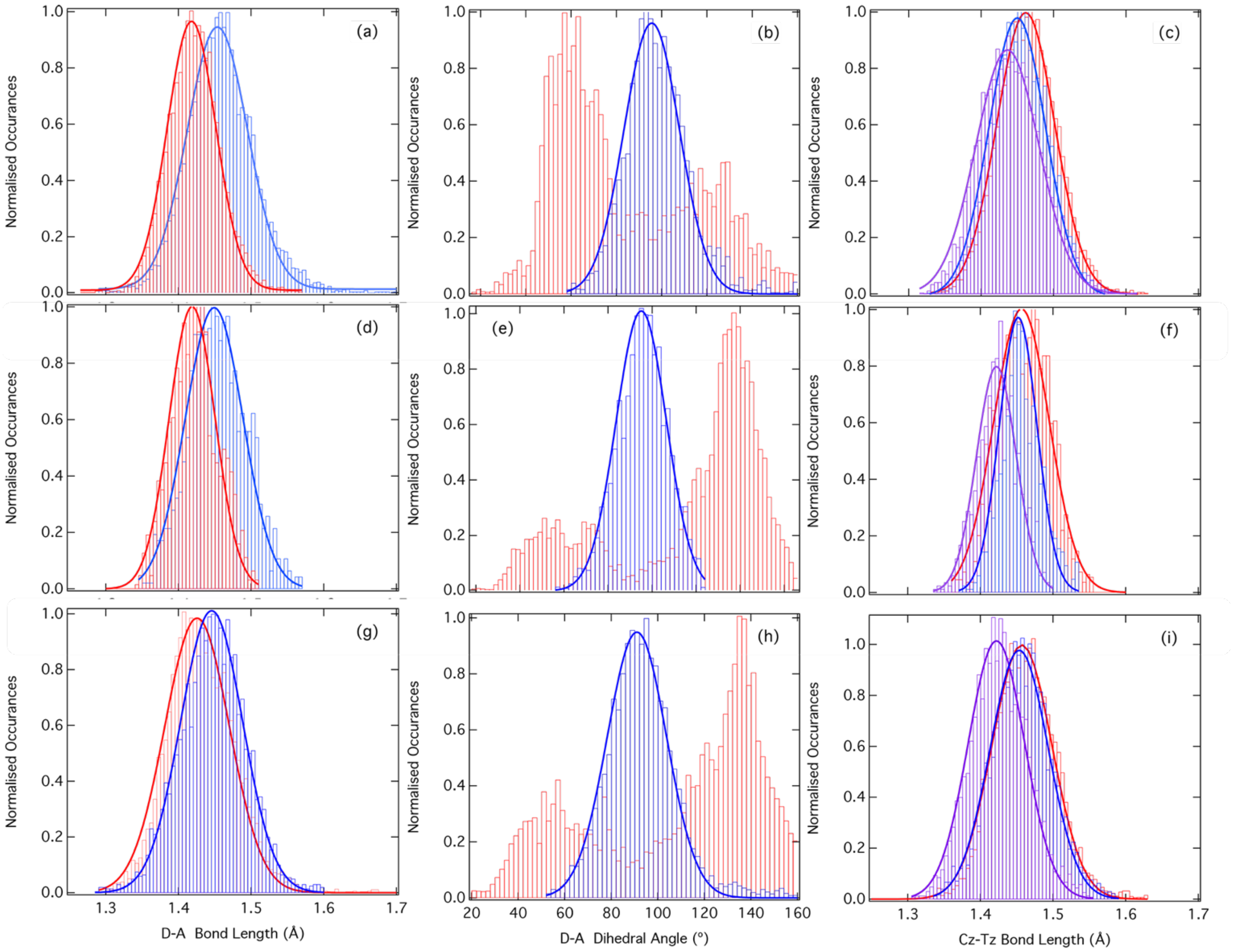

Figure S47. The distribution of the D-A bond distance (left), the dihedral angle between the D and A groups (middle) and carbazole triazole aryl-aryl bond (right) for (a-c) **1,** (d-f) **1**⊂**2** and (g-i) **1**⊂**2**$_2$ for the ground (red) and excited state (blue, purple) geometries.

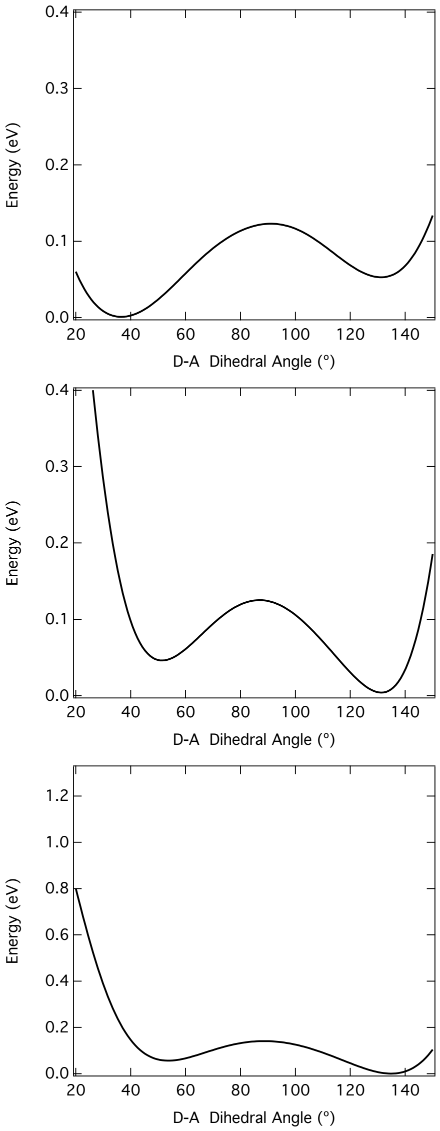

Figure S48. Ground state potential energy scans around the dihedral angle between the D and A groups for **1** (upper), **1**⊂**2** (middle) and **1**⊂$\mathbf{2}_2$ (lower).

Single crystal X-ray crystallographic data for 1⊂2 (CCDC: 2061107)

Single crystals of **1**⊂**2** were grown by layer diffusion of MeOH into a solution of the compound in CH_2Cl_2. Data were collected at 100 K using a FRE+ VHF diffractometer equipped with a HYPix6000 enhanced sensitivity detector. Cell determination, data collection, data reduction, cell refinement and absorption correction were performed with CrysalisPro. Using Olex2, the structure was solved with the SHELXT program using charge flipping,[13] and refined with the SHELXL refinement package.[13] H atoms were placed in calculated positions and refined using a riding model. Differences in the components of the anisotropic displacement parameters are the result of minor positional disorder that has not been modelled as doing so led to degradation in the overall quality of the solution.

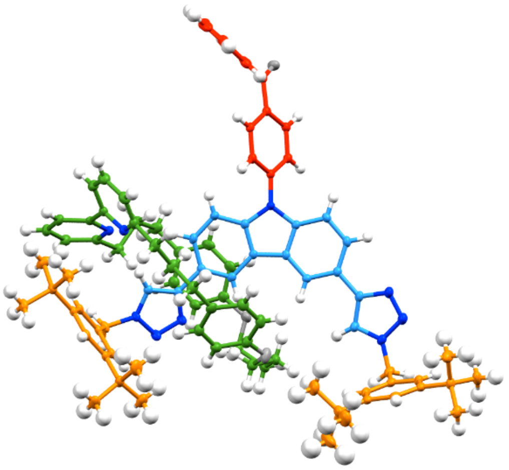

Figure S49. Single crystal X-ray structure of **1**⊂**2**. Ellipsoids shown at 50% probability.

Table S1. T Crystal data and structure refinement for **1**⊂**2**.

Identification code	CCDC: 2061107
Empirical formula	$C_{91}H_{97}N_9O_3$
Formula weight	926.21
Temperature/K	100(2)
Crystal system	monoclinic
Space group	$P2_1/n$
a/Å	16.7366(5)
b/Å	14.4586(3)
c/Å	31.8111(6)
α/°	90
β/°	93.707(2)
γ/°	90
Volume/Å^3	7681.8(3)
Z	1
ρ_{calc}g/cm^3	1.096
μ/mm^{-1}	0.069
F(000)	2533.0
Crystal size/mm^3	0.18 × 0.03 × 0.015
Radiation	MoKα (λ = 0.71073)
2Θ range for data collection/°	3.726 to 62.26
Index ranges	$-22 \le h \le 21$, $-15 \le k \le 20$, $-37 \le l \le 44$
Reflections collected	77599
Independent reflections	20856 [R_{int} = 0.0505, R_{sigma} = 0.0755]
Data/restraints/parameters	20856/0/940
Goodness-of-fit on F^2	1.036
Final R indexes [I>=2σ (I)]	R_1 = 0.0624, wR_2 = 0.1511
Final R indexes [all data]	R_1 = 0.1227, wR_2 = 0.1773
Largest diff. peak/hole / e Å^{-3}	0.49/-0.28

Carbazole purchased from Sigma (≥ 95%)

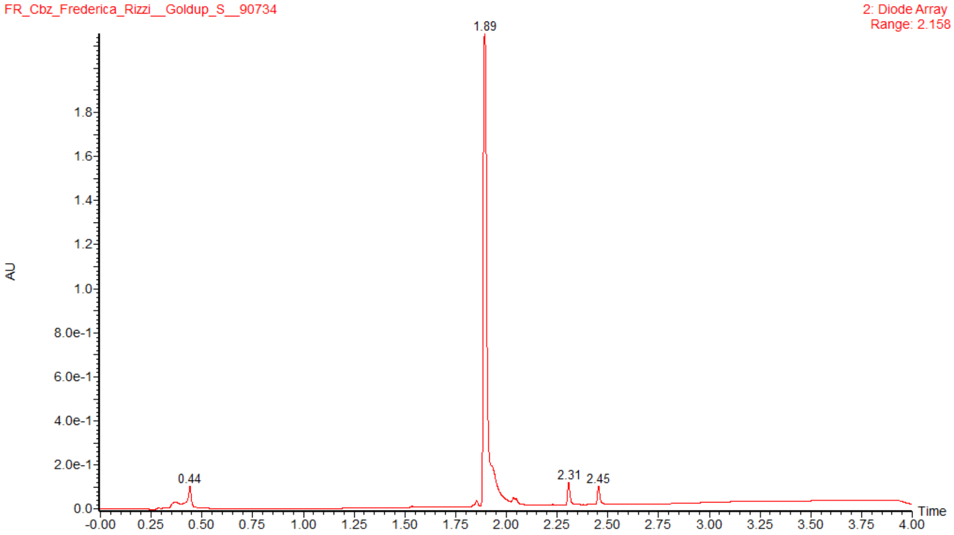

Figure S50. RP-UHPLC of carbazole starting material.

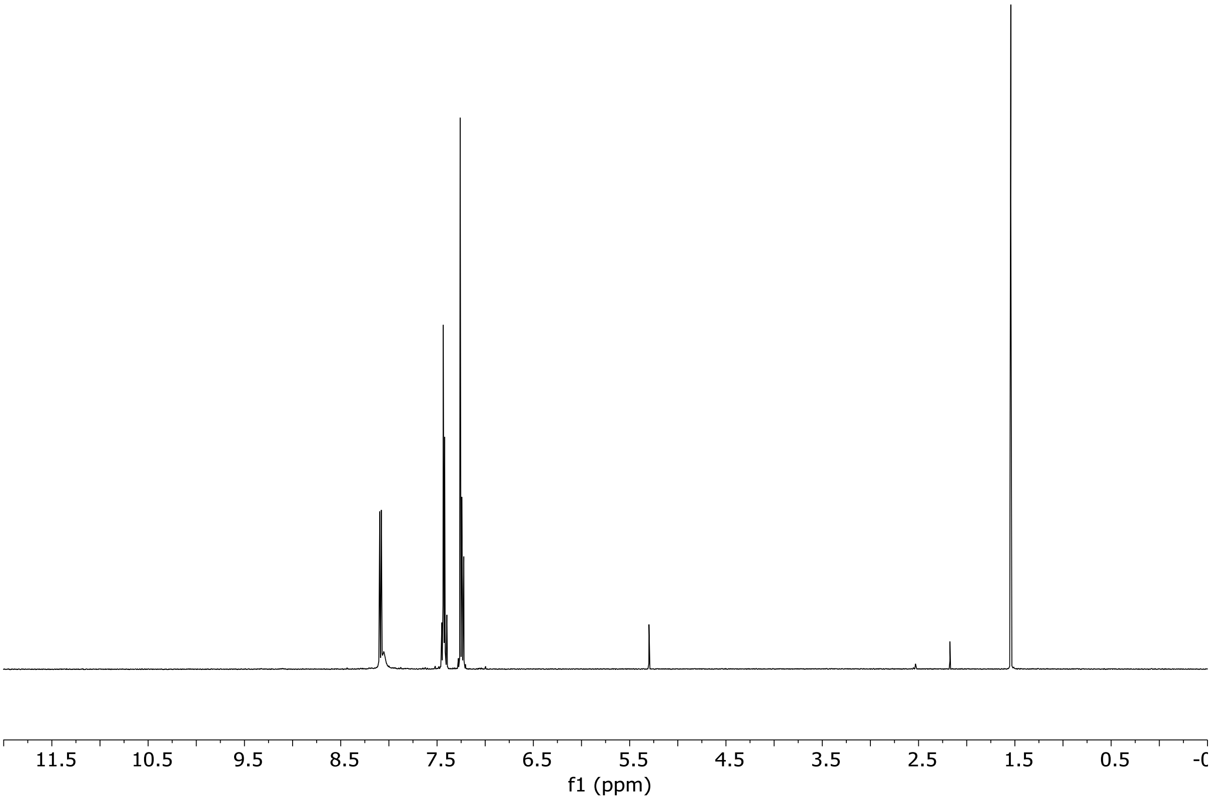

Figure S51. ^{1}H NMR of carbazole in $CDCl_3$.

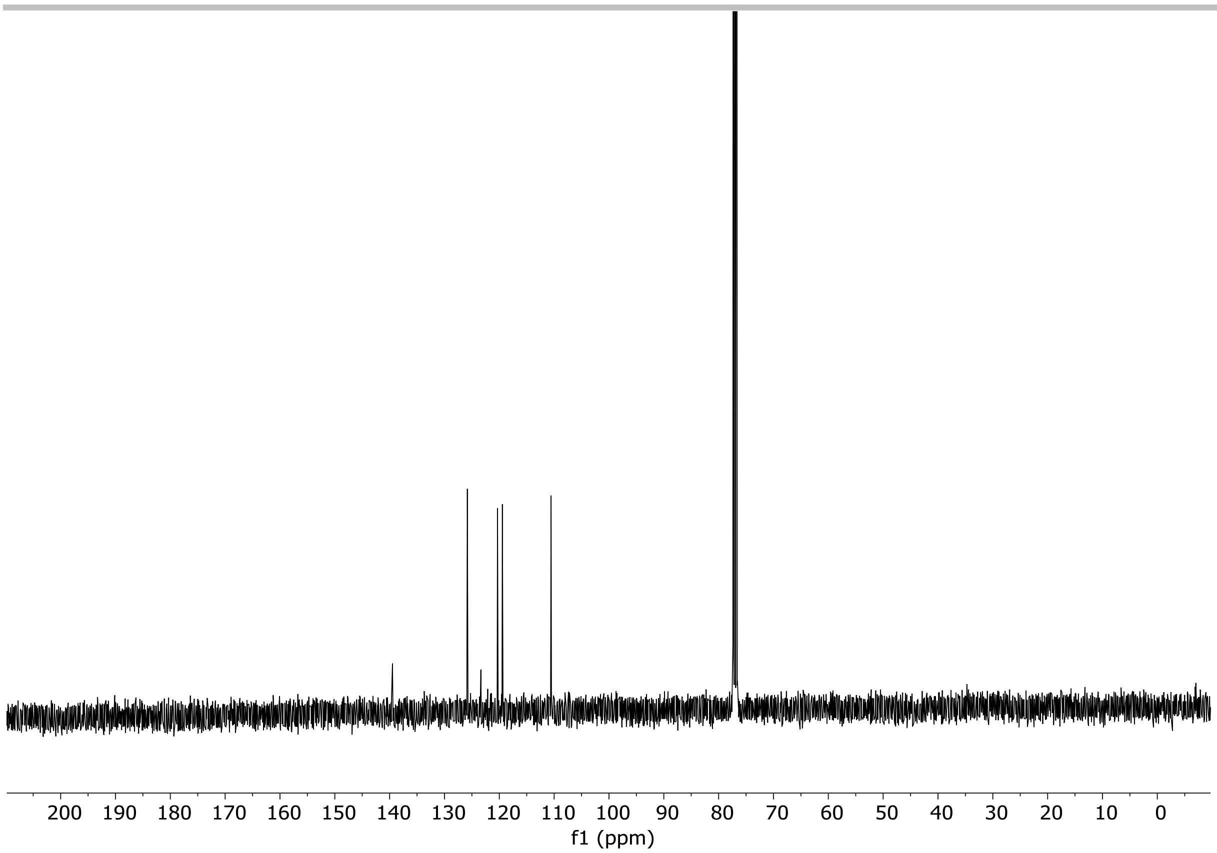

Figure S53. ^{13}C NMR of carbazole in $CDCl_3$

References

[1] A. Pigorsch, M. Köckerling, *Crystal Growth & Design* **2016**, *16*, 4240–4246.

[2] Y. Shao, Z. Gan, E. Epifanovsky, A. T. B. Gilbert, M. Wormit, J. Kussmann, A. W. Lange, A. Behn, J. Deng, X. Feng, D. Ghosh, M. Goldey, P. R. Horn, L. D. Jacobson, I. Kaliman, R. Z. Khaliullin, T. Kuś, A. Landau, J. Liu, E. I. Proynov, Y. M. Rhee, R. M. Richard, M. A. Rohrdanz, R. P. Steele, E. J. Sundstrom, H. L. W. III, P. M. Zimmerman, D. Zuev, B. Albrecht, E. Alguire, B. Austin, G. J. O. Beran, Y. A. Bernard, E. Berquist, K. Brandhorst, K. B. Bravaya, S. T. Brown, D. Casanova, C.-M. Chang, Y. Chen, S. H. Chien, K. D. Closser, D. L. Crittenden, M. Diedenhofen, R. A. D. Jr, H. Do, A. D. Dutoi, R. G. Edgar, S. Fatehi, L. Fusti-Molnar, A. Ghysels, A. Golubeva-Zadorozhnaya, J. Gomes, M. W. D. Hanson-Heine, P. H. P. Harbach, A. W. Hauser, E. G. Hohenstein, Z. C. Holden, T.-C. Jagau, H. Ji, B. Kaduk, K. Khistyaev, J. Kim, J. Kim, R. A. King, P. Klunzinger, D. Kosenkov, T. Kowalczyk, C. M. Krauter, K. U. Lao, A. D. Laurent, K. V. Lawler, S. V. Levchenko, C. Y. Lin, F. Liu, E. Livshits, R. C. Lochan, A. Luenser, P. Manohar, S. F. Manzer, S.-P. Mao, N. Mardirossian, A. V. Marenich, S. A. Maurer, N. J. Mayhall, E. Neuscamman, C. M. Oana, R. Olivares-Amaya, D. P. O'Neill, J. A. Parkhill, T. M. Perrine, R. Peverati, A. Prociuk, D. R. Rehn, E. Rosta, N. J. Russ, S. M. Sharada, S. Sharma, D. W. Small, A. Sodt, T. Stein, D. Stück, Y.-C. Su, A. J. W. Thom, T. Tsuchimochi, V. Vanovschi, L. Vogt, O. Vydrov, T. Wang, M. A. Watson, J. Wenzel, A. White, C. F. Williams, J. Yang, S. Yeganeh, S. R. Yost, Z.-Q. You, I. Y. Zhang, X. Zhang, Y. Zhao, B. R. Brooks, G. K. L. Chan, D. M. Chipman, C. J. Cramer, W. A. G. III, M. S. Gordon, W. J. Hehre, A. Klamt, H. F. S. III, M. W. Schmidt, C. D. Sherrill, D. G. Truhlar, A. Warshel, X. Xu, A. Aspuru-Guzik, R. Baer, A. T. Bell, N. A. Besley, J.-D. Chai, A. Dreuw, B. D. Dunietz, T. R. Furlani, S. R. Gwaltney, C.-P. Hsu, Y. Jung, J. Kong, D. S. Lambrecht, W. Liang, C. Ochsenfeld, V. A. Rassolov, L. V. Slipchenko, J. E. Subotnik, T. V. Voorhis, J. M. Herbert, A. I. Krylov, P. M. W. Gill, M. Head-Gordon, *Molecular Physics* **2015**, *113*, 184–215.

[3] F. Weigend, R. Ahlrichs, *Phys. Chem. Chem. Phys.* **2005**, *7*, 3297–3305.

[4] C. Adamo, V. Barone, *J. Chem. Phys.* **1999**, *110*, 6158–6170.

[5] S. Hirata, M. Head-Gordon, *CHEM. PHYS. LETT.* **1999**, *314*, 291–299.

[6] S. Grimme, *Chemical Physics Letters* **1996**, *259*, 128–137.

[7] I. S. Ufimtsev, T. J. Martinez, *J. Chem. Theory Comput.* **2009**, *5*, 2619–2628.

[8] N. C. Greenham, I. D. W. Samuel, G. R. Hayes, R. T. Phillips, Y. A. R. R. Kessener, S. C. Moratti, A. B. Holmes, R. H. Friend, *Chemical Physics Letters* **1995**, *241*, 89–96.

[9] N. G. Connelly, W. E. Geiger, *Chem. Rev.* **1996**, *96*, 877–910.

[10] J. J. Gassensmith, L. Barr, J. M. Baumes, A. Paek, A. Nguyen, B. D. Smith, *Organic Letters* **2008**, *10*, 3343–3346.

[11] J. E. M. Lewis, R. J. Bordoli, M. Denis, C. J. Fletcher, M. Galli, E. A. Neal, E. M. Rochette, S. M. Goldup, *Chemical Science* **2016**, *7*, 3154–3161.

[12] C.-H. Chen, T.-H. Hu, T.-C. Huang, Y.-L. Chen, Y.-R. Chen, C.-C. Cheng, C.-T. Chen, *Chemistry – A European Journal* **2015**, *21*, 17379–17390.

[13] G. M. Sheldrick, *Acta Cryst C* **2015**, *71*, 3–8.
